# Biomimetic assembly to superplastic metal–organic framework aerogels for hydrogen evolution from seawater electrolysis

**DOI:** 10.1002/EXP.20210021

**Published:** 2021-09-23

**Authors:** Yuntong Sun, Shuaishuai Xu, César A Ortíz‐Ledón, Junwu Zhu, Sheng Chen, Jingjing Duan

**Affiliations:** ^1^ Key Laboratory for Soft Chemistry and Functional Materials School of Chemical Engineering School of Energy and Power Engineering Nanjing University of Science and Technology Nanjing Jiangsu China; ^2^ Department of Chemistry University of Wisconsin–Madison Madison Wisconsin USA

**Keywords:** aerogels, electrocatalysis, hydrogen evolution reaction, metal–organic frameworks, superplasticity

## Abstract

Applications for metal–organic frameworks (MOFs) demand their assembly into three‐dimensional (3D) macroscopic architectures. The capability of sustaining structural integrity with considerable deformation is important to allow a monolithic material to work reliably. Nevertheless, it remains a significant challenge to introduce superplasticity in 3D MOF networks. Here, we report a general procedure for synthesizing 3D superplastic MOF aerogels inspired by the hierarchical architecture of natural corks. The resultant MOFs exhibited excellent superplasticity that can recover fully and rapidly to its original dimension after 50% strain compression and unloading for >2000 cycles. The 3D superplastic architecture is achieved by successively assembling one‐dimensional (1D) to two‐dimensional (2D) and then 3D, in a variety of MOFs with different transition metal active sites (Co‐, NiMn‐, NiCo‐, NiCoMn‐) and organic ligands (2‐thiophenecarboxylic acid and glutaric acid). Latent applications have been demonstrated for NiMn‐MOF aerogels to serve as a new generation of flexible electrocatalysts for hydrogen evolution reaction (HER) from seawater splitting, which requires a low overpotential of 243 mV to achieve a current density of 10 mA·cm^−2^. Notably, the electrocatalyst remains stable even being deformed, as the overpotential to achieve a current density of 10 mA·cm^−2^ increases slightly to 270, 264, and 258 mV after one‐, two‐, and threefold, respectively. In great contrast, traditional MOF powder‐electrodes demonstrate significant activity decay under similar conditions. This work opens up enormous opportunities for exploring new applications of MOFs in a freestanding, structurally adaptive, and macroscopic form.

## Graphical abstract

Superplastic metal–organic framework (MOF) assembled macroscopic architecture is reported for the first time, which can fully and rapidly recover to its initial states after 50% strain compression and unloading for 2000 cycles. The facile synthetic strategy has been extendable to many other single‐, binary‐, and ternary‐metal MOF assemblies. The NiMn‐MOF aerogel could serve as a novel class of flexible electrode for efficient hydrogen evolution reaction (HER) from natural seawater.

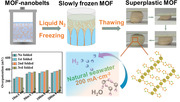

## 1 INTRODUCTION

Metal–organic frameworks (MOFs) represent a class of materials with an extraordinary combination of porous, structural, and chemical properties,^[^ ^1^, ^2^, ^3^, ^4^ ^]^ which can serve as nanoscale building blocks to construct macroscopic assemblies for a wide range of applications.^[^ ^5^, ^6^, ^7^, ^8^, ^9^ ^]^ Strategies, such as hybridization^[^ ^6^, ^7^ ^]^ and gelation,^[^ ^8^, ^9^, ^10^ ^]^ have been recently developed to fabricate three‐dimensional (3D) MOF aerogels. However, as‐resultant MOF aerogels are brittle, only bearing small recoverable deformation before failure. Superplasticity, an important mechanical property for many applications,^[^ ^11^ ^]^ has not been developed for MOFs. The capability of sustaining structural integrity during considerable deformation for 3D MOF assemblies is crucial, not only for fabricating flexible energy devices,^[^ ^2^, ^12^ ^]^ but also for designing MOF‐based membranes, gas storage systems, and biological tissues resistant for mechanical damping.^[^ ^5^, ^13^ ^]^

In nature, corks have been recognized as one of the oldest materials exploited by human beings because of their exceptional mechanical robustness.^[^ ^14^, ^15^, ^16^ ^]^ In corks microstructure, one‐dimensional (1D) nanofibers of cell walls intimately align in a highly oriented manner to reach reasonable strength. Further, an individual cell of tens of micrometers in size intricately connects with each other to produce a 3D honeycomb‐like architecture, which is helpful to maximize bulk superplastic modulus. Accordingly, we deduce that highly ordered assembly in the hierarchical microstructure is the key for corks to achieve high mechanical elasticity.

Inspired by the corks microstructure, we have fabricated superplastic 3D MOF architectures by a two‐step strategy, starting with synthesis of 1D MOF nanobelts followed by ice‐template‐driven assembly into 3D hierarchical aerogels. Note that a key step is to freeze MOF nanobelts in aqueous solutions, where a phase transformation from water to ice can force MOF nanobelts to move along the direction of ice solidification. As a result, the obtained MOFs display superplasticity during significant strain compression and unloading. Further, this general methodology can be readily extended to a variety of MOFs for potential applications in flexible energy storage and conversion devices. In this case, we tested the electrocatalytic activity of superplastic 3D NiMn‐MOF aerogels for hydrogen evolution reaction (HER) which displayed extraordinary activity and stability from seawater splitting.

## 2 RESULTS AND DISCUSSION

### 2.1 Synthesis of superplastic 3D MOF aerogels

**FIGURE 1:**
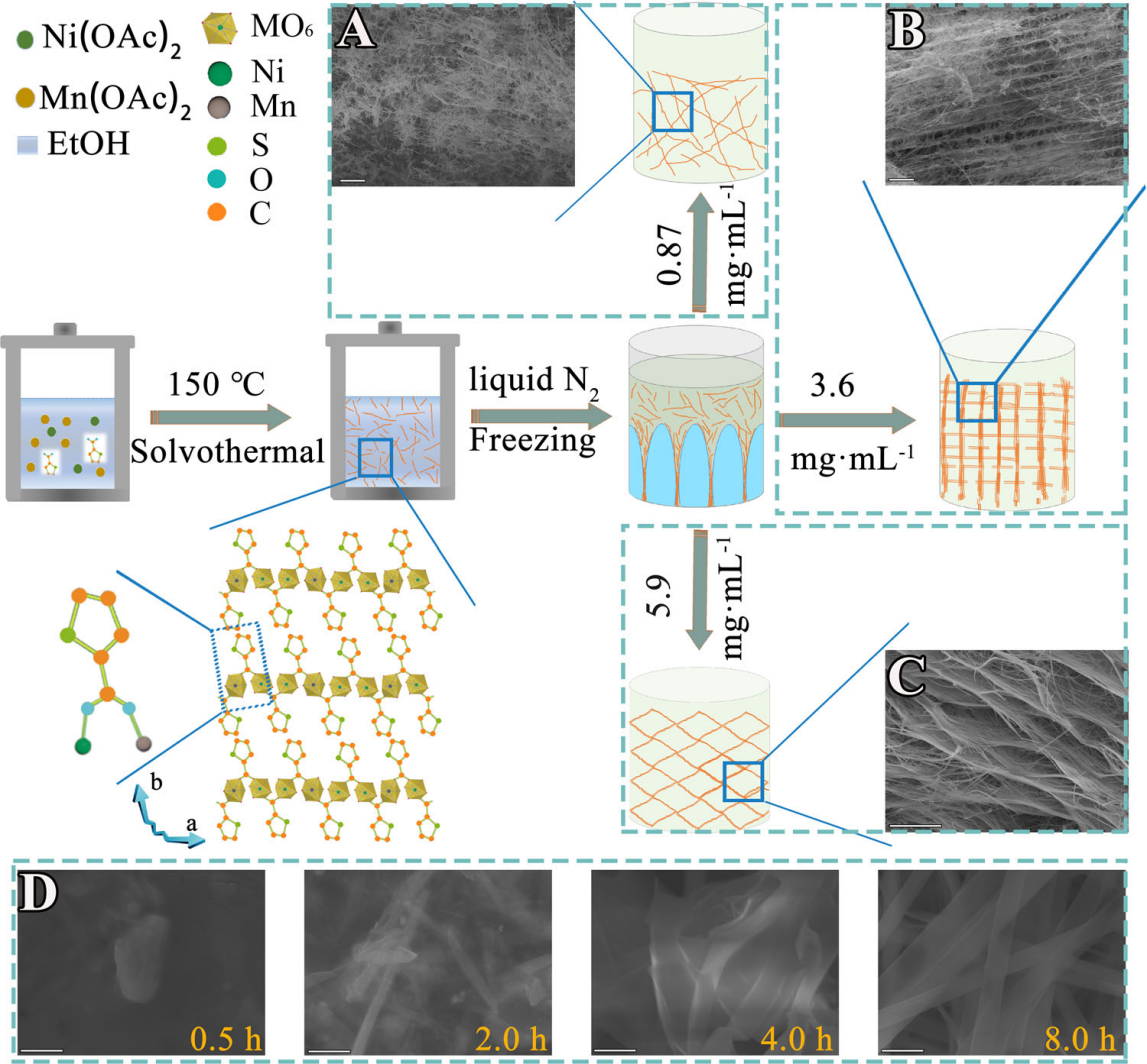
Synthesis of nickel, manganese–metal–organic framework aerogels. (A–C) SEM images and scheme of NiMn‐MOF synthesis from aqueous dispersion with concentration of (A) 0.87 mg·mL^−1^, (B) 3.6 mg·mL^−1^, and (C) 5.9 mg·mL^−1^ (scale bars for panels A, B, and C are 30, 30, and 20 µm, respectively); (D) SEM images of reaction intermediates collected at different reaction durations during NiMn‐MOF synthesis (scale bar: 1 µm)

As a proof‐of‐concept experiment, NiMn‐MOF aerogels are prepared by a two‐step procedure (Figure 1A–C). The first step of producing NiMn‐MOF nanobelts, nickel, and manganese acetate salts are mixed with an organic ligand of 2‐thiophenecarboxylic acid in an ethanol solution, which is then subjected to hydrothermal treatment at 150 °C. By tuning hydrothermal durations, we could examine reaction intermediates. According to scanning electron microscopy (SEM) and elemental mapping images, irregular nanoparticles without apparent elemental segregation are observed in the NiMn‐MOF intermediate obtained at 30 min, indicating that metal ions and ligands begin to nucleate within this period (Figure 1D and Figures S1A,B and S2A–D). Mixtures of irregular nanoparticles and nanobelts are then spotted in intermediates collected at 1–4 h, indicating that the crystal growth process occurs (Figure 1D and Figures S1C–H and S2E–L). After which, the length of MOF nanobelts increases during 6 to 8 h because of continuous crystal growth, and it ends after 10 h according to neglectable morphology change after then (Figure 1D and Figures S1I–N and S2M–P).

**FIGURE 2:**
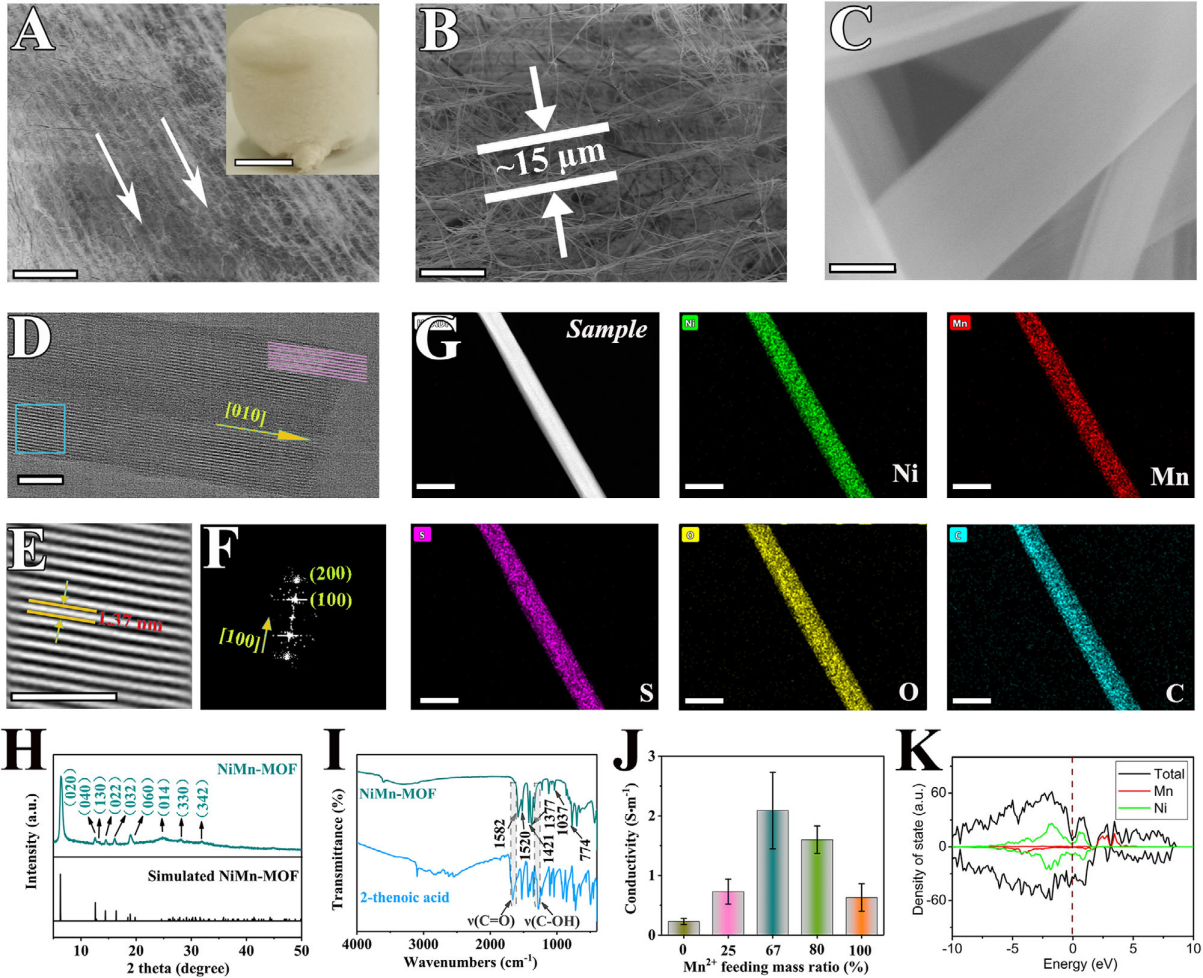
Morphological and structural characterizations of nickel, manganese–metal–organic framework aerogels. (A–C) SEM images (scale bars for panels A, B, and C are 100 µm, 20 µm, and 200 nm), inset of (A) is an optical image (scale bar: 1 cm); (D,E) TEM images (scale bars in panels D and E are 20 and 5 nm, respectively); (F) FFT image of panel D; (G) TEM element mappings of Ni, Mn, S, O, and C (scale bar: 500 nm); (H) Experimental and simulated XRD patterns; (I) FT‐IR spectra; (J) Electrical conductivity of NiMn‐MOF synthesized from different manganese feeding percentages; (K) Density of states

According to literatures^[^ ^17^, ^18^ ^]^ and X‐ray diffraction (XRD) patterns (Figure 2H and Figures S3 and S4), as‐formed NiMn‐MOF nanobelts consisted of alternating organic units (2‐thiophenecarboxylic acid group) and inorganic units (NiO_6_ or MnO_6_), where a carboxyl group on each ligand bridges two transition metal ions, and each ion coordinates with two opposite carboxyl groups and four equatorial ethanol molecules (Figure 1). Other MOF nanobelts, including Ni‐MOF, Mn‐MOF, Co‐MOF, NiCo‐MOF, CoMn‐MOF, NiCoMn‐MOF, and Ni‐MIL‐77‐MOF, are also synthesized by a similar method except varying the metal sources (Figures S3–S10) or organic ligands (2‐thiophenecarboxylic acid or glutaric acid, Figures S11 and S12).

The second step for assembling these MOF nanobelts into 3D macroscopic aerogels, NiMn‐MOF nanobelts are dispersed in aqueous solutions with different mass concentrations and later freeze‐dried (inset of Figure 2A). Both Fourier transform infrared spectroscopy (FT‐IR, Figure 2I and Figures S13 and S14) and Zeta‐potential analysis (Figure S15) were used to verify the presence of many hydroxyls and carboxyl functional groups in NiMn‐MOF, this reassures its good dispersibility in water (Figure S16). The MOF nanobelt dispersion was then frozen using liquid nitrogen, during which nanobelts were forced to align in the direction of how ice naturally forms, resulting in a highly ordered 3D structure.^[^ ^19^, ^20^ ^]^ During this process, the resultant microstructure is affected by a number of factors, such as dispersion concentrations, freezing speeds, and drying methods.

Firstly, we adjust the dispersion concentration of NiMn‐MOF nanobelts from 0.2 to 8.3 mg·mL^−1^. The aerogel architecture obtained at a low concentration (<1.3 mg·mL^−1^) is randomly oriented with the cross‐linked fluffy network, which does not show any superplastic property during compression‐unloading testing (Figure 1A). When the concentration of NiMn‐MOF dispersion reaches 3.6 mg·mL^−1^ and above (such as 5.9 mg·mL^−1^), the 1D nanobelts assemble into ordered two‐dimensional (2D) sheets, further to honeycomb‐like 3D aerogels (Figure 1C,D). In addition, the drying method is also important, as we can only obtain powder‐like samples by drying as‐prepared NiMn‐MOF nanobelts in an oven at 60 °C (Figure S17). Further, the freezing speed (rapid freezing: full‐scale liquid nitrogen pouring and slow freezing: bottom‐up pouring) should also be manipulated with care. Rapid freezing (freezing rate: 50 °C·min^−1^) can only generate disordered cross‐linked aerogels (Figure S18C,D) with poor mechanical properties (Figure S18A,B). While with slow freezing (5 °C·min^−1^), water condensation in vertical direction would force 1D NiMn‐MOF nanobelts to assemble into 2D sheets along the leading edge of ice movement. Finally, one 2D nanosheet laterally connects with each other into a highly ordered 3D compressible MOF aerogel after thawing (Figures 1 and 2A–C). This procedure was applied to fabricate other flexible 3D MOF aerogels composed of single (Ni‐, Co‐, Mn‐), binary (NiCo‐, CoMn‐) and ternary (NiCoMn‐) metal sites (Figures S5–S10) or organic ligands (glutaric acid, Ni‐MIL‐77, Figures S11 and S12).

### 2.2 Morphology and structure characterizations of superplastic NiMn‐MOF aerogels

The as‐prepared NiMn‐MOF is present as a solid yellow‐gray cylinder with sizes of a few centimeters and mass density of 3.6 mg·cm^−3^ (inset of Figure 2A). The macroscopic aerogel is made of ordered 2D sheets with adjacent spacing of approximately 15 µm (Figure 2A,B), which are composed of flexible 1D nanobelts with dimensions of 400 nm in width and tens of micrometers in length (Figure 2C). The high‐resolution transmission electron microscope image displays good crystallinity of NiMn‐MOF with well‐defined lattice viewed along the [010] axis (Figure 2D), and a lattice spacing of 1.37 nm of (200) plane (Figure 2E). Fast Fourier transform image (FFT, Figure 2F) indicates a well‐developed single crystal feature with (100) and (200) planes along the [100] direction. Further, TEM element mapping images exhibit homogeneously distribution of Ni, Mn, S, O, and C elements throughout nanobelts (Figure 2G).

The crystal structure of NiMn‐MOF characterized by XRD shows a series of diffraction peaks, matching well with simulated patterns (Figure 2H). The strongest diffraction peak at 2*θ* = 6.4^°^ is ascribed to (200) plane of NiMn‐MOF, which displays a lattice spacing of 1.37 nm (Figure 2E). Other MOFs also show XRD patterns similar to simulated results (Figures S3 and S4). Furthermore, X‐ray photoemission spectroscopy (XPS) indicates an atomic ratio of 61.1% for Mn/Ni (Figure S19). Consequently, combination of element analysis (EA, Figures S5–S10 and S20), inductively coupled plasma optical emission spectroscopy (ICP‐OES, Table S1), thermogravimetric analysis (TGA, Figure S21), FT‐IR (Figure 2I and Figures S13 and S14) and XPS suggest the chemical formula of NiMn‐MOF is Ni_0.62_Mn_0.38_(C_5_H_3_SO_2_)(C_2_H_6_O)_2_.

Traditionally, MOFs are considered as poor electrical conductors (usually with conductivity of 10^−10 ^S·m^−1^) that greatly limits their applications in fields of electrical and electrochemical devices.^[^ ^21^, ^22^ ^]^ While, as‐prepared NiMn‐MOF (67% of Mn) shows an excellent conductivity of 2.09 ± 0.64 S·m^−1^, the highest compared to other samples, including Ni‐MOF (0.23 ± 0.05 S·m^−1^), NiMn‐MOF (25%, 0.73 ± 0.21 S·m^−1^), NiMn‐MOF (80%, 1.60 ± 0.23 S·m^−1^), and Mn‐MOF (0.63 ± 0.23 S·m^−1^, Figure 2J and Table S2). This result is consistent with simulated density of states (DOS) of NiMn‐MOF by density functional theory (DFT, Figure 2K), for Ni, Mn, and overall atoms across Fermi level.^[^ ^23^ ^]^ The above results unambiguously confirm that as‐synthesized NiMn‐MOF aerogel displays excellent electrical conductivity, which could be a potential electrocatalyst for HER from seawater.

### 2.3 Mechanical property of 3D MOF aerogels

**FIGURE 3:**
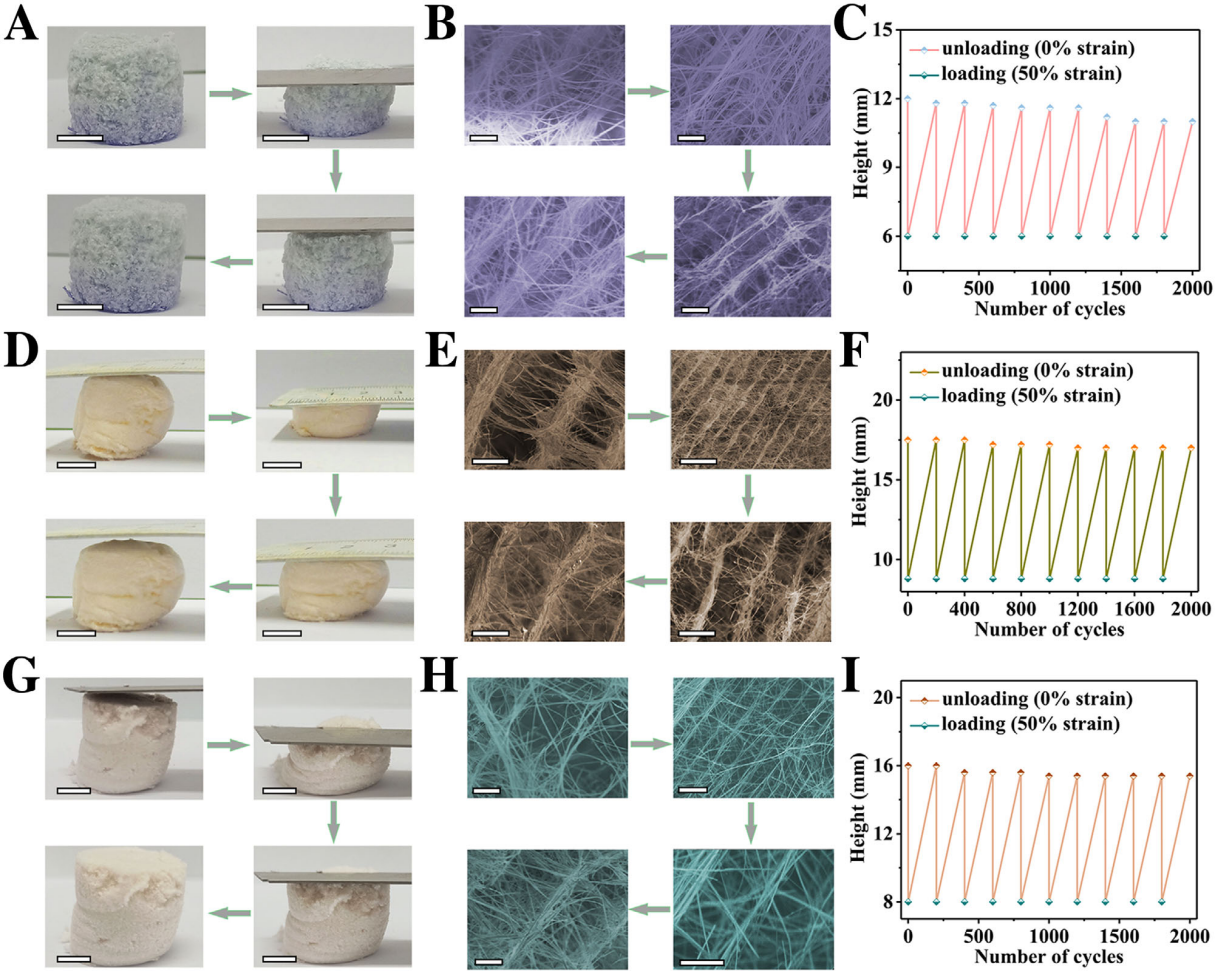
Mechanical property demonstration of metal–organic framework aerogels. (A–C) Co‐MOF aerogels; (D–F) NiMn‐MOF aerogels; (G–I) NiCoMn‐MOF aerogels; (A,D,G) Optical images (scale bar: 1 cm); (B,E,H) SEM images (scale bar: 10 µm); (C,F,I) Compression‐unloading performance

Compression‐resilience cycle testing for as‐synthesized MOF aerogels has been conducted, including Co‐ (single), NiMn‐ (binary), and NiCoMn‐ (ternary) MOFs with 2‐thiophenecarboxylic acid as the ligand, and Ni‐MIL‐77 MOF with glutaric acid as the ligand (Figure 3 and Figure S22). Applying 50% strain onto above 3D MOF aerogels, they could restore its original shape within 1 s once applied force released (Figure 3A,D,G and Figure S22A). Originally, the anisotropic layered structure of 3D Co‐MOF, NiMn‐MOF, NiCoMn‐MOF and Ni‐MIL‐77 MOF aerogels is observed (Figures 3B,E,H and Figure S22B), with layer spacing of 22, 95, 25, and 15 µm, respectively. Under 50% strain, it changes to 6, 20, 6, and 5 µm, while restore to 21, 90, 23, and 14.5 µm within 1 s once strain released, respectively. The compression‐resilience cycle is repeated 2000 times, after which only negligible shape deformation (8.3% for Co‐MOF, 2.9% for NiMn‐MOF, and 3.8% for NiCoMn‐MOF), was observed (Figure 3C,F,I). The Ni‐MIL‐77 aerogel also displays good mechanical properties during 10‐cycle testing (Figure S22C). The results suggest excellent superplasticity of as‐prepared 3D MOF aerogels, which have been synthesized using a ubiquitous strategy.

### 2.4 Electrochemical hydrogen evolution

Potential applications of as‐obtained superplastic MOF aerogels as a new class of flexible electrodes have been explored, such as HER from seawater electrolysis. As we know, flexible energy systems have attracted considerable interests because of remarkable properties such as small‐size units, lightweight, and shape conformability, which are promising in portable, foldable, and wearable devices.^[^ ^24^, ^25^, ^26^, ^27^ ^]^ However, HER systems are generally fabricated in forms of bulky and heavy architectures, which are far from flexibility. Conventional electrodes are fabricated by casting powder or thin‐film electrocatalysts, onto rigid substrates (like glassy carbon^[^ ^28^ ^]^ and FTO glass^[^ ^29^, ^30^ ^]^), resulting in bulk and fragile electrolytic devices. Flexible current collectors (such as carbon‐fiber paper^[^ ^24^ ^]^ and metal foams^[^ ^26^, ^27^ ^]^) have been employed to fabricate HER electrodes because of their high electrical conductivity, interconnected porous networks, and mechanical robustness. Integrating flexible substrates with active catalytic species has been achieved by post solution‐casting or direct growth. However, flexibility of as‐resultant electrodes is still compromised owning to the intrinsically fragile nature of catalyst powders or thin‐films, leading to considerable activity decay during electrode deformation.

**FIGURE 4:**
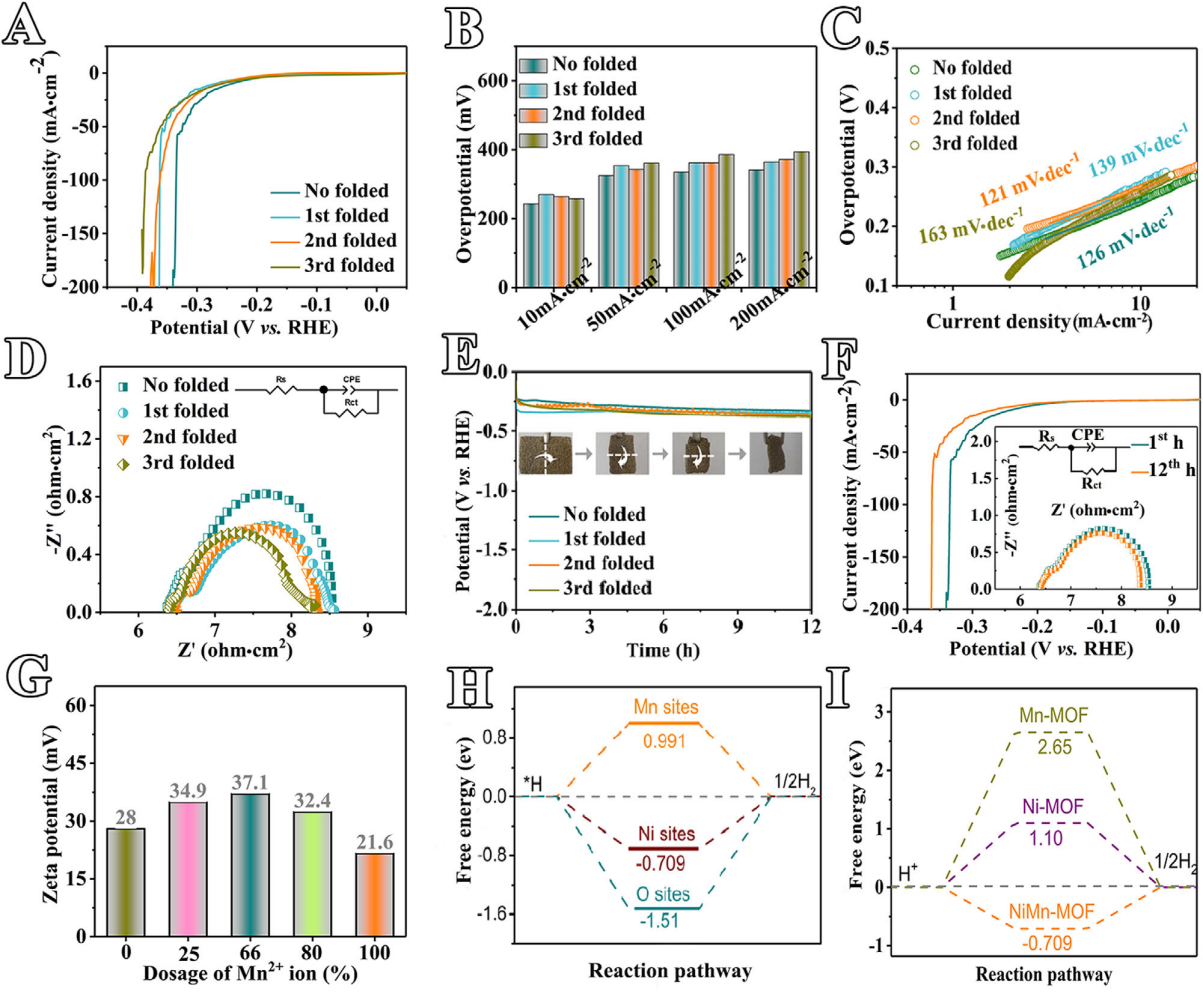
Electrocatalytic performance and mechanism study of HER from natural seawater electrolysis. (A) LSV curves of NiMn‐MOF electrode with different folding times; (B) Overpotentials to achieve different current densities; (C) Tafel plots; (D) EIS plots; (E) Stability testing for 12 h, inset of panel (E) shows optical images of catalyst electrodes with different folding times; (F) LSV curves of NiMn‐MOF before and after stability tests, inset of (F) is the EIS plot; (G) Zeta potentials in relation to feeding ratios (w/w) of metal salts (Ni and Mn) to synthesize NiMn‐MOF; (H) Gibbs energy diagrams of HER catalyzed by different active sites in NiMn‐MOF; (I) Gibbs energy diagrams of HER promoted by NiMn‐MOF, Ni‐MOF, and Mn‐MOF

Here we studied the performance of a superplastic MOF aerogel for HER from both simulated seawater (NaCl, Figures S23–S30 and Tables S5–S8) and natural seawater (collected from the Yellow Sea in Qingdao, China). Technically, a NiMn‐MOF electrocatalyst is obtained by adding nickel foam in the second step of freeze‐drying NiMn‐MOF dispersion.^[^ ^31^, ^32^ ^]^ Without deformation, the NiMn‐MOF electrode only requires an overpotential of ∼243 mV to achieve a current density of 10 mA·cm^−2^ in the natural seawater, which is similar with the simulated seawater (3 wt% NaCl, Figure 4A and Figure S23). Even under high current density of 200 mA·cm^−2^, the required HER overpotential of NiMn‐MOF is only ∼341 mV. Therefore, as‐prepared NiMn‐MOF is one of the most active electrocatalysts reported (Table S9). Notably, the HER activity of this superplastic electrode shows insignificant changes after structural deformation. The HER overpotential for NiMn‐MOF varies with electrode deformations. More specifically, the overpotentials with one‐, two‐, and threefold are 270, 264, and 258 mV for 10 mA·cm^−2^, 354, 343, and 361 mV for 50 mA·cm^−2^, and 362, 362, and 386 mV for 100 mA·cm^−2^, respectively. Impressively, NiMn‐MOF can achieve high current density of 200 mA·cm^−2^ at 364, 372, and 394 mV for one, two and three‐folds, respectively (Figure 4A,B and Table S7). While the fragile non‐superplastic bulk NiMn‐MOF displays rapid decay of HER activity with deformation (Figures S31 and S32), confirming the advantages of superplasticity in electrocatalyst fabrication.

Importantly, the electrocatalyst also displays facile reaction kinetics after deformation, as verified by similar Tafel slopes and charge‐transfer resistance (*R*_ct_) from electrochemical impedance spectroscopy (EIS) analysis (Figure 4C, Table S7). The Tafel slope only displays slight change, which is 126 (no fold) to 139 (onefold), 121 (twofold), and 163 mV·dec^−1^ (threefold), respectively. *R*_ct_ @−1.5 V *vs*. RHE of HER process promoted by NiMn‐MOF is 2.2 ohm·cm^2^ for no folds, 2.1 ohm·cm^2^ for onefold, 1.4 ohm·cm^2^ for twofold, and 1.9 ohm·cm^2^ for threefold (Figure 4D and Table S7).

As expected, the NiMn‐MOF has exhibited excellent electrochemical stability without or with deformation, as revealed by a chronopotentiometric test at 10 mA·cm^−2^ (Figure 4E and Figure S34), linear sweep voltammograms (LSVs), and EIS before and after testing (Figure 4F). The excellent electrochemical stability is further confirmed by other characterizations including SEM, elemental mappings, XRD, FT‐IR, and ICP mass spectrometer (ICP‐MS) (Figures S35, S36, and Table S10). All above results show that the NiMn‐MOF has excellent physical superplasticity, HER activity, and stability, and thus is a good electrocatalyst candidate for seawater splitting.

### 2.5 HER mechanism

To gain mechanism insights into HER promoted by NiMn‐MOF catalysts, DFT simulation has been conducted to calculate Gibbs free energy change of H* adsorption (Δ*G*_H*_), which has been considered as a key descriptor for HER activity.^[^ ^33^, ^34^, ^35^, ^36^ ^]^ Generally, an electrocatalyst with a positive Δ*G*_H*_ means low reaction kinetics of hydrogen adsorption, while a negative value suggests low reaction kinetics of hydrogen desorption, therefore the optimum value of Δ*G*_H*_ is zero. Firstly, Δ*G*_H*_ of different active sites (Ni, Mn, and O sites) in NiMn‐MOF with the most stable configuration, are calculated (Figure 4H and Figure S38). The Δ*G*_H*_ of Mn is quite positive (0.991 eV), indicating poor hydrogen adsorption for Mn sites, while that of O is very negative (−1.51 eV) suggesting poor hydrogen desorption for O sites. Δ*G*_H*_ of Ni active sites shows a value of −0.709 eV closer to zero, suggesting the active site for HER is the Ni atom (Figure 4H). Further, the Δ*G*_H*_ of as‐prepared MOF catalysts are compared, that NiMn‐MOF shows a much smaller Δ*G*_H*_ of −0.709 eV than Ni‐MOF (1.10 eV) and Mn‐MOF (2.65 eV) (Figure 4I and Figure S39). This result might be related to the configuration change of NiMn‐MOF that Ni‐H bond decreases by 0.01 angstrom as comparison to bare Ni‐MOF without Mn doping. In addition, the competitive co‐ordination between metals (Ni or Mn) with different electronegativity and oxygen (O) forms an asymmetric M‐O covalent bond, which may improve the exposure of cationic active sites, might also be a contribution to the enhancement of HER activity.^[^ ^37^ ^]^ According to Figure S24, the NiMn‐MOF even outperforms Pt/C in seawater electrolysis, which might be because the Pt active site is poisoned by adsorbed Cl‐ions, making hydrogen adsorption‐desorption sluggish.

The significant HER performance of NiMn‐MOF aerogel also benefits from its unique structure features. Firstly, the surface area of NiMn‐MOF has been examined by N_2_ isotherms^[^ ^38^, ^39^ ^]^ that Brunauer–Emmett–Teller specific surface area is 1.6 times larger than its powder counterpart (11.8 *vs*. 4.6 m^2^·g^−1^, Figure S40 and Table S4). This result is consistent with electrochemical double‐layer capacitance (*C*_dl_) testing (Figures S41, S42, and Table S5), that *C*_dl_ of NiMn‐MOF is 11.25 mF·cm^−2^ and roughly 3 times its powder counterpart (3.92 mF·cm^−2^). Comparing polarization curves normalized by electrochemical surface area (Figure S43), NiMn‐MOF still exhibits a smaller overpotential to achieve the same current density as comparison with Ni‐ and Mn‐MOFs, indicating its intrinsically high catalytic activity for HER. This result is consistent with Zeta potential, XRD, and FT‐IR results (Figures 2H,I and 4G), which proves the bimetallic synergy effect to improve HER. Secondly, the NiMn‐MOF displays excellent electrical conductivity both theoretically and experimentally (Figure 2J,K and Table S2), which differs from traditional MOF materials. The conductive nature can ensure fast charge transport within electrodes that lead to excellent catalytic activity, which is related to the strong coordination between metal ions and organic ligands.

Thirdly, from the perspective of microstructure, the superplastic NiMn‐MOF aerogel exhibits a hierarchical porous structure made of 1D nanobelts assembled into 2D nanosheets, and further to 3D macroscopic architecture. During stress‐strain testing, the mutual repulsion between organic groups (─OH and ─COOH from 2‐thiophenecarboxylic acid) on the surface of nanobelts compromises external forces, which are easily recover after releasing any load. The hierarchically porous structure of NiMn‐MOF aerogels can also provide enormous voids between adjacent nanobelts that can prevent strain/stress during vigorous gas evolution from electrochemical testing. Because of such properties, this material guarantees excellent stability during electrocatalytic reactions involving gaseous products (O_2_, N_2_, H_2_, etc.).

## 3 CONCLUSIONS

In conclusion, 3D superplastic MOF aerogels have been synthesized by mimicking the hierarchical architecture of nature cork via a low‐cost, two‐step solution procedure. This material shows attractive properties, such as superplasticity, high electrical conductivity, large specific surface area, and rich active sites. As a result, the 3D MOF exhibits excellent HER activity and stability in natural seawater electrolysis. Such exceptional materials will pave the way for a broad range of technological applications. Particularly, our work allows exploring applications of MOFs in a self‐supporting, structurally flexible, and 3D macroscopic form. Furthermore, a wide range of functional materials could be readily introduced into the hierarchical porous structure of MOF aerogels, offering opportunities to develop novel MOF‐based nanomaterials for applications such as photocatalysis, electrocatalysis, metal–air batteries, biosensors, and so on.

## CONFLICT OF INTEREST

Jingjing Duan is a member of the *Exploration* editorial board. The authors declare that they have no competing interests.

## Supporting information

Supporting information
Detailed data of material characterizations, electrochemical testing, and other supporting data are included.
