# Supplementary material for: Biomimetic assembly to superplastic metal–organic framework aerogels for hydrogen evolution from seawater electrolysis
Source: Exploration (Beijing). 2021 Sep 23;1(2):20210021. doi: 10.1002/EXP.20210021 (PMC10190981; doi:10.1002/EXP.20210021)
Supplement: Supplementary file 1 — Supporting information Detailed data of material characterizations, electrochemical testing, and other supporting data are included. [file EXP2-1-20210021-s001.docx]

**Supplementary Information**

**Biomimetic assembly of superplastic metal-organic framework aerogels for hydrogen evolution from seawater**

*Yuntong Sun,^1^ Shuaishuai Xu, ^1^ César A Ortíz‐Ledón, ^2^ Junwu Zhu,* ^1^ Sheng Chen,* ^1^ Jingjing Duan*^1^*

*^1^*Key Laboratory for Soft Chemistry and Functional Materials, School of Chemical Engineering, School of Energy and Power Engineering, Nanjing University of Science and Technology, Nanjing, Jiangsu 210094, China

*^2^*Department of Chemistry, University of Wisconsin-Madison, Madison, WI 53706, USA

To whom correspondence should be addressed: zhujw@njust.edu.cn; [sheng.chen@njust.edu.cn](mailto:sheng.chen@njust.edu.cn); jingjing.duan@njust.edu.cn

**Table of Contents**

1. Chemicals.......................................................................................................................................S4

2. Material synthesis...........................................................................................................................S4

3. Physical characterizations..............................................................................................................S5

4. Electrochemical characterizations..................................................................................................S6

5. Theoretical simulations..................................................................................................................S7

6. Figures and Tables..........................................................................................................................S9

7. References....................................................................................................................................S62

**Supporting Figure Contents**

**Figures S1 to S2.** Morphological characterizations of NiMn-MOF nanobelts at different reaction times.

**Figures S3 to S4.** Experimental and simulated XRD patterns.

**Figures S5 to S10.** Morphological characterizations of Ni-MOF, Co-MOF, Mn-MOF, NiCo-MOF, CoMn-MOF and NiCoMn-MOF aerogels.

**Figures S11 to S12.** Morphological characterization, XRD pattern and FT-IR spectra of Ni-MIL-77 aerogels with glutaric acid as organic ligand.

**Figures S13 to S14.** FT-IR spectra.

**Figures** **S15 to S16.** Optical photos and Zeta potentials of NiMn-MOF nanobelts after standing for different time.

**Figures S17 to S18.** The morphological characteristics of NiMn-MOF obtained by directly drying the nanobelts in the air at 60℃ and rapidly frozen in liquid nitrogen.

**Figures S19 to S20.** XPS survey spectrums and EDS elemental mapping images of NiMn-MOF.

**Figures S21.** TGA profiles.

**Figure S22.** Mechanical properties of Ni-MIL-77 aerogels.

**Figures S24 to S31.** Electrochemical test of NiMn-MOF and other electrodes in NaCl electrolyte.

**Figures S32 to S34.** Electrochemical test of NiMn-MOF in natural seawater.

**Figures S35 to S37.** Structure characterizations of NiMn-MOF before and after electrochemical test.

**Figures** **S38 to S39.** Optimized lattice structure for NiMn-MOF、Ni-MOF and Mn-MOF absorbed hydrogen atoms at different active sites.

**Figure S40.** N_2_ adsorption-desorption isotherms of the NiMn-MOF and NiMn-MOF powder.

**Figures** **S41 to S43.** ECSA curves of NiMn-MOF and other electrodes.

**Table S1**. ICP-OES analysis of NiMn-MOF and other comparison samples.

**Table S2.** Electrical conductivity of NiMn-MOF and other comparison samples.

**Table** **S3.** Average mass loadings of NiMn-MOF catalysts on nickel foam substrates.

**Table** **S4.** Brunauer-Emmett-Teller (BET) surface area of NiMn-MOF and its powder counterpart.

**Table S5.** Comparison of the HER activities for NiMn-MOF with other electrocatalysts in 3 wt% NaCl solution.

**Table** **S6.** Comparison of the HER activities for NiMn-MOF in different concentrations of NaCl electrolyte.

**Table** **S7.** Comparison of the HER activities for NiMn-MOF with different folding times in natural seawater.

**Table S8.** Comparison of the HER activities for bulk NiMn-MOF with different folding times in natural seawater.

**Table** **S9.** Comparison of the HER activities for NiMn-MOF with recently reported electrocatalysts in neutral electrolytes.

**Table** **S10.** The concentration for Ni and Mn in the electrolyte in the presence of NiMn-MOF after stability testing and without applied HER overpotential.

**1. Chemicals.** Nickel foam (NF, 2.8 cm×2 cm×1.6 mm, bulk density 350 g m^-2^, porosity > 95%), 2-thiophenecarboxylic acid (2-C_5_H_3_O_2_S, 99%), nickel acetate tetrahydrate (Ni(Ac)_2_·4H_2_O, 99%), Manganese (II) acetate tetrahydrate (Mn(Ac)_2_·4H_2_O, 98%), Cobalt (II) acetate tetrahydrate (Co(Ac)_2_·4H_2_O, 99%), ethanol (EtOH, absolute) and sodium chloride (NaCl, 99.8%) were purchased from Sigma-Aldrich and directly used without further treatment or purification. All aqueous solution was prepared with high-purity de-ionized water (DI-water, resistance 18.2 MΩ cm^-1^ ). Natural seawater was collected from the Yellow Sea (Qingdao, China), which was filtered to remove visible impurities before usage.

**2. Material synthesis**

**Synthesis of NiMn-MOF aerogels.** In the first step, 33 mg of Ni(Ac)_2_·4H_2_O and 66 mg Mn(Ac)_2_·4H_2_O were added to 30 mL of EtOH; Next, 100 mg of organic ligand (2-C_5_H_3_O_2_S) was added into this solution under magnetically stirring for 1 hour (hr), followed by hydrothermal treatment at 150 °C for 12 hrs. After cooling down to room temperature, the as-produced NiMn-MOF was taken out and washed three times with EtOH and DI-water, repeatedly. In the second step, NiMn-MOF was mixed with 20 mL of DI-water to form a dispersion, which was slowly frozen-dried with liquid nitrogen. The electrodes were prepared by adding NF into above solution followed by a similar procedure.

**Synthesis of other MOF aerogels.** A number of other MOF aerogels have been synthesized using a similar procedure with NiMn-MOF only differing in metal precursors, for example, Ni-MOF (only Ni source), Mn-MOF (only Mn source), Co-MOF (only Co source), NiMn-MOF (25, 60, and 80 wt% of Mn/Ni), NiCo-MOF (1:1 for Ni : Co in mass ratio), CoMn-MOF (1:1 for Co : Mn in mass ratio), NiCoMn-MOF (1:1:1 for Ni : Co : Mn in mass ratios).

**Synthesis of** **NiMn-MOF powders.** The synthetic procedure is similar with that of NiMn-MOF by replacing freeze-drying with 60 °C drying in an oven. To fabricate catalyst electrodes, NiMn-MOF powder was dispersed in isoproponal/water (v/v = 1/3) mixed solvents with 1 wt% Nafion as the binder, and then being drop casted onto a NF substrate with mass loading of 0.48 mg cm^-2^.

**Calcination of NiMn-MOF at high temperature.** For reliable comparison, NiMn-MOF was calcined at 600 °C for 3 hrs with an elevated rate of 2 °C min^-1^ in Ar atmospheres.

**Synthesis of Ni-MIL-77 aerogel** **by using** **glutaric acid as the organic ligand.** In the first step, 988 mg of Ni(Ac)_2_·4H_2_O, 792 mg of HOOC(CH_2_)_3_COOH and 460 mg KOH were added to 40 mL of mixture solution containing H_2_O and EtOH (*V*_H2O_: *V*_EtOH_ = 1 : 1). Next, 4 mL of NaOH (0.4 M) was added into the above solution while magnetically stirring, followed by hydrothermal treatment at 180 °C for 48 hrs. The as-produced Ni-MOF nanobelts were washed 3 times with EtOH and DI-water. In the second step, the aerogel was prepared by using a freeze-drying method similar with that of NiMn-MOF.

**3. Physical characterization**

Field emission scanning electron microscope was carried out on JEOL (FESEM 7800F Prime); transmission electron microscope (TEM) and high resolution transmission electron microscope (HRTEM) were conducted on an aberration-corrected TEM (FEI Titan 80-300, 300 KV acceleration voltage); energy-dispersive X-ray spectroscopy (EDS) and element mapping were acquired on the SEM (OXFORD X-Max^N^ 150 10 KV). Nitrogen adsorption-desorption isotherms were measured on a Micromeritics ASAP 2020 Plus analyzer at 77 K with the corresponding pore size distributions estimated using the Barrett-Joyner-Halenda (BJH) model. X-ray diffractometer (XRD) was examined on a Smartlab machine (9 kW, 40 kV, 40 mA, λ=1.5418 Å) with Cu-Kα radiation; X-ray photoelectron spectroscopy (XPS) was collected between 0 and 1400 eV on an Axis Ultra (Thermo ESCALAB 250XI) XPS spectrometer equipped with an Al Kα source (1486.6 eV photon energy). Fourier transform infrared (FT-IR) spectra were recorded in a Thermofisher NICOLETIS10 FT-IR spectrometer; electrical conductivity were tested on a Signatone four point probing system.

Conductivity has been measured by a Signatone four-probe probing system on a catalyst pellet.[^1^](#_ENREF_1) In detail, pellets for conductivity measurements were prepared by cold isostatic pressing using an infrared tablet press mold. The conductivity (*σ*) was calculated according to the equation:

*σ* = (*I*/*V*) × (*l*/*A*)

Where *I* is current, *V* is voltage and *A* is cross-sectional area of the conductor (*A* = *a* × *d*). Parameter *l*, *a* and *d* is voltage probe’s distance, sample width and thickness, respectively.

**4. Electrochemical characterization**

Electrochemical tests were carried out on a CHI 760E electrochemical workstation in a three-electrode system using as-synthesized catalyst electrodes, graphite rods and Hg/Hg_2_Cl_2_ electrode (KCl saturated) as working electrode, counter electrode and reference electrode, respectively. The catalytic electrode was produced by interweaving NiMn-MOF with NF, which was achieved by introducing NF in the second step of freeze-drying of NiMn-MOF.[[^2,^[^3^](#_ENREF_3)](#_ENREF_2)](#_ENREF_2) The electrolyte is the home-made NaCl solution with different mass concentrations: 0.5 wt%, 1.5 wt%, 3 wt%, 6 wt%, and 10 wt%. Research is also conducted in natural seawater collected from the Yellow Sea (Qingdao, China).

Linear sweep voltammogram (LSV) plots were recorded at the scan rate of 5 mV s^-1^ with 85% *iR*-compensation and without *iR*-compensation. HER potential (V) *vs.* E_Hg/Hg2Cl2_ has been converted to reversible hydrogen electrode potential using the following equation:

E_RHE_ = E _Hg/__Hg2Cl2_ + 0.059×pH + 0.242.

The Tafel slope was calculated according to the Tafel equation as follows:

*η* = *b* log *j* + *a*,

where *η* is overpotential (V, calculated by |E-E* HER|), *j* is current density (mA cm^-2^), and *b* is Tafel slope (mV dec^-1^).

Long-term durability was tested by chronoamperometric response at a current density of -10 mA cm^-2^ for 12 hrs, with corresponding LSVs before and after testing collected for comparison. Electric double layer capacitance (C_dl_, mF cm^-2^) of the working electrode was obtained from the double-layer charge-discharge diagram using CVs in a small potential range of 0.027~0.127 V (*vs.* RHE). Electrochemical impedance spectroscopy (EIS) was measured at -1.5 V (*vs.* Hg/Hg_2_Cl_2_) from 100 KHz to 0.01 Hz.

Electrochemical surface area (ECSA) of NiMn-MOF was calculated using the experimental double-layer capacitance (C_dl_), which was determined by using CVs at various scan rates (10-20 mV s^-1^) in a small potential range of 0.027~0.127 V (*vs.* RHE). Both a plot of the *∆J*= (*J_a_* - *J_c_*) (mA) at 0.077 V (*vs.*RHE) against scan rate are nearly linear and the double layer capacitance (*C_dl_* (mF)) is obtained by its slopes. Based on the reported method,[^4^](#_ENREF_4) we use benchmark capacitance of *C_s_* = 0.035 mF cm^-2^ (typical reported value) to calculate ECSA of various electrodes (ECSA=*C_dl_* / (*C_s_* per cm^-2^)).

**5. Theoretical simulation**

Computational work was conducted by using density functional theory (DFT) with spin polarization. The ionic cores were illustrated by the projector-augmented wave (PAW) method. The Perdew-Burke-Ernzerhof (PBE)[^5-7^](#_ENREF_5) functional in combination with DFT + U approach was used for electron exchange-correlation within the generalized gradient approximation (GGA) implemented in VASP package code.[[[[[[[^8,^[^9^](#_ENREF_9)](#_ENREF_8)](#_ENREF_8)](#_ENREF_5)](#_ENREF_4)](#_ENREF_4)](#_ENREF_4)](#_ENREF_4) An effective on-site Coulomb interaction parameter of U = 3 and 3 eV was selected for Ni and Mn according to previous reports.[^9^](#_ENREF_9) The cut-off energy for plane wave expansion was fixed at 400 eV, optimized from a range of cut-off energies. The electronic self-consistent-loop criterion was set to 10^-4^ eV. During geometry optimization, the structures were relaxed to forces on all atoms smaller than 0.05 eV/Å. A Gaussian smearing method was employed with 0.50 eV width. The K-points for structural optimization in all models were set to 5 × 3 × 1. For density of state (DOS) calculations, the k-points were set to be 10 × 6 × 1.

A surface was cleaved across *a* direction to form a NiMn-MOF slab. The slab is a sandwich structure with the top and bottom layers containing nickel and manganese atoms with the two layers linked by thiophene acid groups. According to the XPS and element mappings, one third of nickel was replaced by manganese. Further, test calculations were conducted on different doping sites, and the most stable structure was presented in Supplementary Fig. 38.

Further, different adsorption sites for H* were investigated for studying the mechanism of HER. During optimization of H* adsorption the bottom layer of substrate atoms was fixed to allow a time-efficient relaxation of the adsorbed species.

The detailed Gibbs free energy calculation for HER has been carried out as it follows:

*G* = *E* + *∫*C_P_ *dT* – *TS*

where *G*, *E* and *C_P_* refer to the chemical potential (partial molar Gibbs free energy), electronic energy and heat capacity, respectively. The entropy term can be expressed as the sum of the translational, rotational, vibrational and electronic contributions as to:

*S* = *S_t_* + *S_r_* + *S_v_* + *S_e_*

And finally, intrinsic zero-point energy (ZPE) and extrinsic dispersion (D) corrections can be included to finally obtain:

*G* = *E* + *∫*C_P_ *dT* – *T*(*S_t_* + *S_r_* + *S_v_* + *S_e_*) + ZPE + D

Since *S_e_* ≈ 0 at the fundamental electronic level.

For the case of solids and adsorbates, some approximations can be assumed:

1. As for gases, at the fundamental electronic level *S_e_* ≈ 0.

2. Translational and rotational motions can be neglected, therefore, *S_t_* ≈ 0 and *S_r_* ≈ 0. In this sense, all entropy contribution comes from vibrations: S = *S_v_*. Similarly, translational and rotational contributions to the heat capacity are neglected.

Therefore, Gibbs free energies for the different states have been calculated as to:

*G* = *E* + *∫*C_P_ *dT* – *TS_v_* + ZPE + D

**6. Figures and Table**


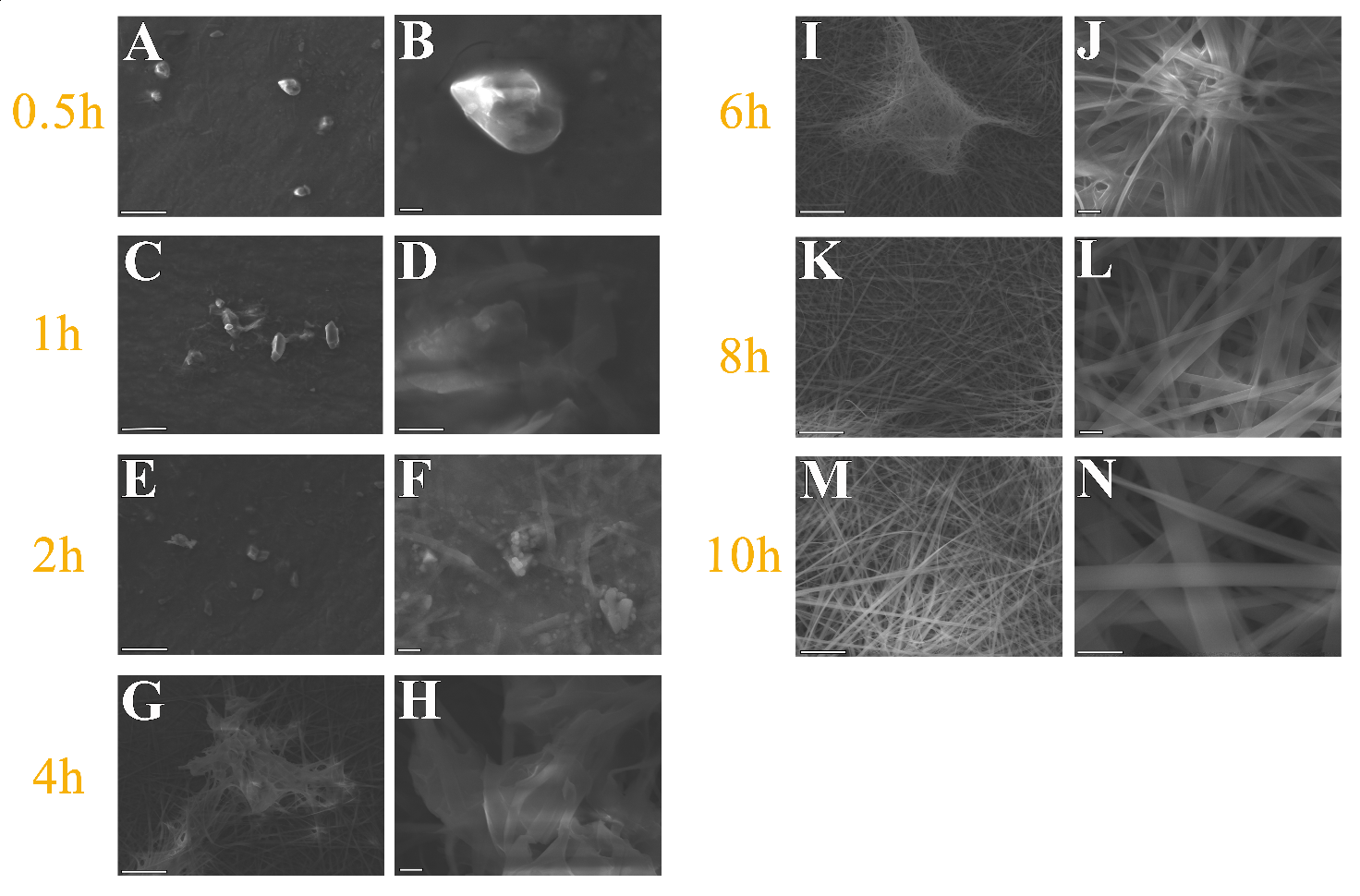


**Figure S1.** Morphological characterizations of reaction intermediates collected at different durations during synthesis of NiMn-MOF nanobelts (scale bars for A, C, E, G, I, K and M are 10 μm, B, D, F, H, J, L and N are 1 μm).


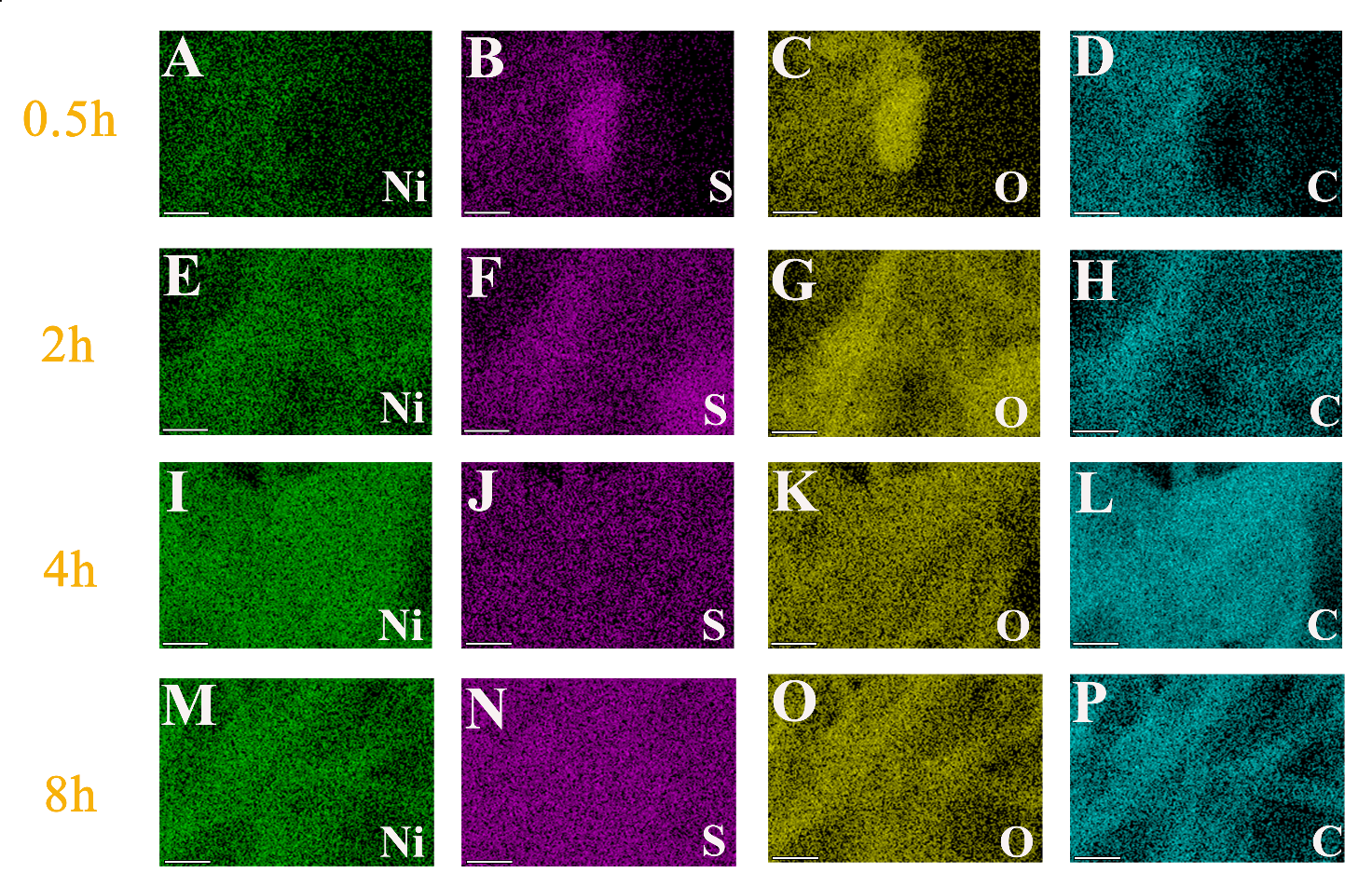


**Figure S2.** SEM elemental mappings of reaction intermediates obtained at different duration during NiMn-MOF nanobelt synthesis (scale bars, 1 μm).


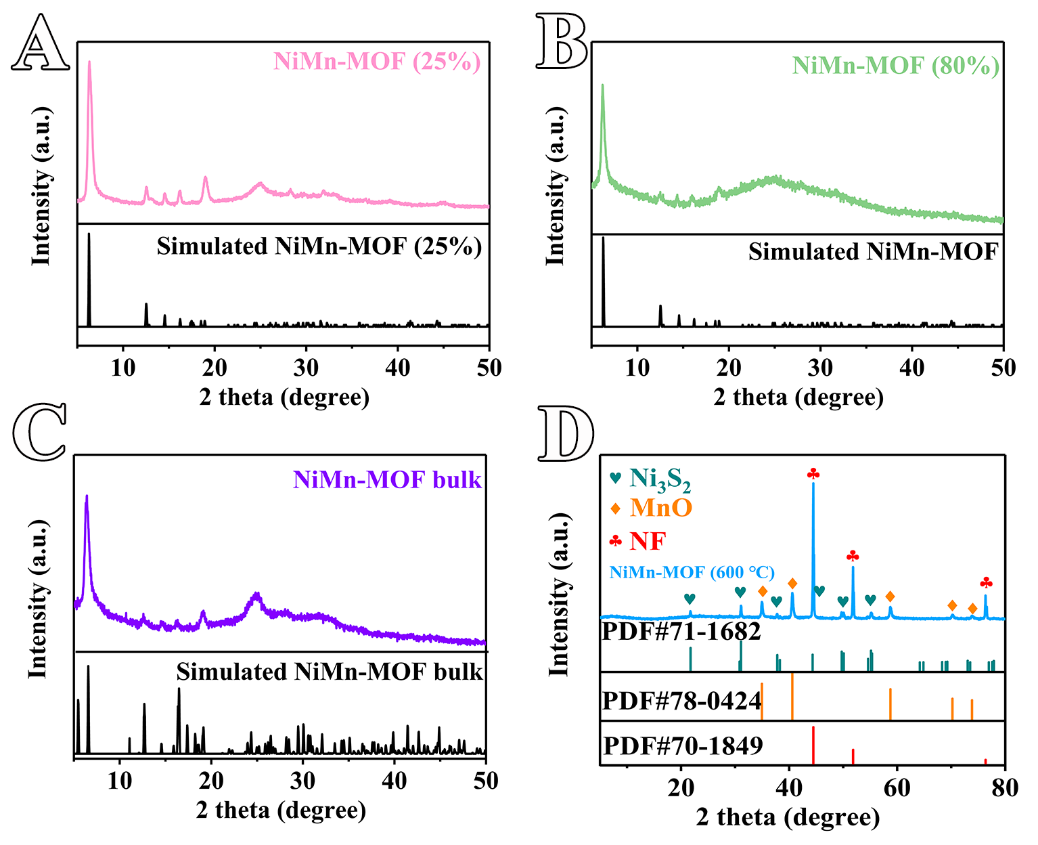


**Figure S3.** Experimental and simulated XRD patterns of (A) NiMn-MOF (25% of Mn), (B) NiMn-MOF (80% of Mn) and (C) bulk NiMn-MOF (powder); (D) NiMn-MOF treated at 600°C composed of Ni_3_S_2_, MnO, and nickel foam (NF) components.

**Supplementary note.**

XRD patterns showed that NiMn-MOF, NiMn-MOF bulk and other comparative samples (different Ni: Mn ratio) had similar diffraction characteristics, and the crystallinity decreased with the increase of Mn: Ni. The low-angle peaks (2θ=6.4^o^) are shown along the growth direction of the 1D nanobelts (Figure. 2G and Figure. S3,S4). The annealed NiMn-MOF was confirmed to be a mixture of pure Ni (PDF# 70-1849), Ni_3_S_2_ (PDF# 71-1682) and MnO (PDF# 78-0424, Figure. S3D), thus indicating the decomposition of MOF structure at high-temperature annealing.


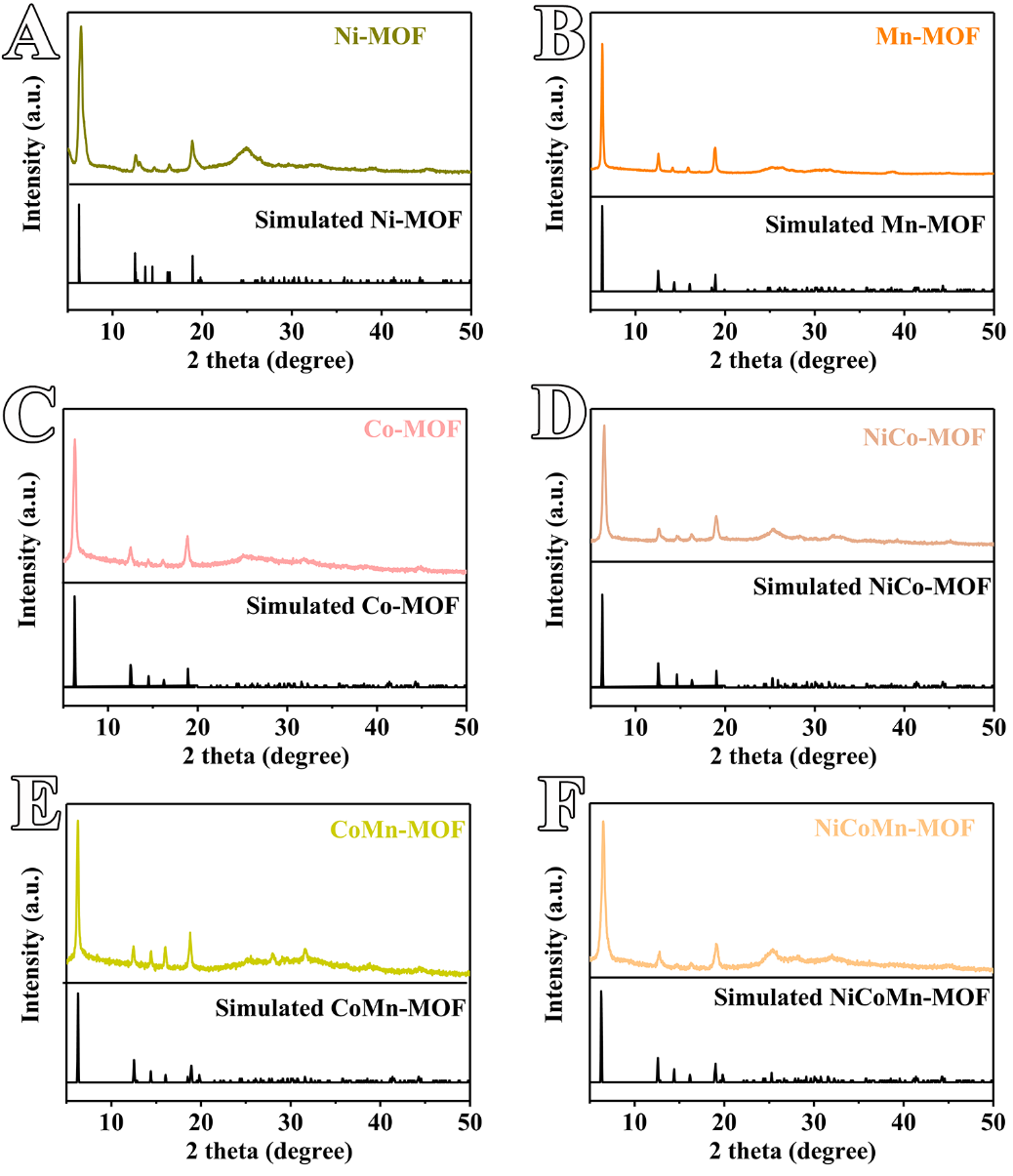


**Figure S4.** Experimental and simulated XRD patterns of (A) Ni-MOF, (B) Mn-MOF, (C) Co-MOF, (D) NiCo-MOF, (E) CoMn-MOF and (F) NiCoMn-MOF.


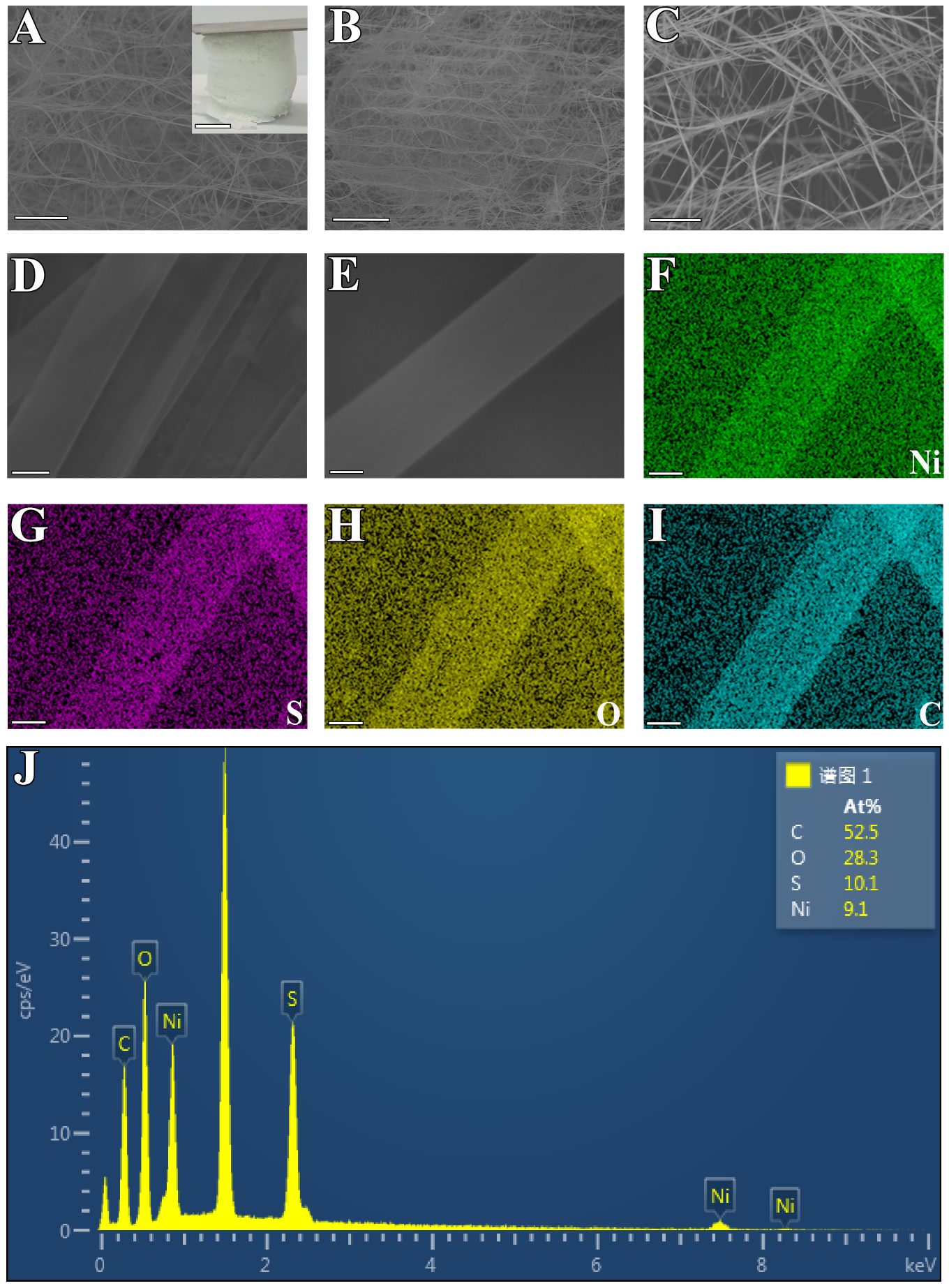


**Figure S5.** Morphological characterization of Ni-MOF aerogels. (A-E) SEM images (scale bars for A, B, C, D and E are 40 μm, 20 μm, 10 μm, 200 nm and 200 nm), inset of (A) is an optical image (scale bars: 1 cm); (F-I) SEM elemental mappings of Ni, S, O, C (scale bars: 200 nm); (J) EDS elemental mapping images.


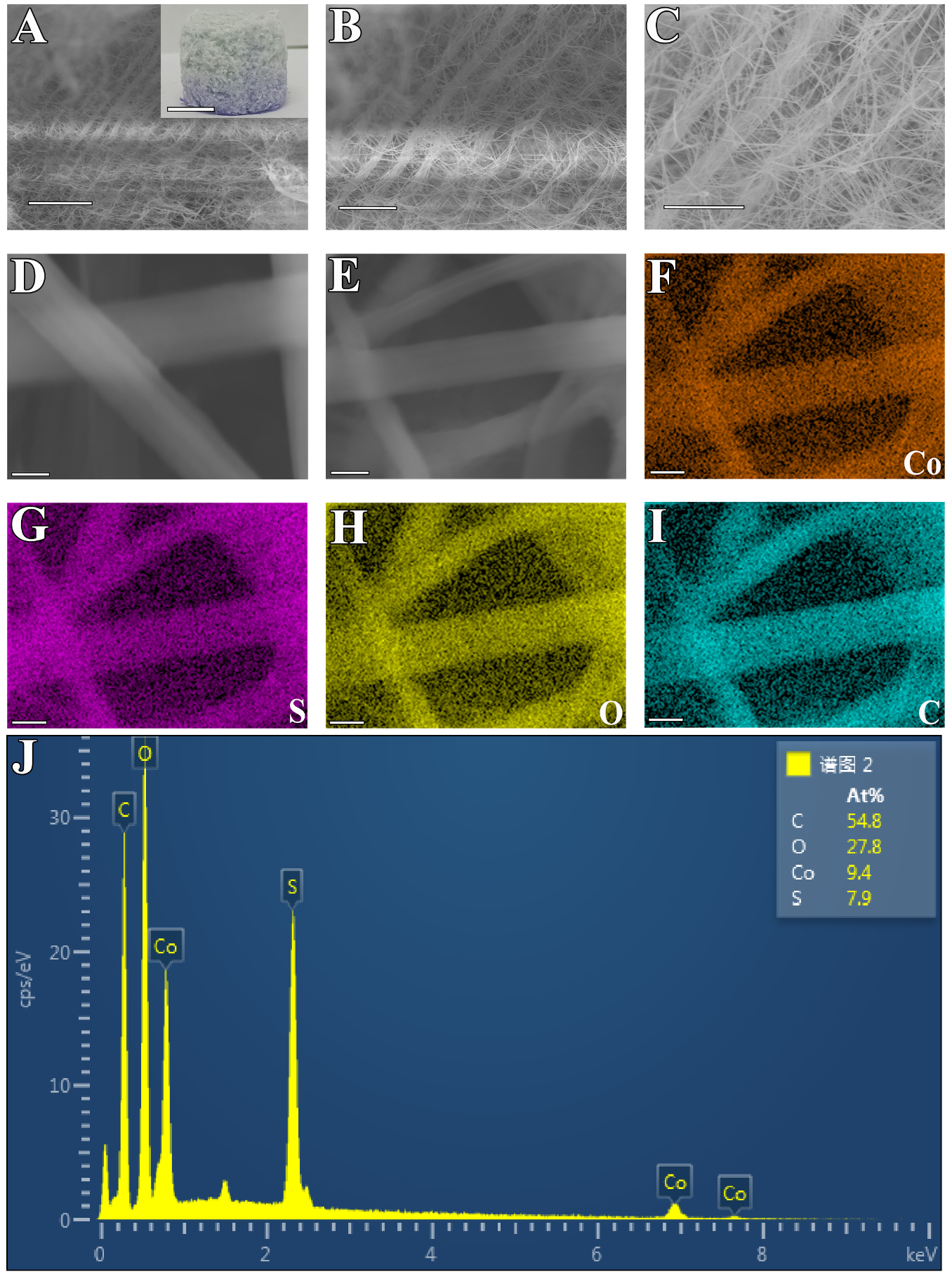


**Figure S6.** Morphological characterization of Co-MOF aerogels. (A-E) SEM images (scale bars for A, B, C, D, and E are 100 μm, 40 μm, 30 μm, 200 nm and 200 nm), inset of (A) is an optical image (scale bars: 1 cm); (F-I) SEM elemental mappings of Co, S, O, C (scale bars: 200 nm); (J) EDS elemental mapping images.


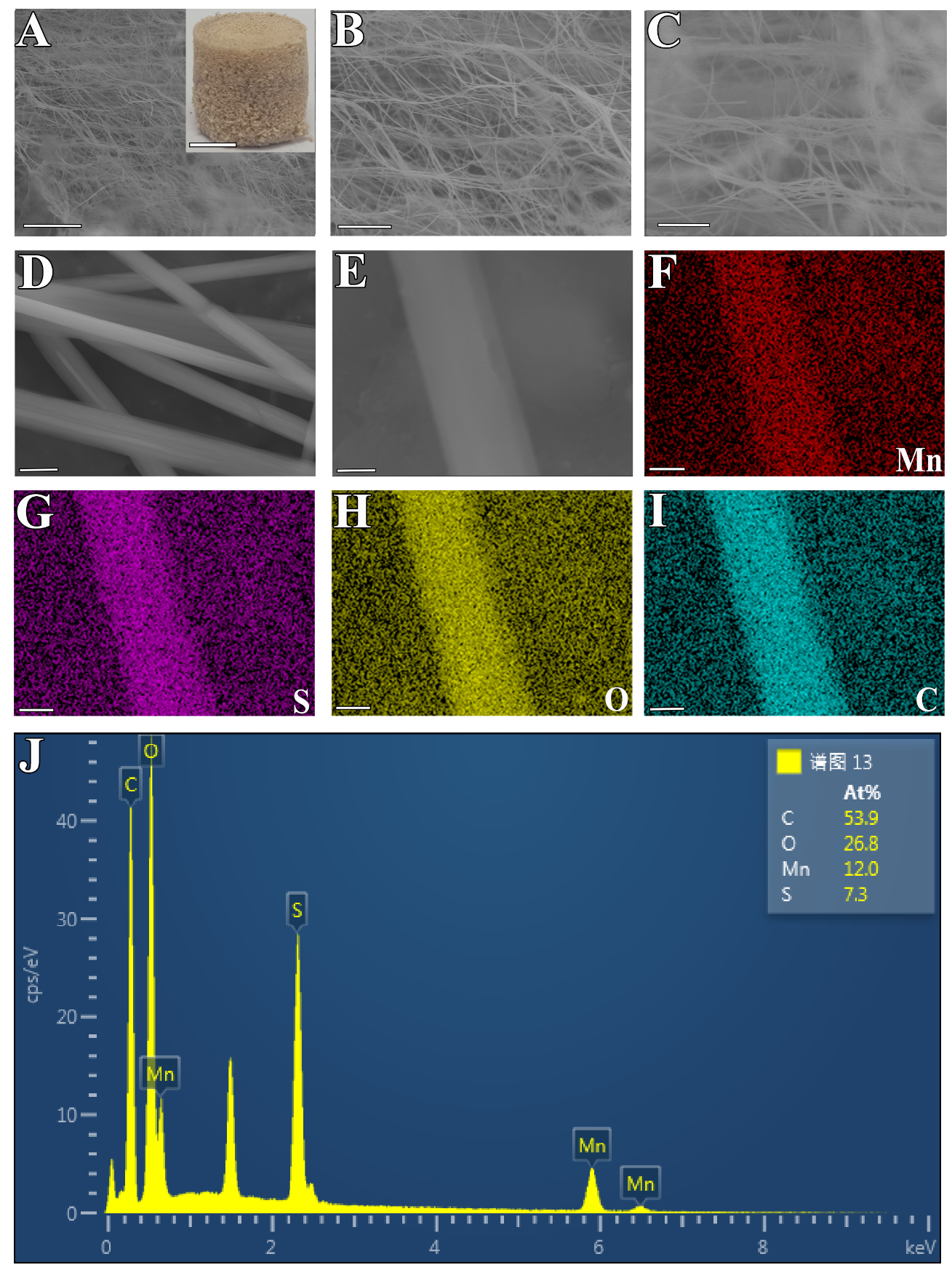


**Figure S7.** Morphological characterization of Mn-MOF aerogels. (A-E) SEM images (scale bars for A, B, C, D and E are 40 μm, 20 μm, 10 μm, 1 μm and 200 nm), inset of (A) is an optical image (scale bars: 1 cm); (F-I) SEM elemental mappings of Mn, S, O, C (scale bars: 200 nm); (J) EDS elemental mapping images.


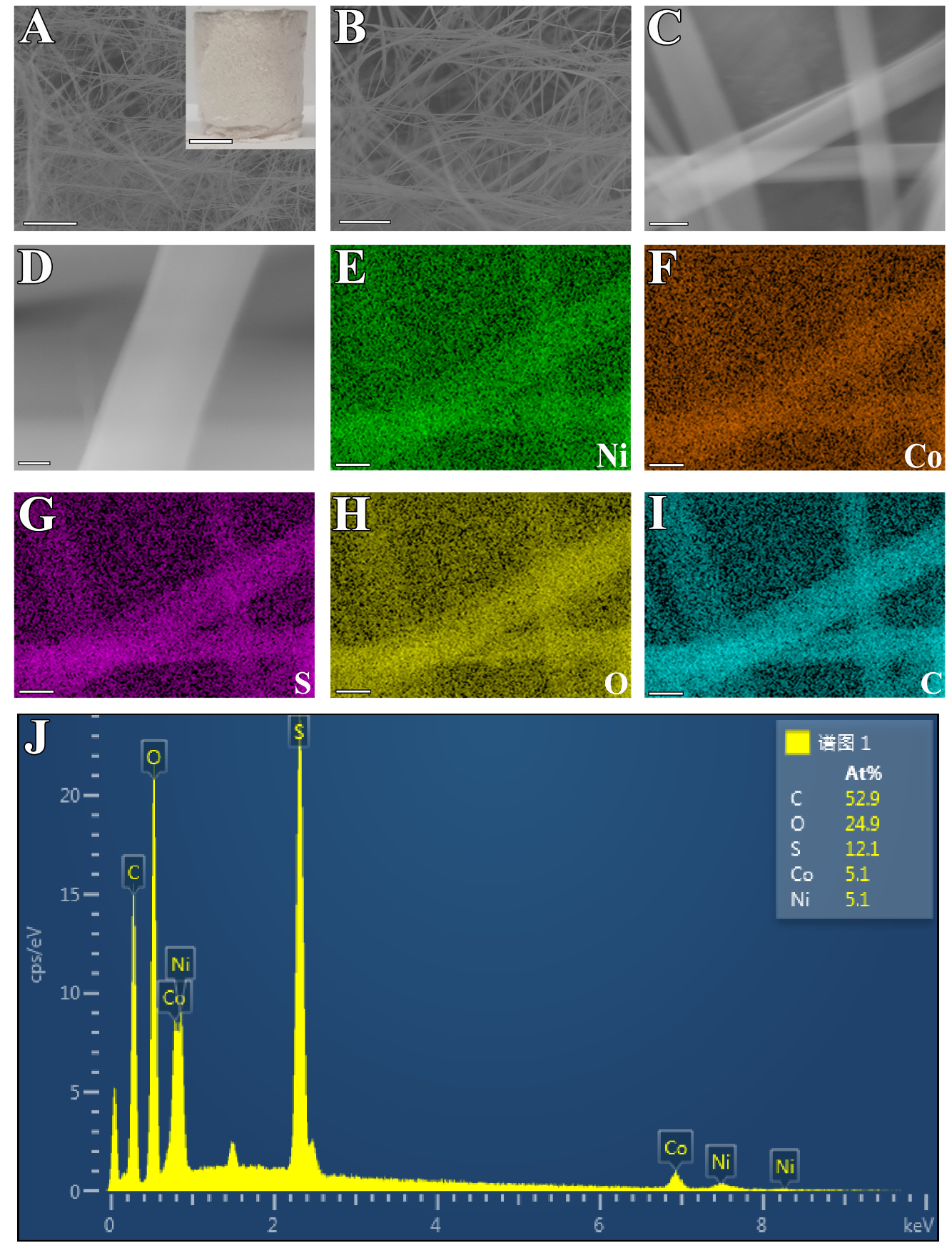


**Figure S8.** Morphological characterization of NiCo-MOF aerogels. (A-D) SEM images (scale bars for A, B, C and D are 20 μm, 10 μm, 200 nm and 100 nm), inset of (A) is an optical image (scale bars: 1 cm); (E-I) SEM elemental mappings of Ni, Co, S, O, C (scale bars: 200 nm); (J) EDS elemental mapping images.


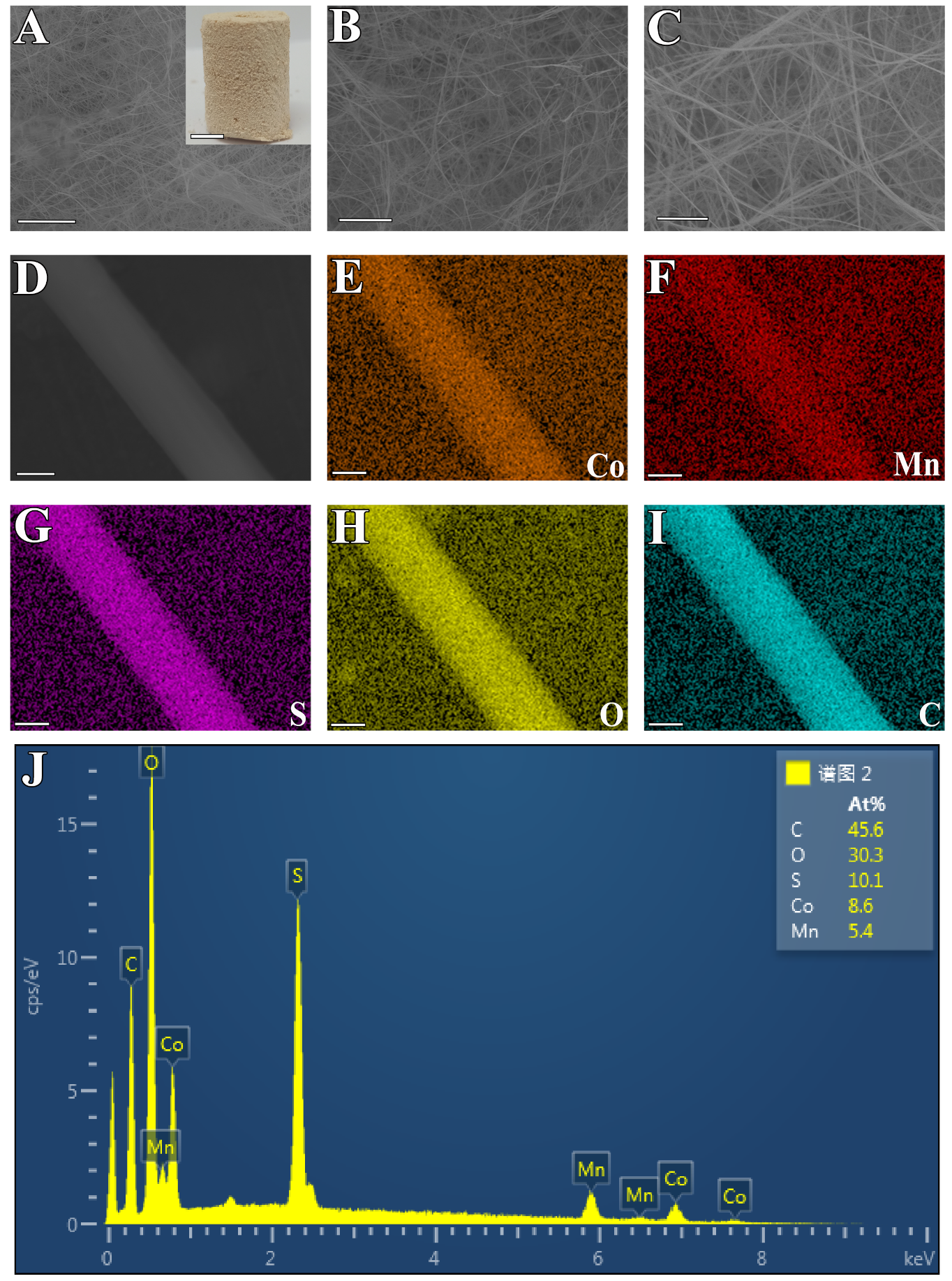


**Figure S9.** Morphological characterization of CoMn-MOF aerogels. (A-D) SEM images (scale bars for A, B, C and D are 40 μm, 20 μm, 10 μm and 200 nm), inset of (A) is an optical image (scale bars: 1 cm); (E-I) SEM elemental mappings of Co, Mn, S, O, C (scale bars: 200 nm); (J) EDS elemental mapping images.


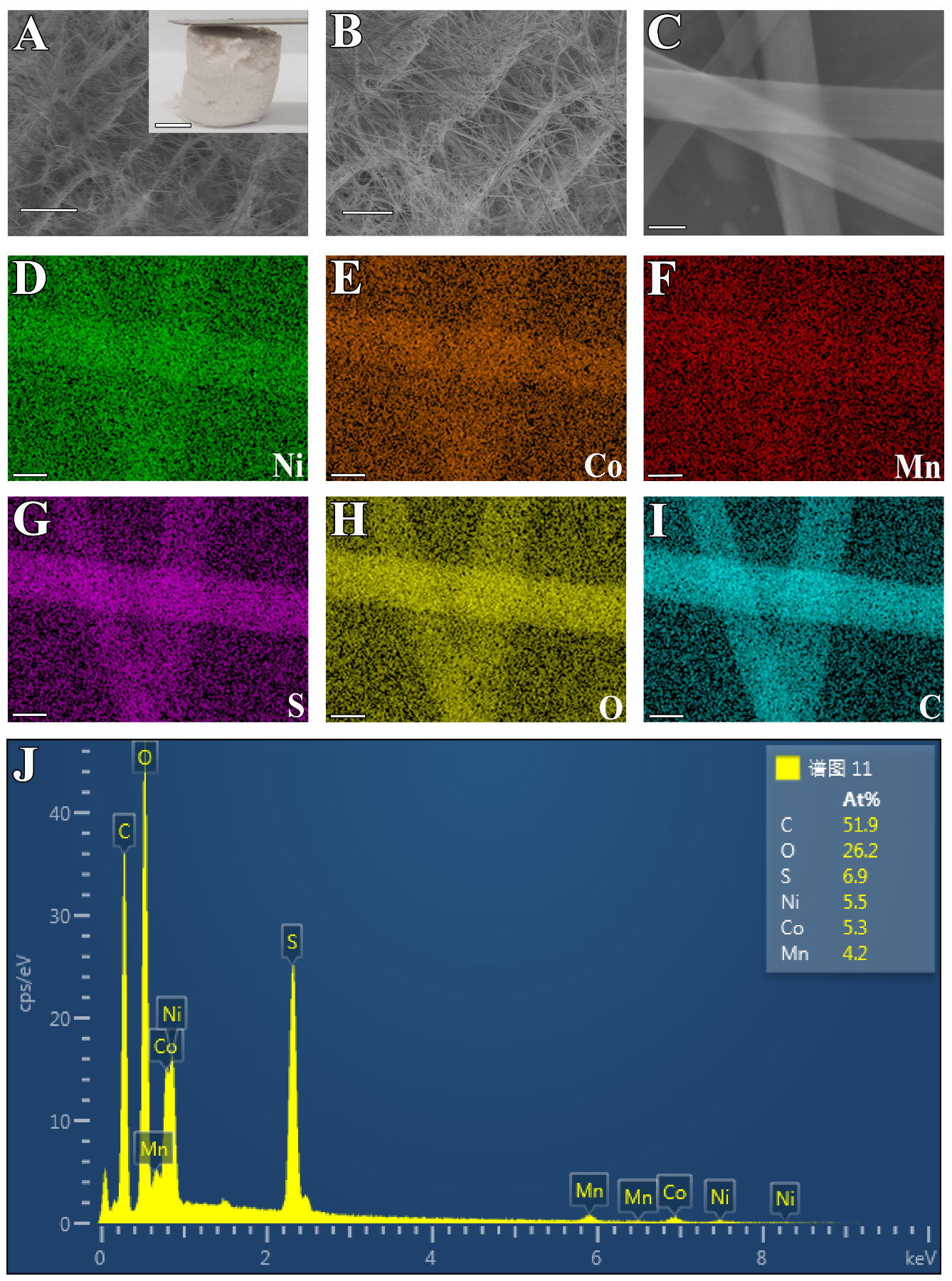


**Figure S10.** Morphological characterization of NiCoMn-MOF aerogels. (A-C) SEM images (scale bars for A, B and C are 40 μm, 20 μm and 200 nm), inset of (A) is an optical image (scale bars: 1 cm); (D-I) SEM elemental mappings of Ni, Co, Mn, S, O, C (scale bars: 200 nm); (J) EDS elemental mapping images.

*
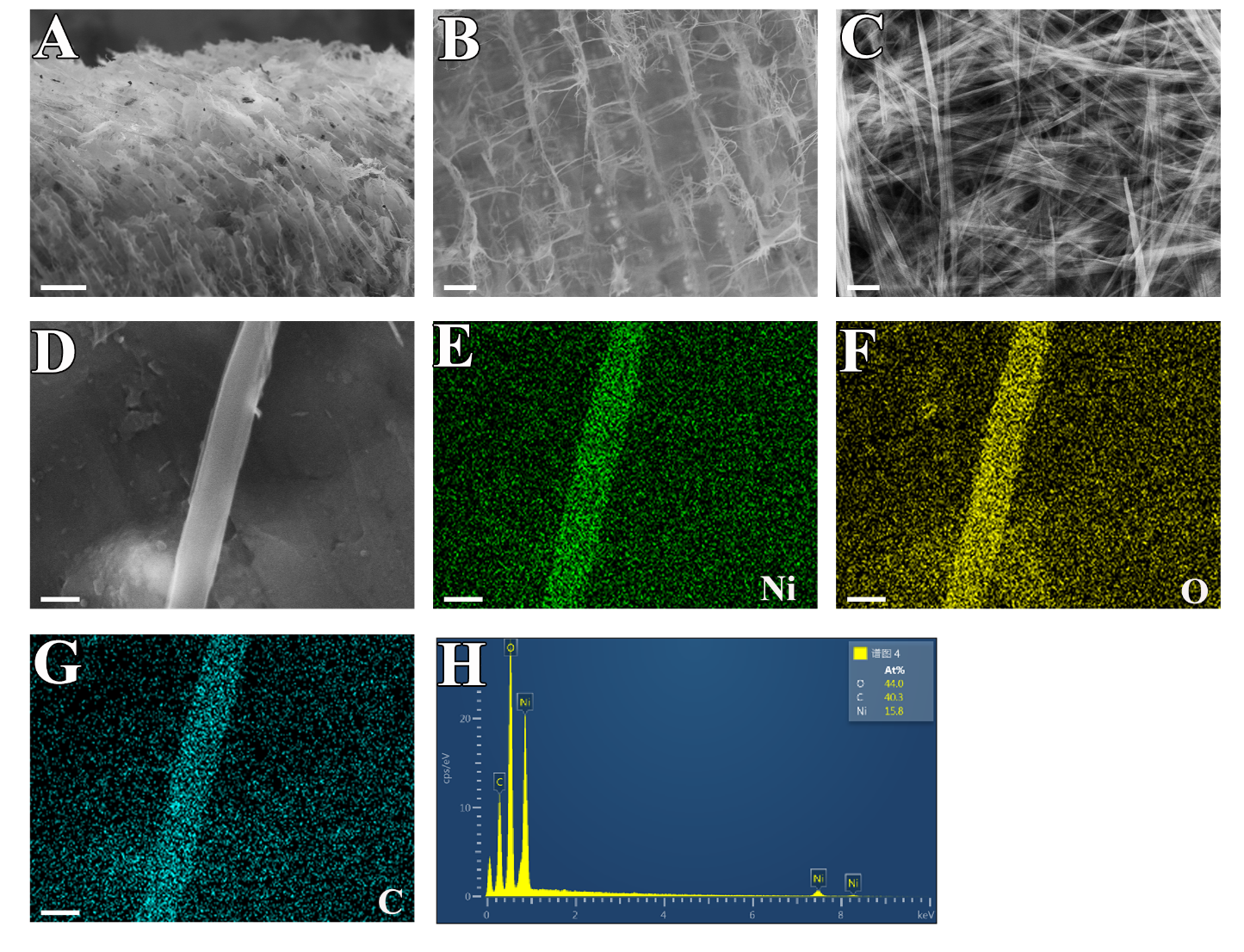
*

**Figure S11.** Morphological characterization of Ni-MIL-77 aerogels using glutaric acid as an organic ligand. (A-C) SEM images (scale bars for A, B and C are 100 μm, 10 μm and 500 nm); (D-G) SEM and elemental mappings of Ni, O and C (scale bars: 200 nm); (H) EDS elemental mapping images.

**Supplementary note.**

Ni-MIL-77 aerogel is present as a solid green cylinder of ~4 cm height (Figure. S22). The macroscopic aerogel is made of ordered 2D sheet array with the adjacent sheet spacing of approximately 25 μm (Figure. S11A,B). These nanobelts have a typical width of ~200 nm, and length over several micrometers (Figure. S11C). Further, SEM element mapping shows the homogeneously distribution of Ni, O and C elements throughout nanobelts (Figure. S11D-H).


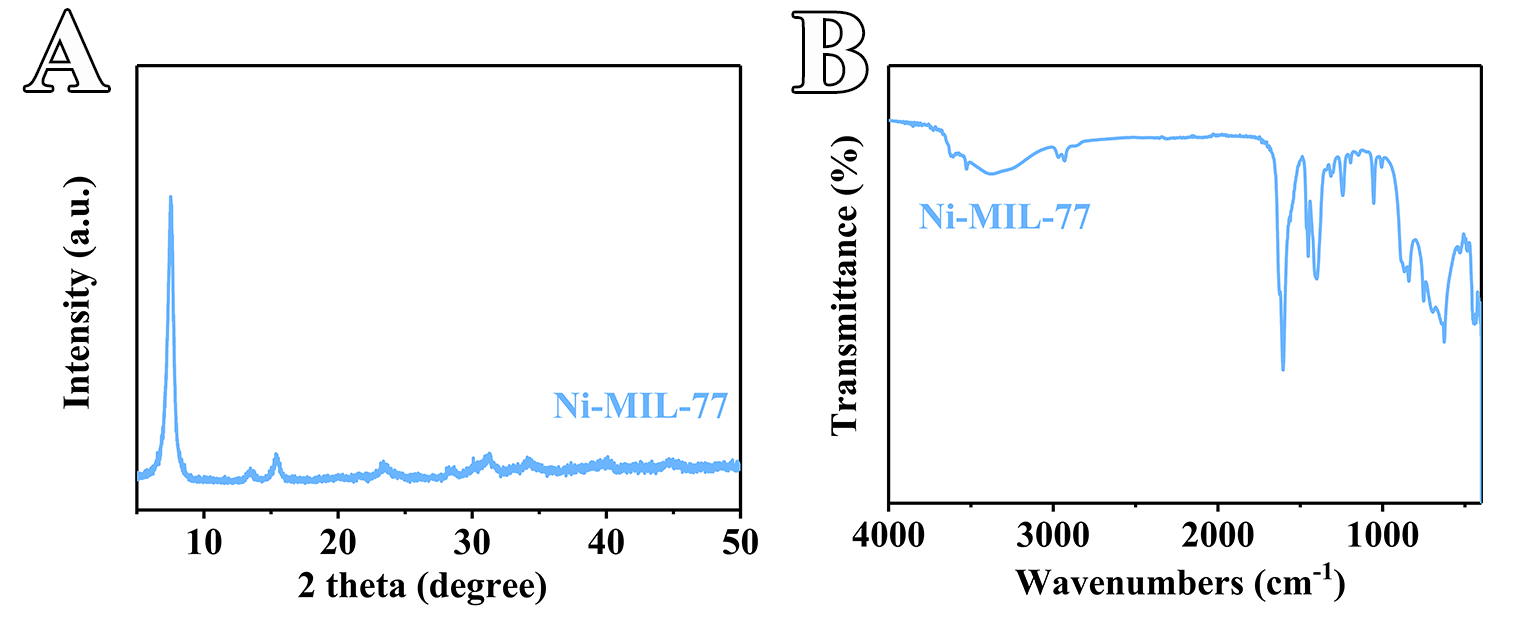


**Figure S12.** (A) XRD pattern and (B) FT-IR spectra of Ni-MIL-77 using glutaric acid as an organic ligand, both of which are consistent with previous work.[^10^](#_ENREF_10)


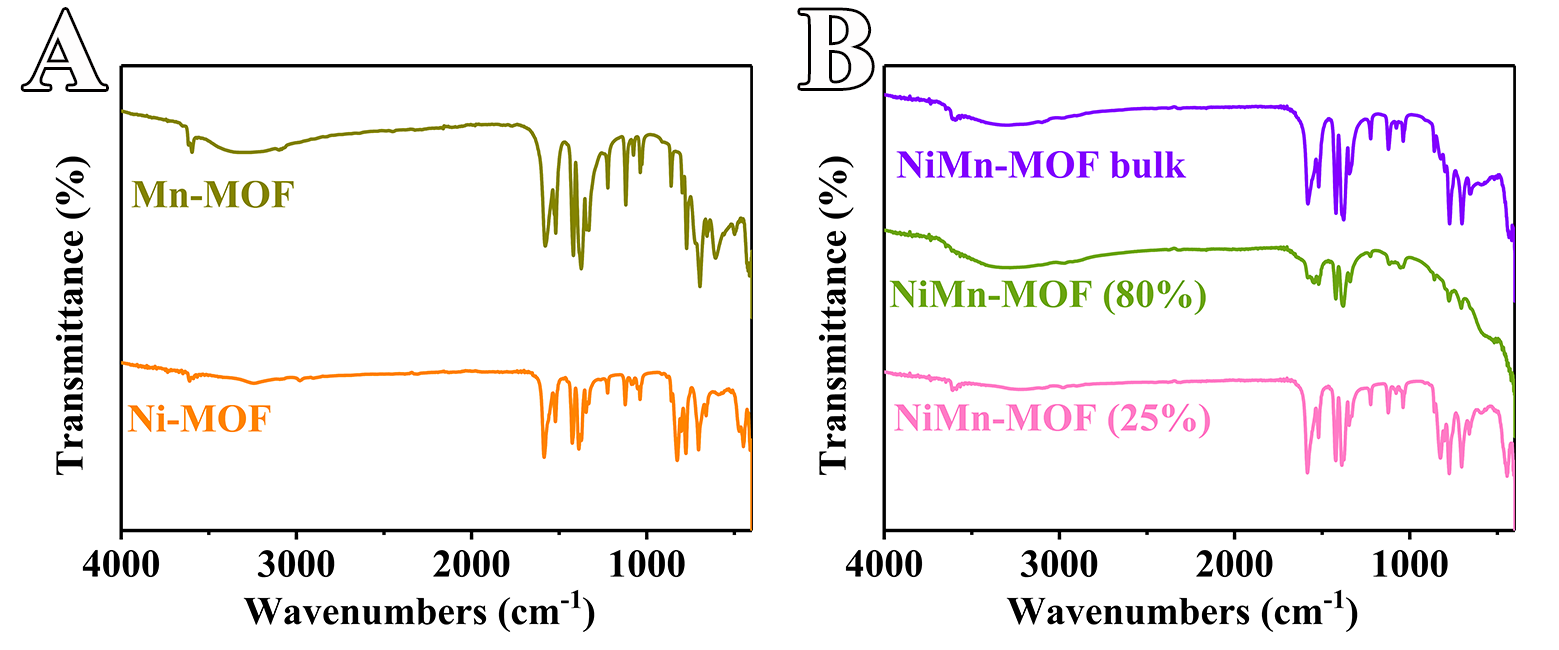


**Figure S13.** FT-IR spectra of Mn-MOF, Ni-MOF, NiMn-MOF (powder), NiMn-MOF (80% of Mn) and NiMn-MOF (25% of Mn).

**Supplementary note.**

In Figure. S13, vibration bands of organic ligand unit at 1421 and 1037 cm^-1^, carboxylate groups (COO^-^) coordinated with Ni^2+^ or Fe^3+^ at 1582, 1520, 1377 and 774 cm^-1^ (Fig. 2J) are observed.^12,13^ Further, as compared with the organic ligand in Fig. 2J, the acidic carbonyl group (C=O) and non-ionized carboxyl group (C-OH) disappeared in NiMn-MOF, indicating that the organic ligand has been completely deprotonated and formed MOF with Ni or Mn ions.


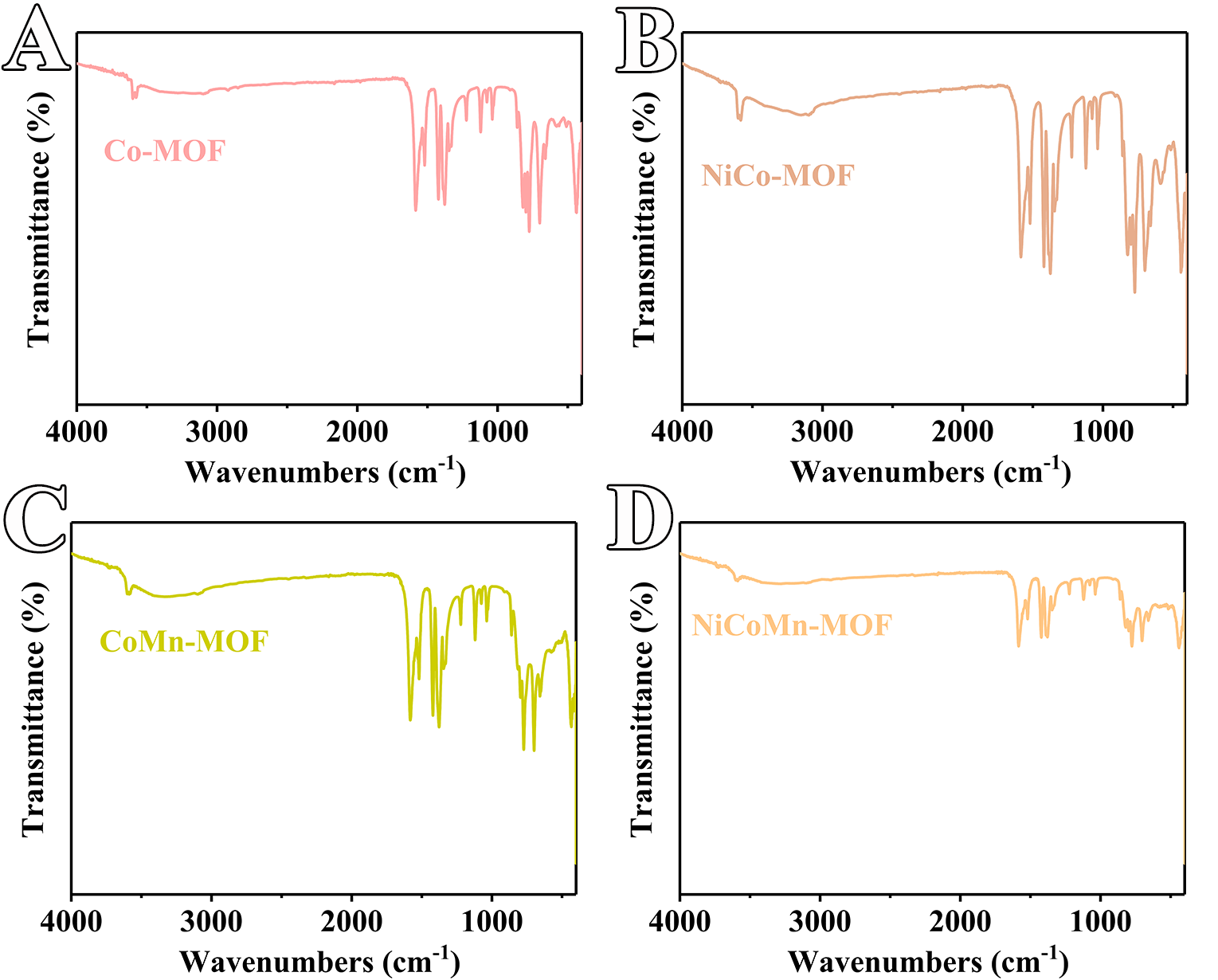


**Figure S14.** FT-IR spectra of (A) Co-MOF, (B) NiCo-MOF, (C) CoMn-MOF and (D) NiCoMn-MOF.


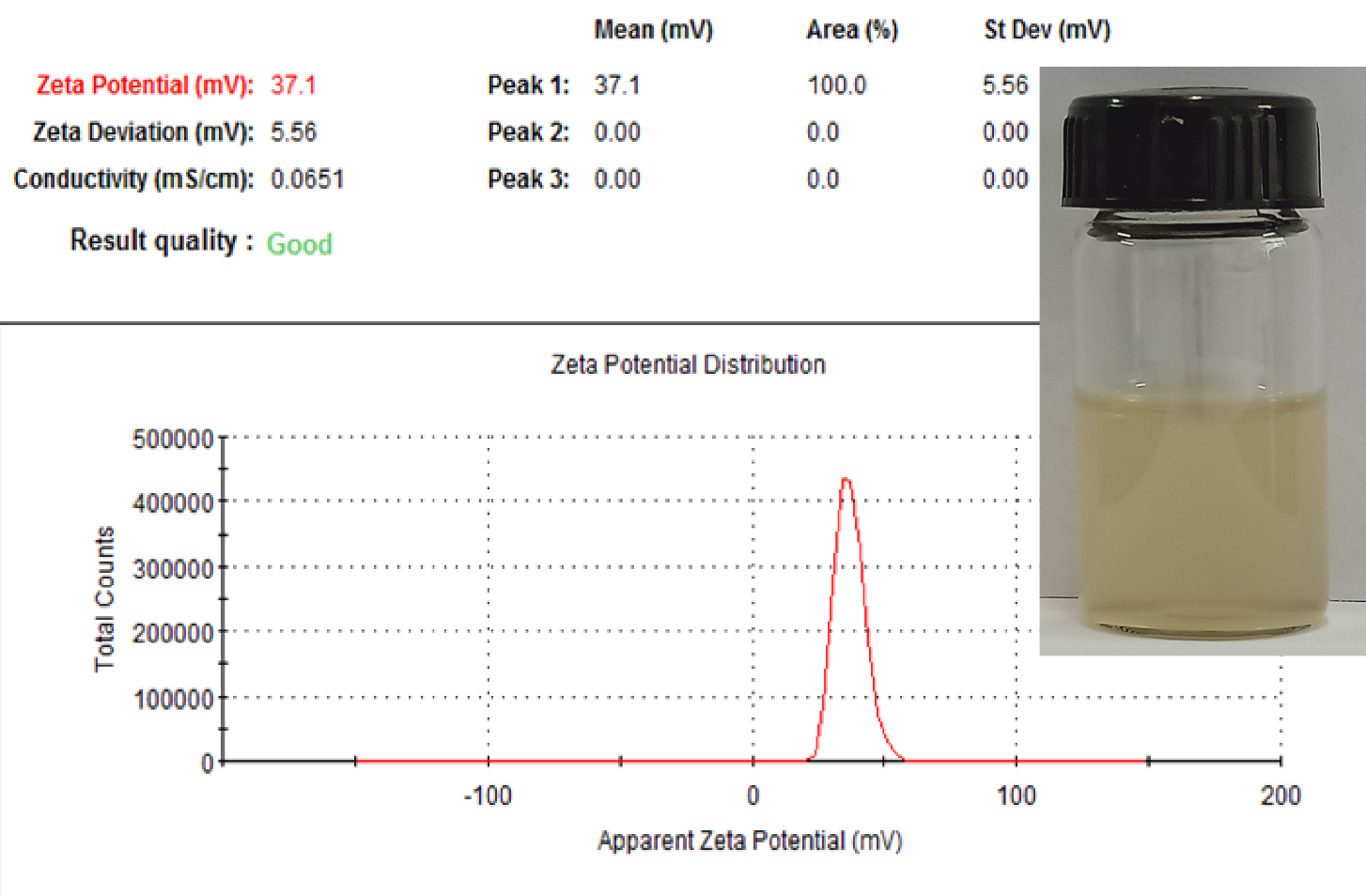


**Figure S15.** Zeta potential of aqueous solution of NiMn-MOF nanobelts.


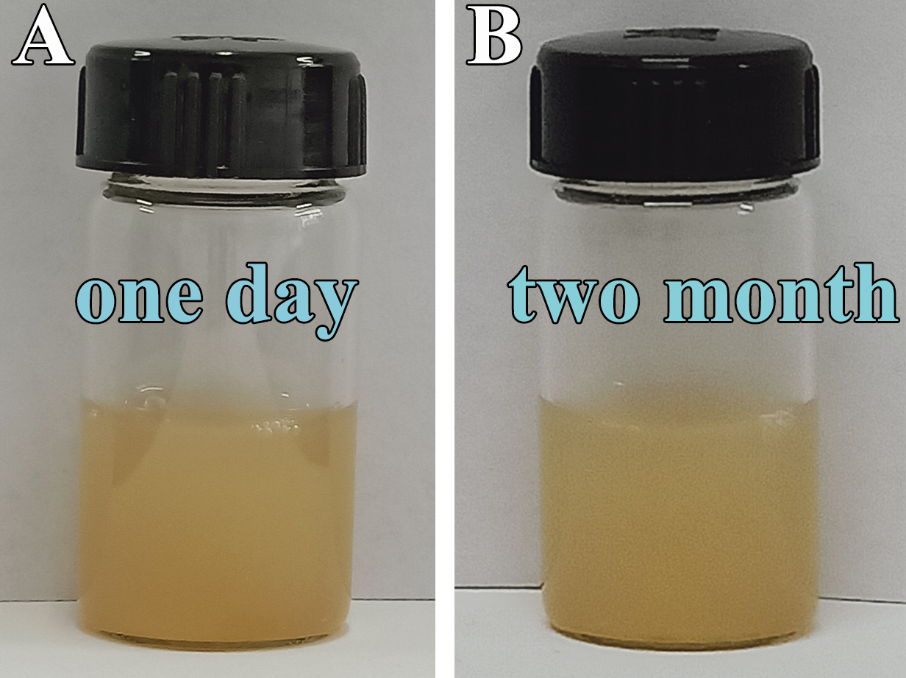


**Figure S16.** Optical photographs of aqueous solutions of NiMn-MOF nanobelts after standing still for different time.


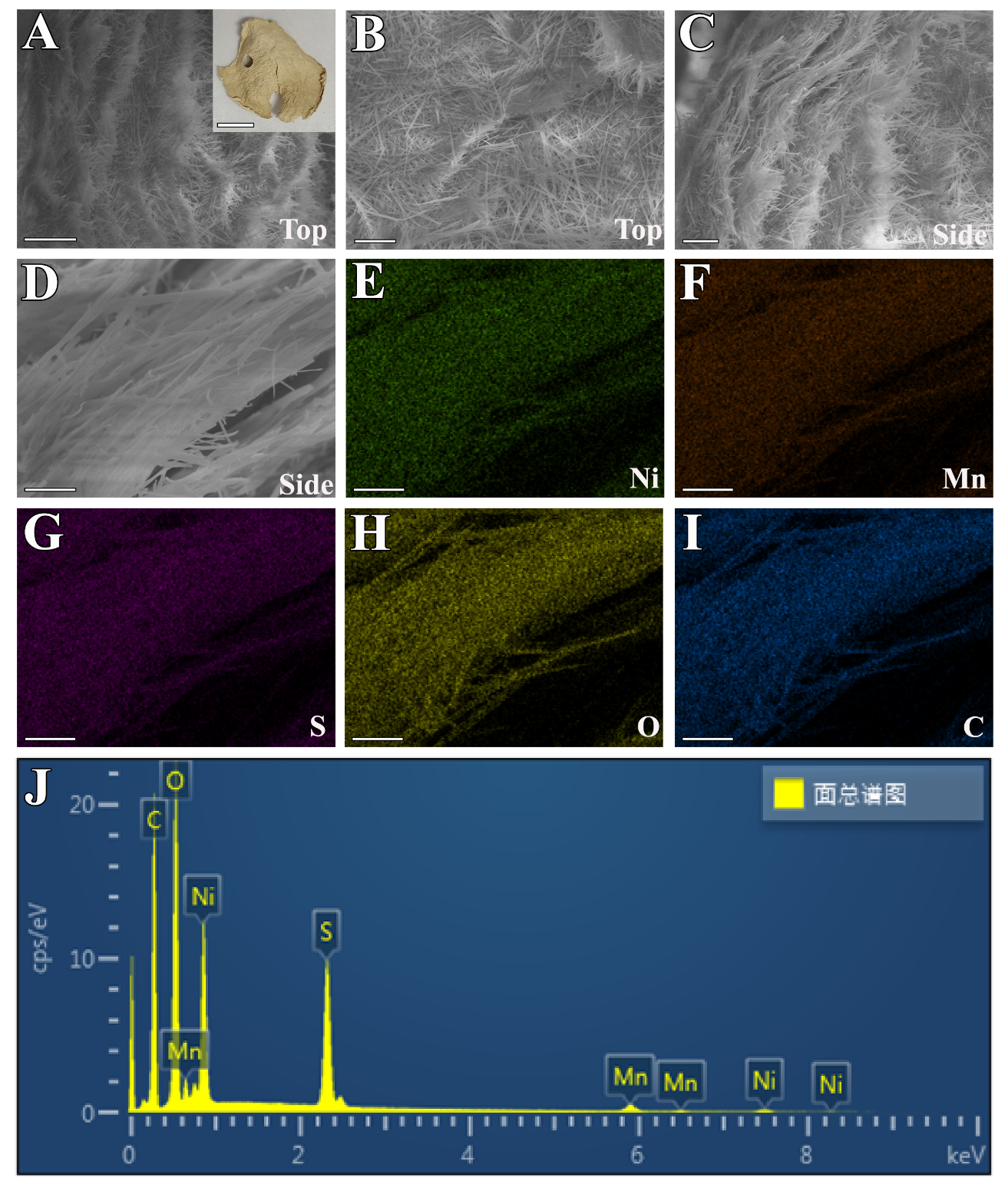


**Figure S17.** Morphological characterization of bulk NiMn-MOF obtained by directly drying nanobelts at 60 ^o^C in an oven. (A-D) SEM images (scale bars for A, B, C and D are 20, 10, 10, and 2 μm), inset of (A) is an optical image (scale bars: 1 cm); (E-I) SEM elemental mappings of Ni, Mn, S, O, C (scale bars: 2 μm); (J) EDS elemental mapping images.


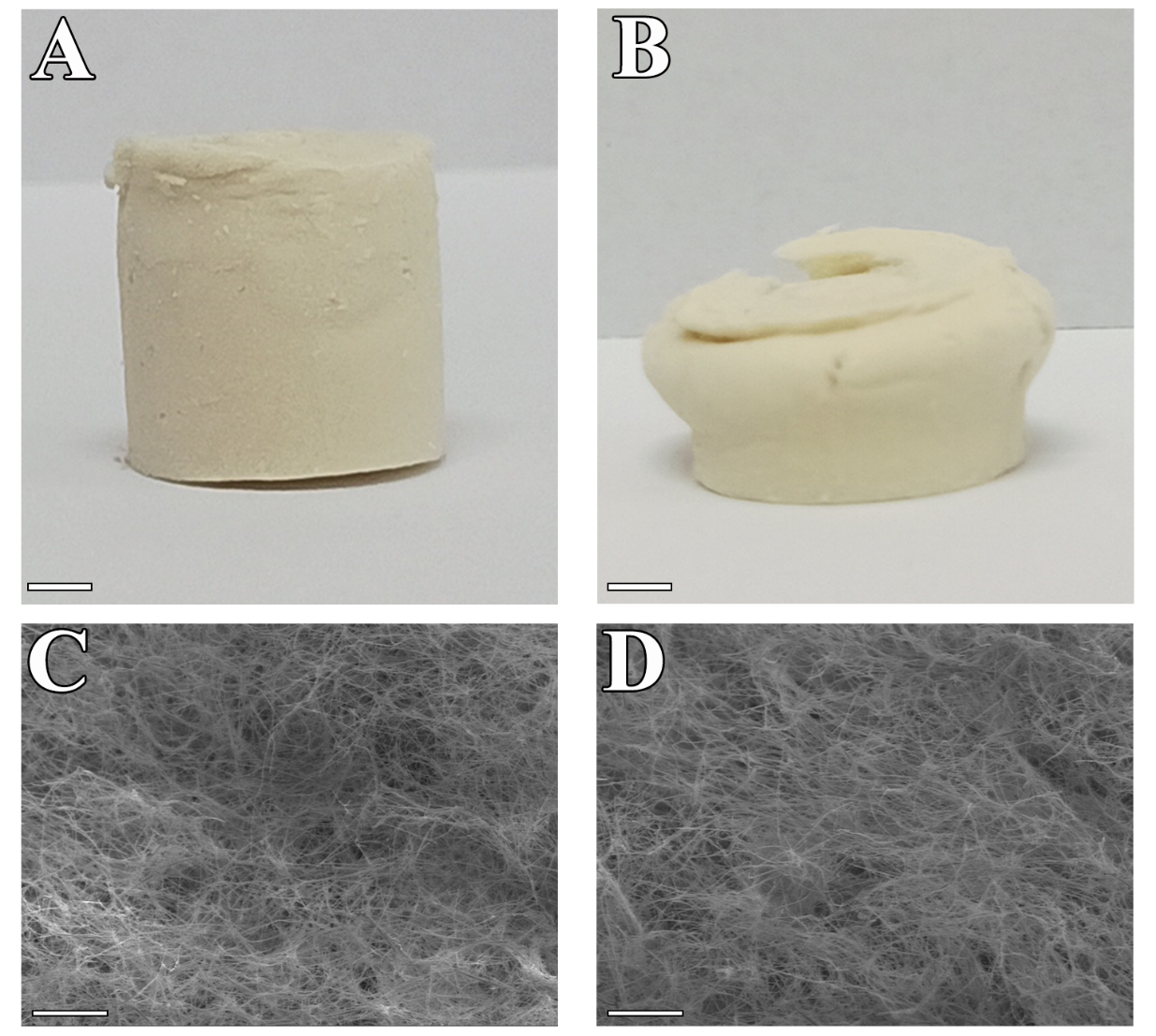


**Figure S18.** Morphological characterization of NiMn-MOF aerogels obtained by rapid liquid nitrogen freezing (freezing rate: 50 ^o^C min^-1^). (A, B) morphology before and after compression (without mechanical flexibility, scale bars: 2 cm); (C, D) SEM images (scale bars are 30 μm).


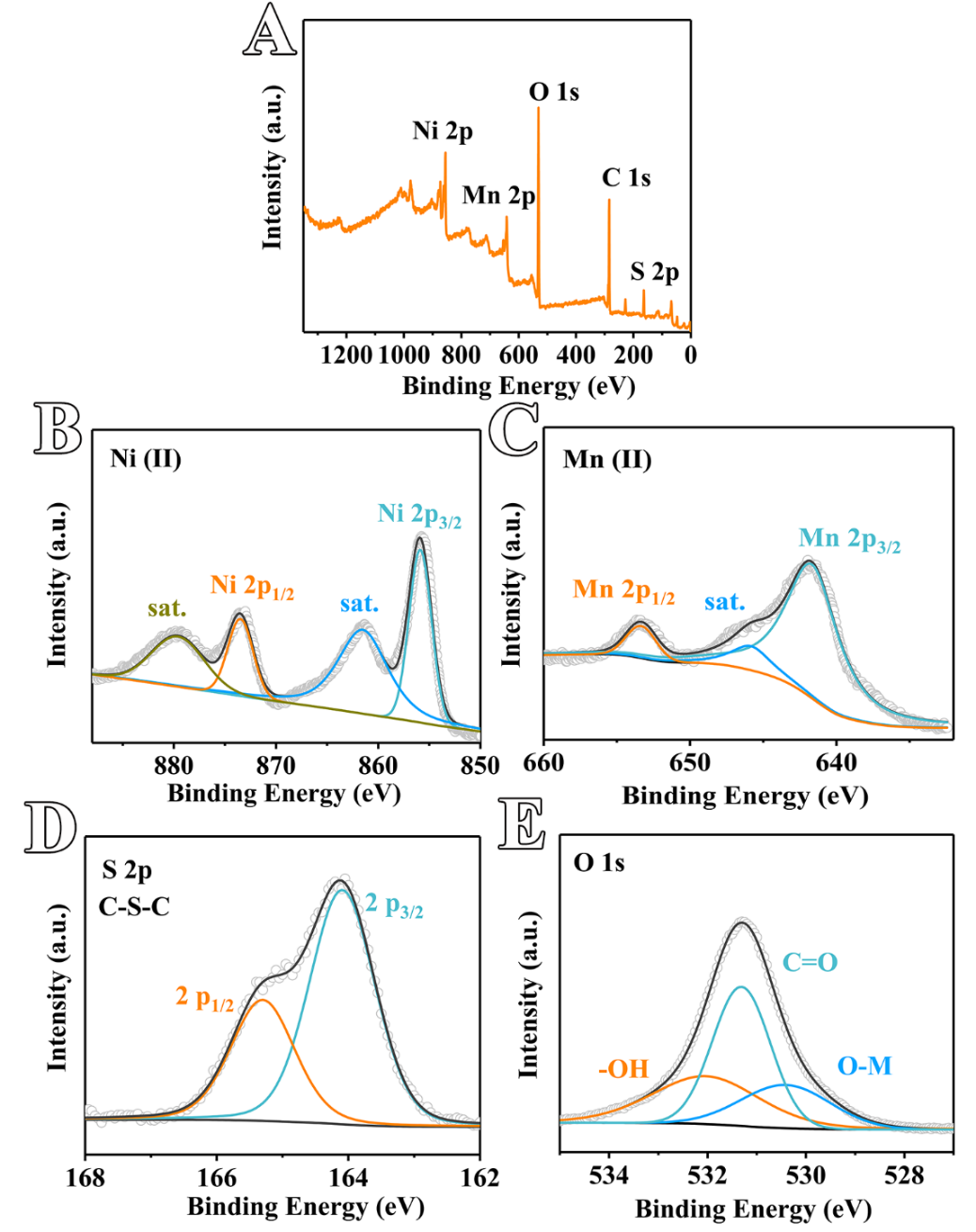


**Figure S19. XPS spectra.** (A) Survey scan of NiMn-MOF, (B) Ni 2p, (C) Mn 2p, (D) S 2p, C-S-C and (E) O 1s regions of NiMn-MOF.

**Supplementary note.**

XPS Ni 2p profile has been deconvoluted into Ni 2p_3/2_ at 855.9 eV, Ni 2p_1/2_ at 873.5 eV and their satellites at 861.5 (Ni 2p_3/2_) and 879.6 eV (Ni 2p_1/2_, Figure S19B). Therefore, the oxidation state of the Ni species is determined to be +2. Similarly, XPS Mn 2p profile exhibits Mn 2p_3/2_ at 641.7 eV, Mn 2p_1/2_ at 653.3 eV, and satellites at 645.8 eV, which identify +2 oxidation state for Mn inside NiMn-MOF (Figure S19C). Further, we analyzed the XPS S 2p_3/2_ at 164.1 eV and S 2p_1/2_ at 165.3 eV that indicate the C-S-C chemical bond in the organic ligand (Figure S19D);^11^ and the peaks of the O1s at 530.4, 531.3 and 532 eV attributed to O-M (Ni, Mn), C=O and -OH from metal nodes of MOF materials (Figure S19E).


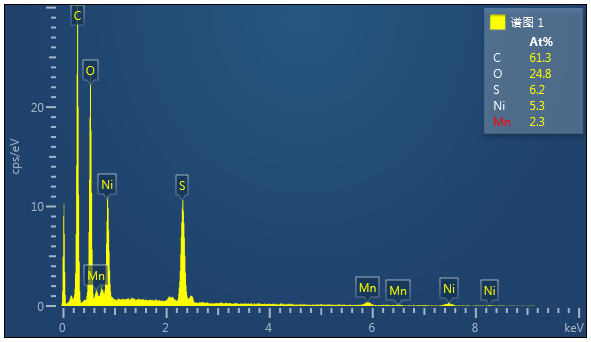


**Figure S20.** EDS of NiMn-MOF**.**


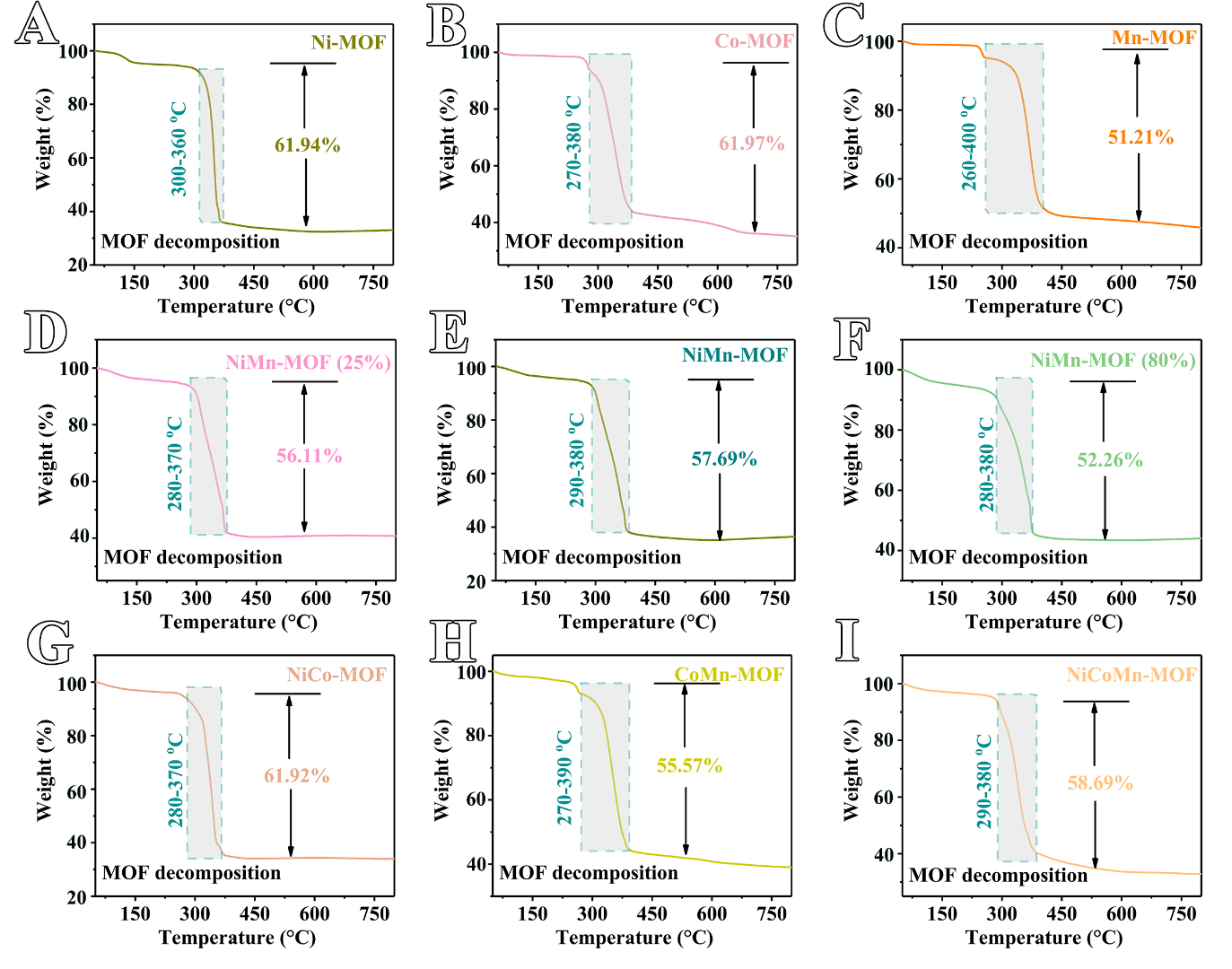


**Figure S21.** TGA profiles of (A) Ni-MOF, (B) Co-MOF, (C) Mn-MOF, (D) NiMn-MOF (25%), (E) NiMn-MOF, (F) NiMn-MOF (80%), (G) NiCo-MOF, (H) CoMn-MOF and (I) NiCoMn-MOF.

Taking NiMn-MOF as an example, the combination of element analysis techniques (EA, Figures. S5-10,S20), XPS (Figure. S19), ICP-OES (Table S1) and TGA (Figure. S21) shows its chemical formula as: Ni_0.62_Mn_0.38_(C_5_H_3_SO_2_)(C_2_H_6_O)_2_. Similarly, we summarize the chemical formulas of other MOF as follows:

Ni-MOF: Ni(C_5_H_3_SO_2_)(C_2_H_6_O)_2_

Co-MOF: Co(C_5_H_3_SO_2_)(C_2_H_6_O)_2_

Mn-MOF: Mn(C_5_H_3_SO_2_)(C_2_H_6_O)_2_

NiCo-MOF: Ni_0.5_Co_0.5_(C_5_H_3_SO_2_)(C_2_H_6_O)_2_

CoMn-MOF: Co_0.61_Mn_0.39_(C_5_H_3_SO_2_)(C_2_H_6_O)_2_

NiCoMn-MOF: Ni_0.36_Co_0.35_Mn_0.28_(C_5_H_3_SO_2_)(C_2_H_6_O)_2_


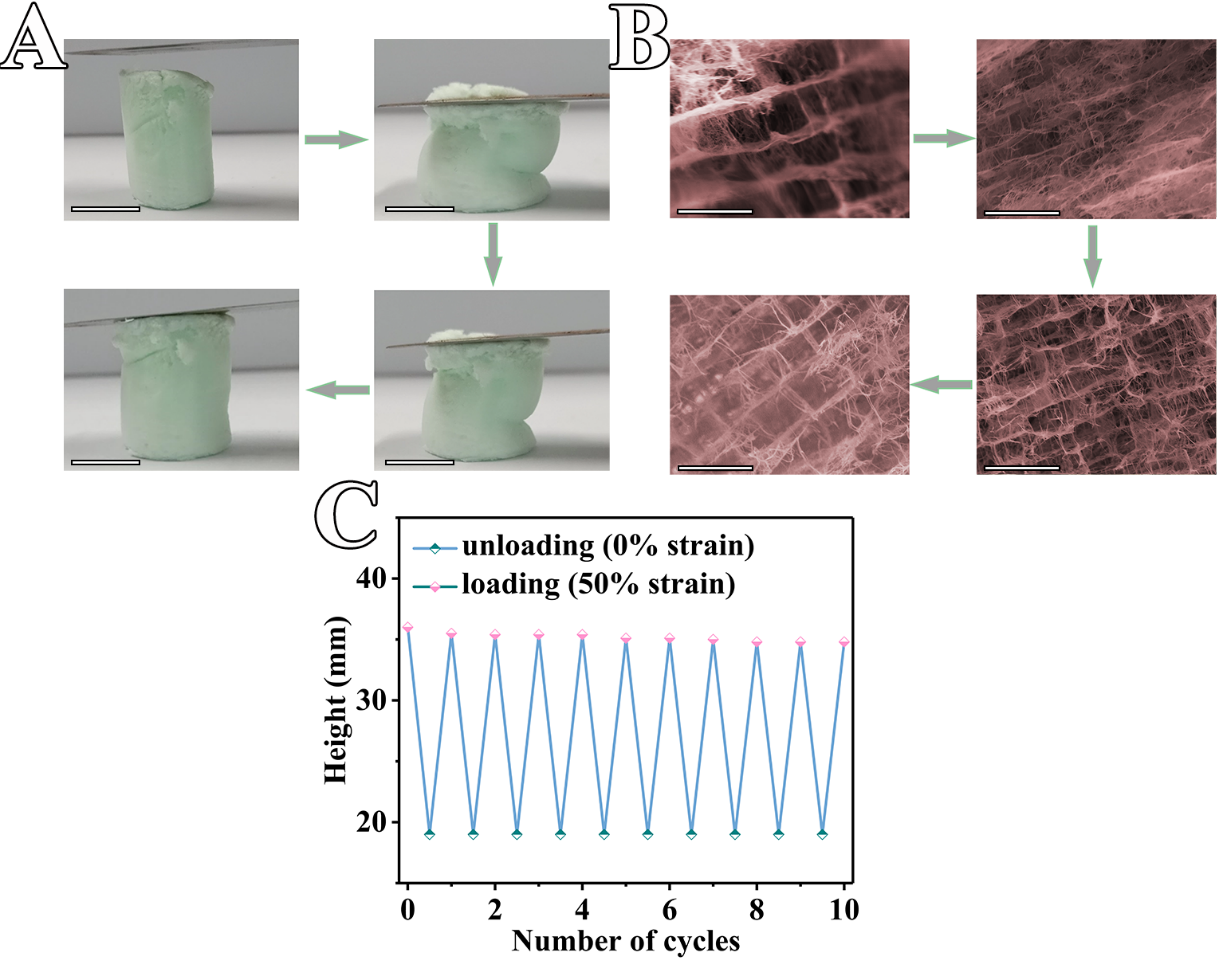


**Figure S22.** **Mechanical property of Ni-MIL-77 aerogels (glutaric acid as the organic ligand).** (A) Optical images (scale bars: 2 cm); (B) SEM images (scale bars: 30 μm); (C) Compression-unloading cycles.


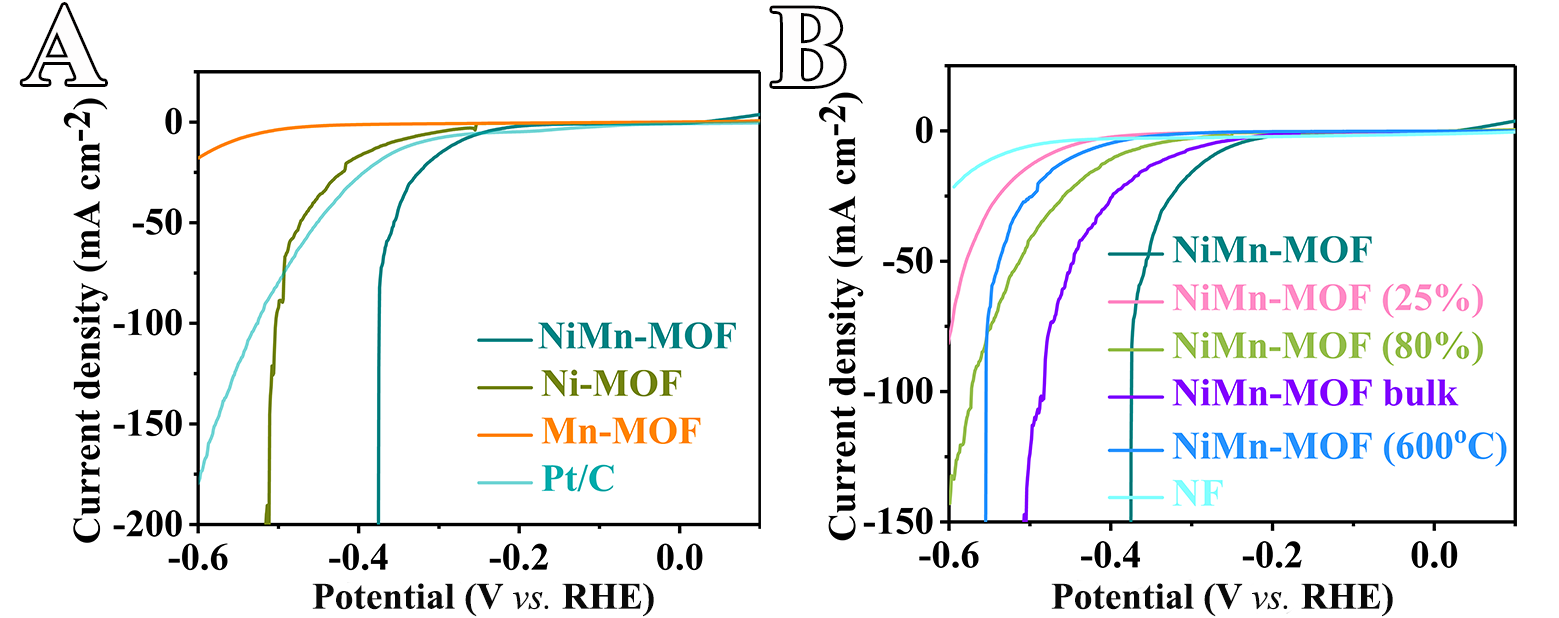


**Figure S23.** LSV curves of NiMn-MOF (A) and other comparison electrodes (B) for HER at 5 mV s^-1^ with 85% *iR*-compensation in 3 wt% NaCl electrolyte.


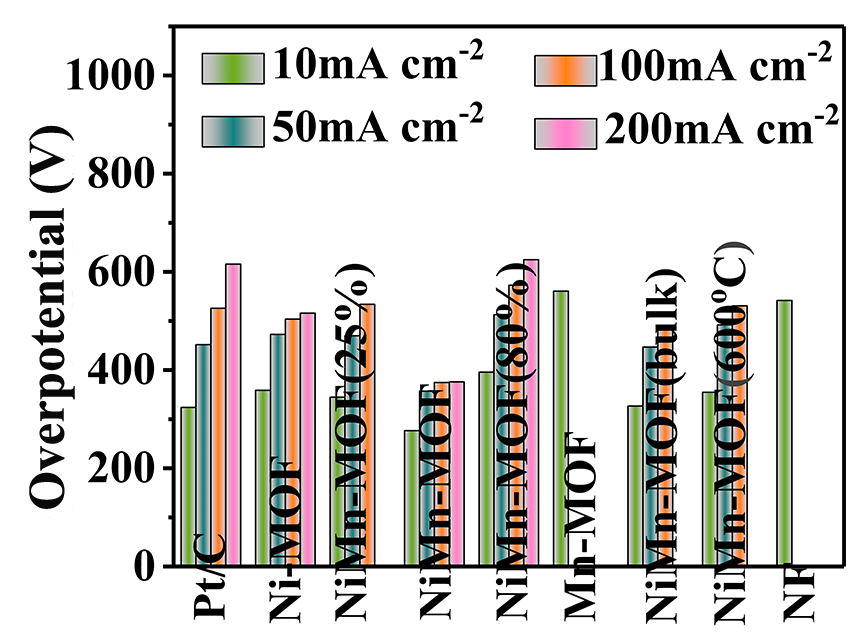


**Figure S24.** Required overpotentials of NiMn-MOF and other comparison electrodes to achieve different current densities in 3 wt% NaCl electrolyte.

**Supplementary note.**

The electrocatalytic test was conducted in a three-electrode cell containing 3 wt% NaCl solution with 85% *iR*-compensation. Firstly, LSVs demonstrates outstanding activities for NiMn-MOF as comparison to other samples (Figure. S23). To afford a current density of 10 mA cm^-2^, NiMn-MOF only requires an overpotential of 277 mV, which is smaller than Ni-MOF (359 mV), NiMn-MOF (25%, 483 mV), NiMn-MOF (80%, 396 mV) and Mn-MOF (561 mV), NiMn-MOF powder (327 mV), NiMn-MOF calcinated at 600 ^o^C (448 mV) and Pt/C (324 mV, Figures. S23,S24 and Table S5). Even under the high current density of 200 mA cm^-2^, the HER overpotential of NiMn-MOF is only 376 mV (Figures. S23, S24 and Table S5). By comparison to literature (Table S9), Our NiMn-MOF is among the most active reported electrocatalysts reported thus far.


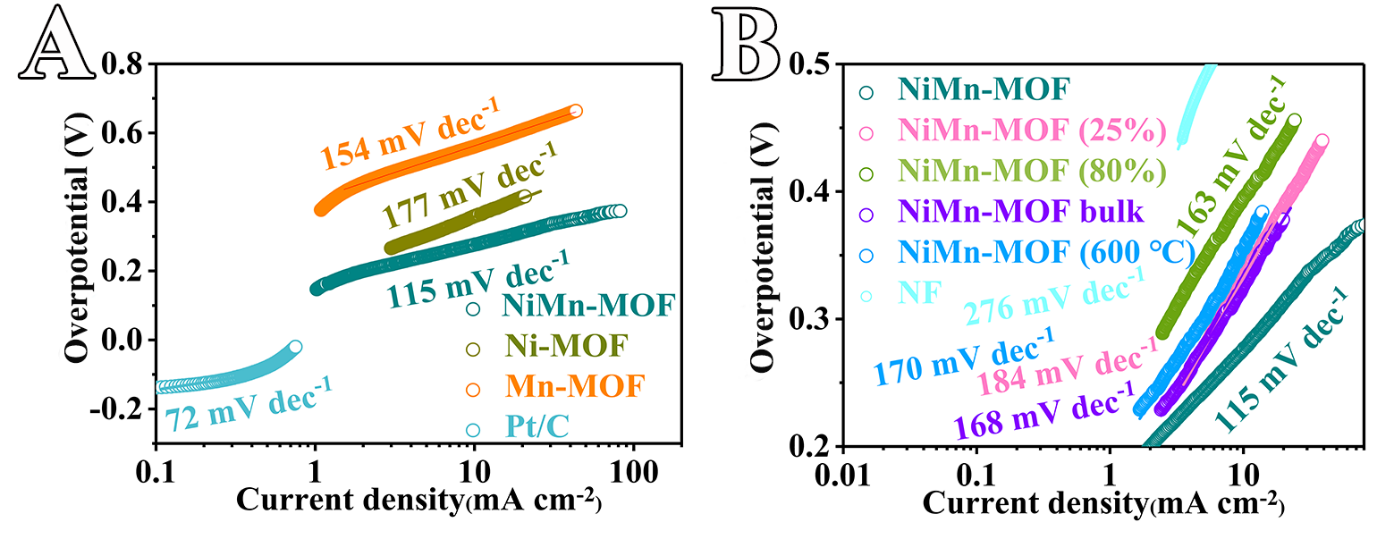


**Figure S25.** Tafel plots of NiMn-MOF (A) and other electrodes (B) during HER process in the 3 wt% NaCl electrolyte.


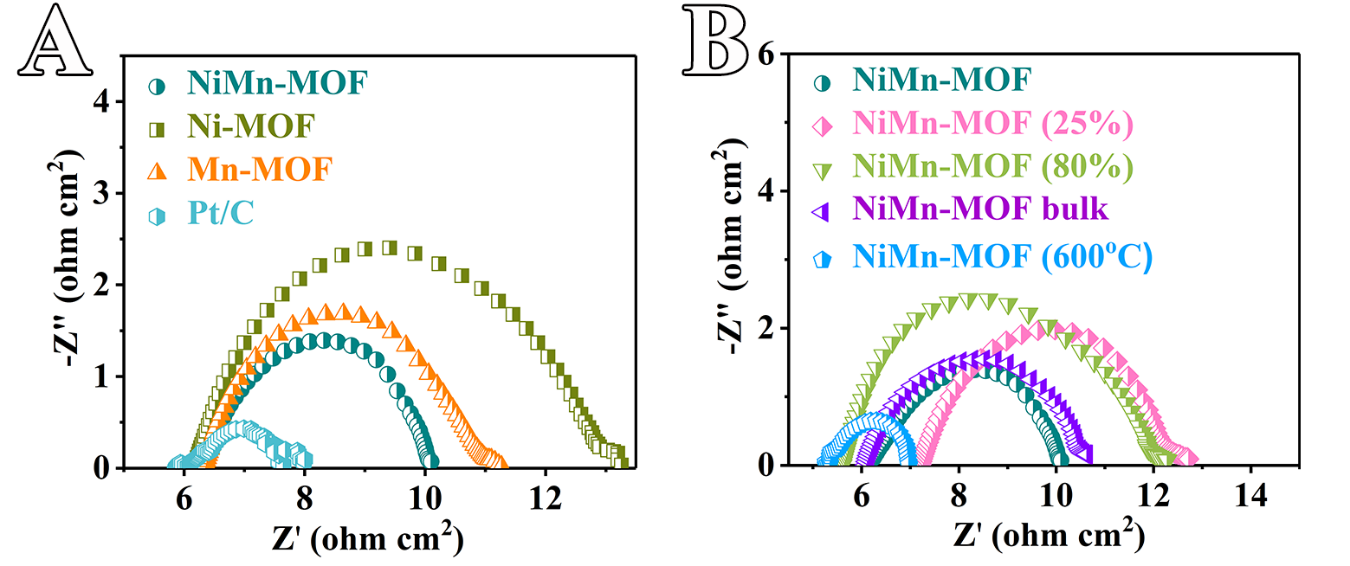


**Figure S26.** EIS plots of NiMn-MOF (A) and other comparison electrodes (B) in 3 wt% NaCl electrolyte. According to Figures. S25,S26 and Table S5, the catalyst electrodes show excellent reaction kinetics, as verified by the similar Tafel slopes and charge-transfer resistance (R_ct_) from EIS analysis compared with other comparative samples.


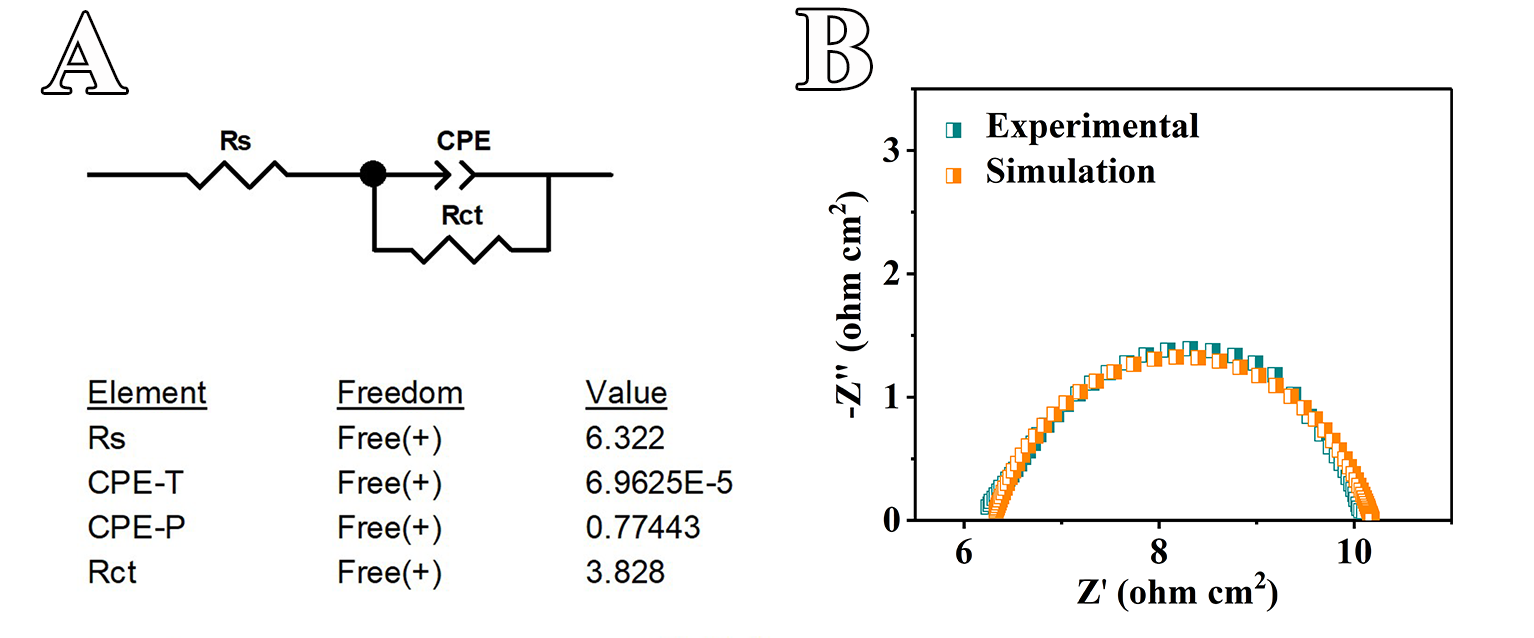


**Figure S27.** (A) Simulated equivalent circuit model according to the EIS plot of NiMn-MOF; (B) Experimental and simulated EIS plots of NiMn-MOF in 3 wt% NaCl electrolyte.

**Supplementary note.**

The experimental data for NiMn-MOF is consistent with simulation results. Consequently, the equivalent circuit is consisted of a solution resistance (R_s_), a constant phase element (CPE) and a charge transfer resistance (R_ct_). Similarly, all other EIS results have been simulated.


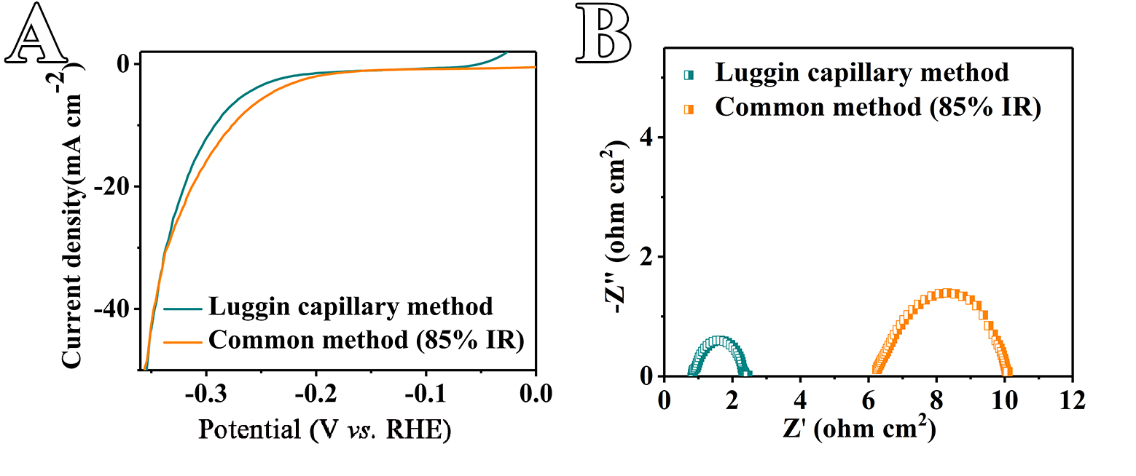


**Figure S28.** Comparison performance of NiMn-MOF by using a luggin capillary method and 85% *iR*-compensation method. (A) LSV curves; (B) EIS plots. EIS shows that luggin capillary method can indeed reduce the solution resistance (R_s_) from 6.1 to 0.82 ohm cm^2^. Nevertheless, LSV shows they demonstrate similar current densities, especially at high potentials. Therefore, the *iR*-compensation method is as reliable as the luggin capillary method.


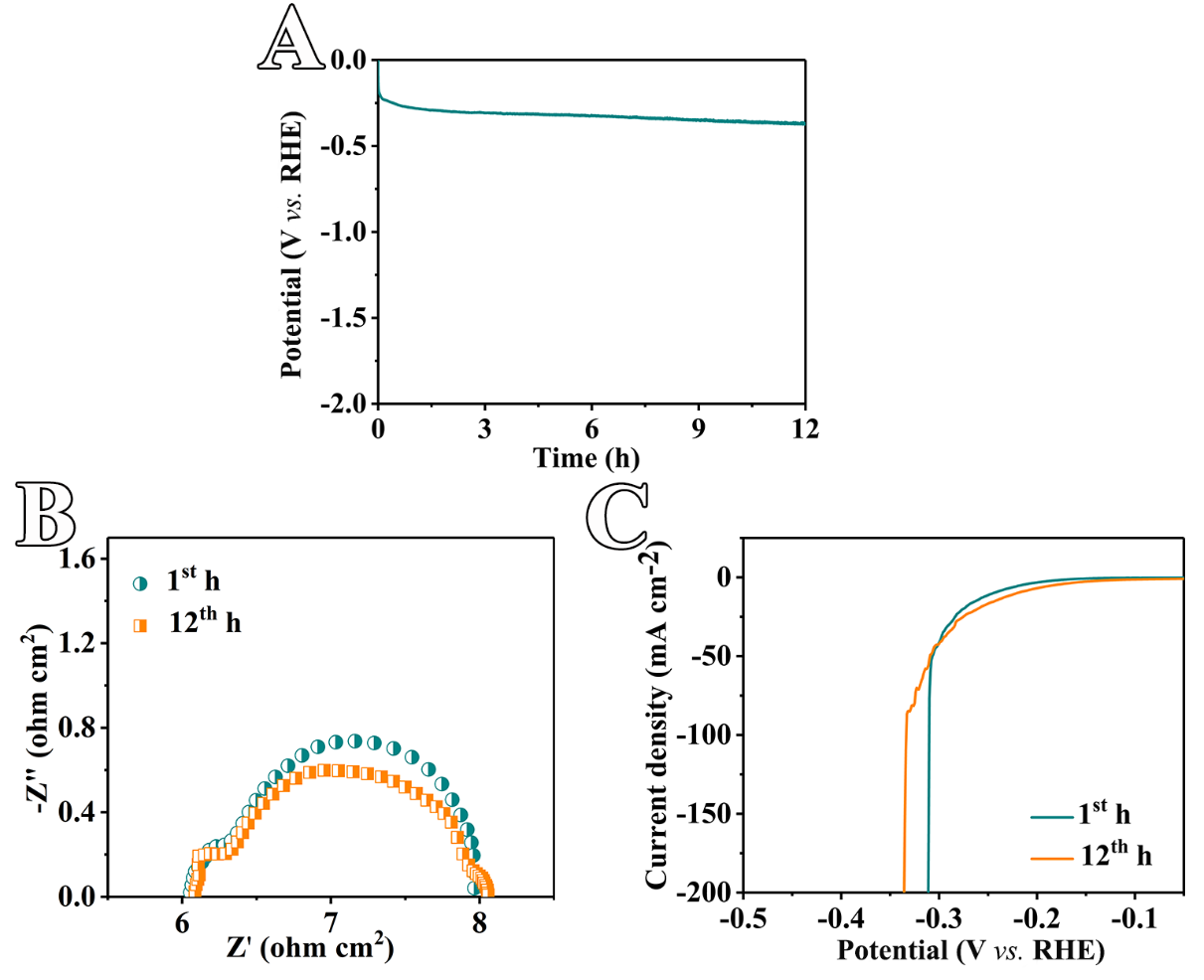


**Figure S29.** Stability performance of NiMn-MOF in 3 wt% NaCl electrolyte. (A) Stability test for 12 hrs; (B) EIS plots of NiMn-MOF before and after stability testing; (C) LSVs before and after stability testing. Remarkably, NiMn-MOF electrode shows excellent electrochemical durability as conﬁrmed by chronoamperometry (10 mA cm^-2^ for 12 hrs, panel A), EIS (panel B), and LSVs (panel C) before and after testing.


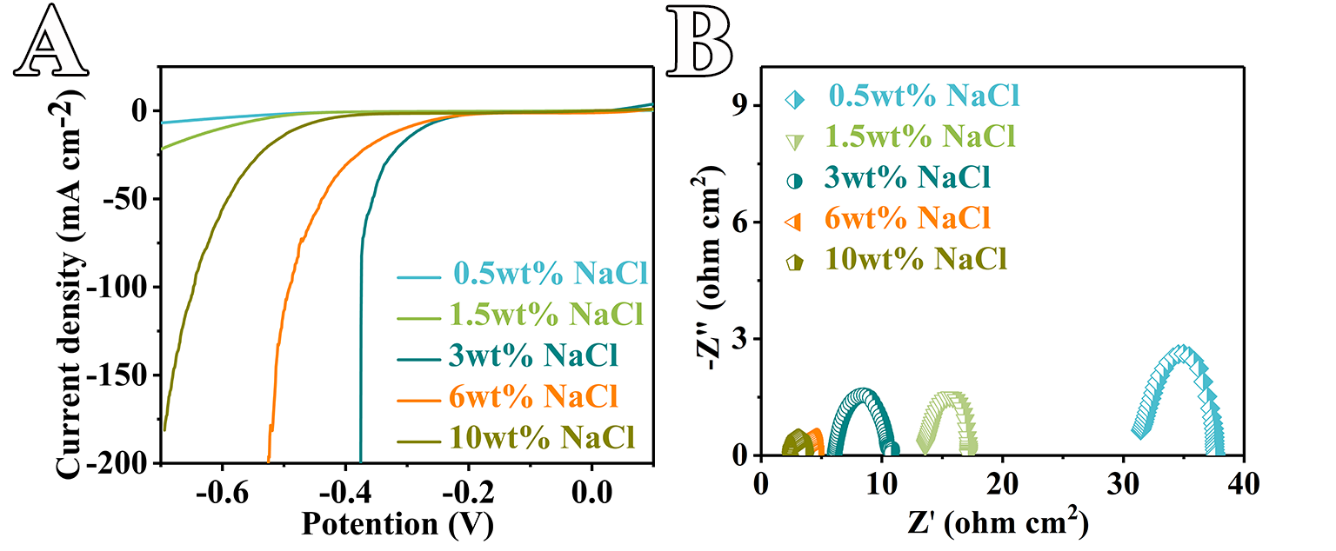


**Figure S30.** (A) LSVs and (B) EIS plots of the HER promoted by the NiMn-MOF electrode in NaCl electrolyte of different concentrations.

**Supplementary note.**

The effect of NaCl solution concentration (0.5, 1.5, 3, 6, and 10 wt%) on HER performance was studied. According to EIS plots (Figure. S30B, Table S5), the R_s_ of electrochemical system gradually decreases from 31.4 (0.5 wt%), 13.6 (1.5 wt%), 6.1 (3 wt%) and 3.6 (6 wt%) to 2.4 ohm cm^2^ (10 wt%), which indicates increase of ionic conductivity of NaCl electrolytes. However, LSVs shows a different trend from EIS results (Figure. S30A, Table S6). The concentration of NaCl soilution ranges from 0.5 to 10 wt%, the minimum overpotential of 277 mV is required at 3% NaCl to afford 10 mA cm^-2^ current density, which is much lower than 0.5 wt% (793 mV), 1.5 wt% (602 mV), 6 wt% (305 mV) and 10 wt% (479 mV, Figure. S30A, Table S6). We rationalize one possible explanation for performance degradation at high NaCl concentration: the excessively high ion concentration could prevent ionization of water molecules; and consequently, extra energy is required to promote dissociation of the electrolyte, leading to higher overpotential for HER.


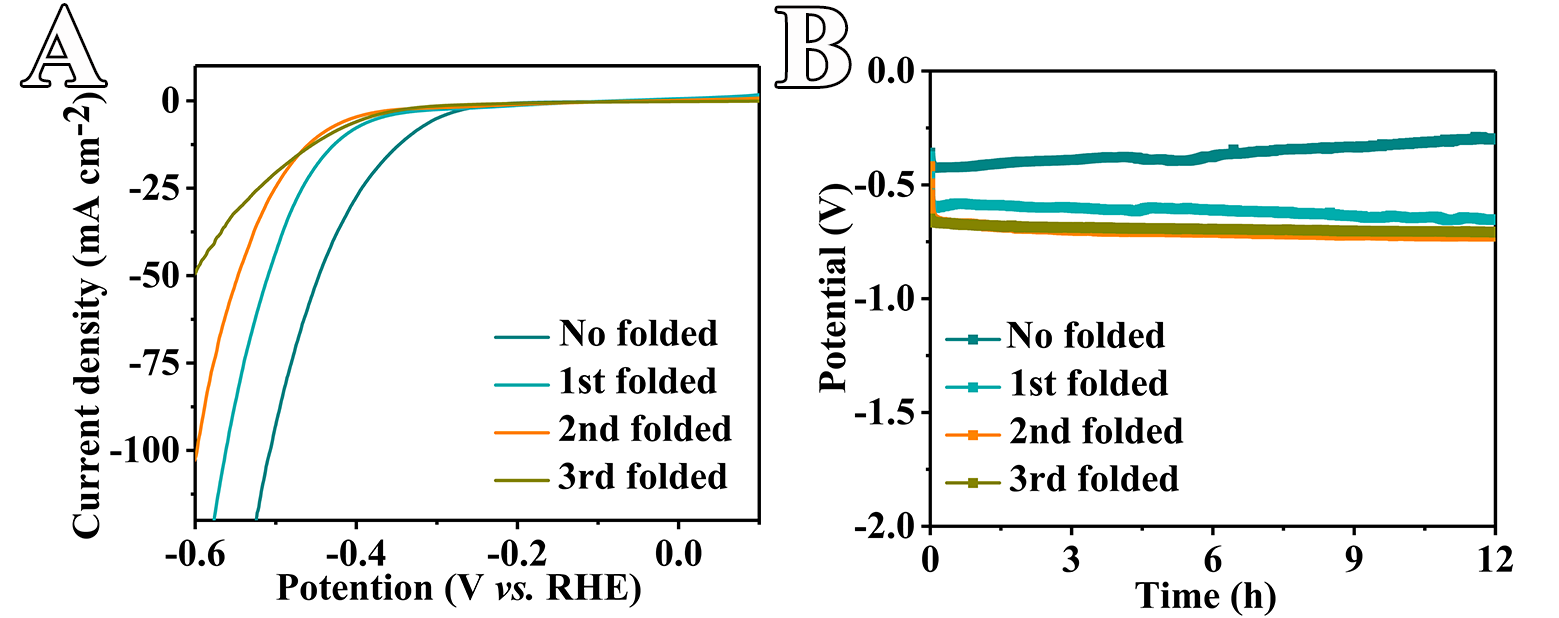


**Figure S31.** (A) LSVs and (B) chronoamperometric tests of bulk NiMn-MOF with different folding times in natural seawater.


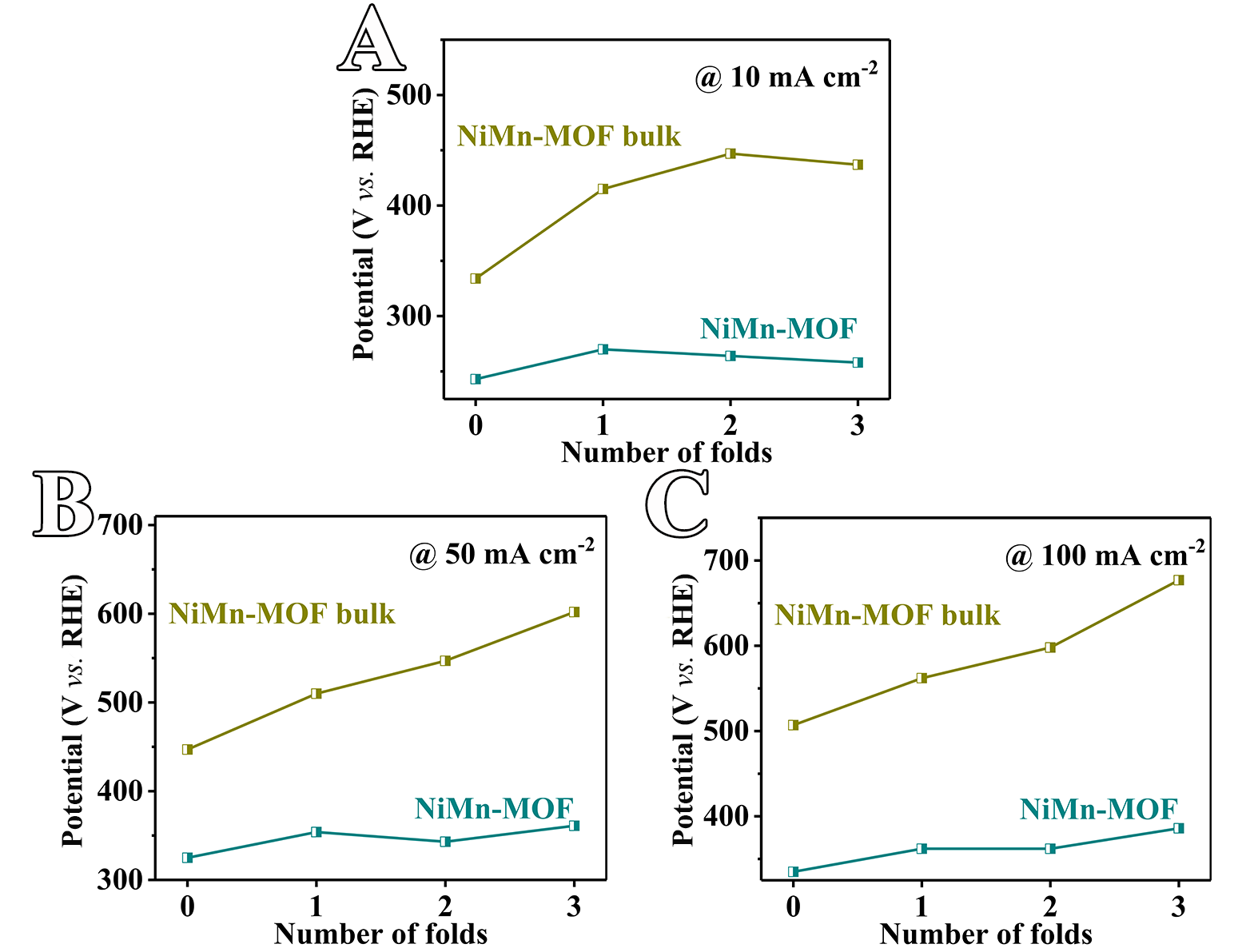


**Figure S32.** Plots showing the applied potentials for superplastic NiMn-MOF and bulk one with different electrode folds in natural seawater.


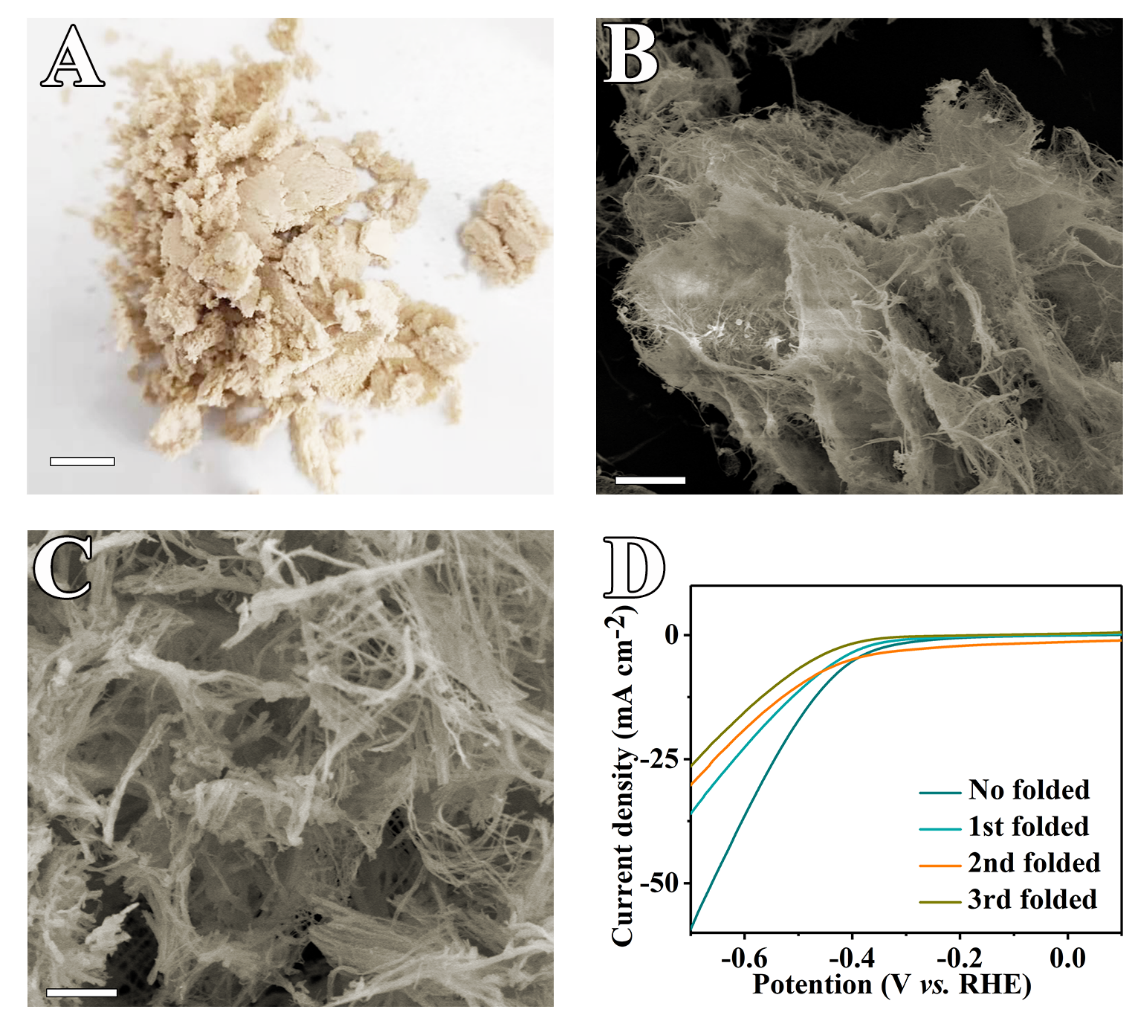


**Figure S33.** Morphological characterization and performance of S-NiMn-MOF. (A) an optical image (scale bars: 1 cm); (B, C) SEM images (scale bars for B, and C are 10 μm and 2 μm); (D) LSVs curves with different folding times in natural seawater.

**Supplementary note.**

As shown in Figure S33A, shorter NiMn-MOF nanobelts (S-NiMn-MOF) cannot form aerogels through a similar preparation method with that of superplastic NiMn-MOF aerogels. The SEM images shows that the length of S-NiMn-MOF is only a few tens of microns, so it can^’^t move along the direction of the ice template nor cross-link to form a three-dimensional network (Figure S33B, C). Further, the S-NiMn-MOF electrode was prepared using the same method with that of the NiMn-MOF powder electrode. As shown in Figure S33D, non-superplastic S-NiMn-MOF demands an overpotential of 450 mV to reach 10 mA·cm^-2^, which is much lower than that of superplastic NiMn-MOF aerogels. While the S-NiMn-MOF displays rapid decay of HER activity with folding, indicating that short S-NiMn-MOF nanobelts are unfavorable for formation of superplastic electrodes and thus exhibits decreased electrocatalytic performance.





**Figure S34.** Chronoamperometric testing for 30 hrs at -0.25 V (*vs.* RHE) in natural seawater, the inset shows EIS plots before and after testing.


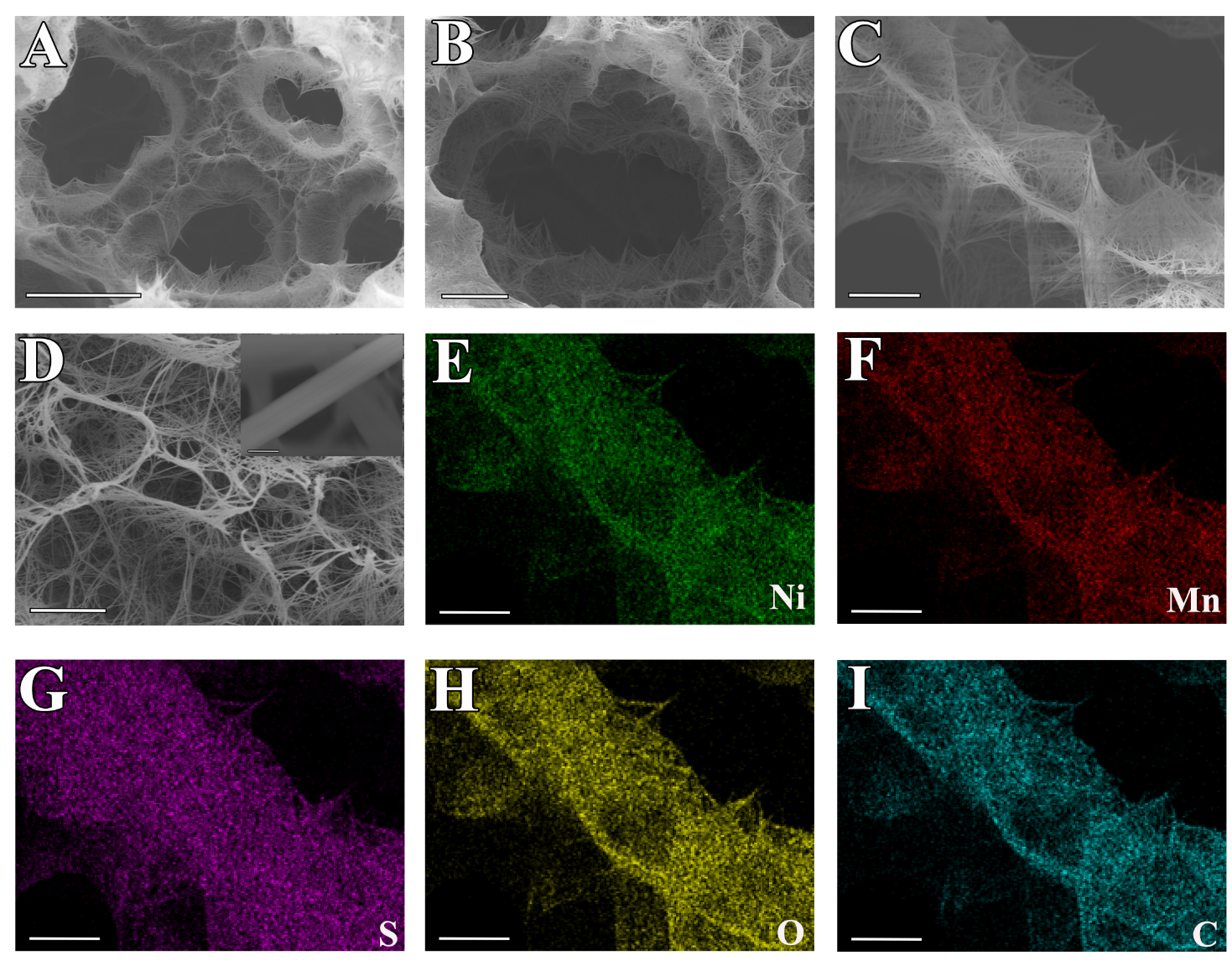


**Figure S35.** Morphological characterization of superplastic NiMn-MOF before electrochemical cycling. (A-D) SEM images (scale bars for A, B, C and D are 200 μm, 100 μm, 50 μm and 20 μm); inset of (D) is SEM image (scale bars: 300 nm); (E-I) SEM elemental mappings of Ni, Mn, S, O, C (scale bars: 50 μm).


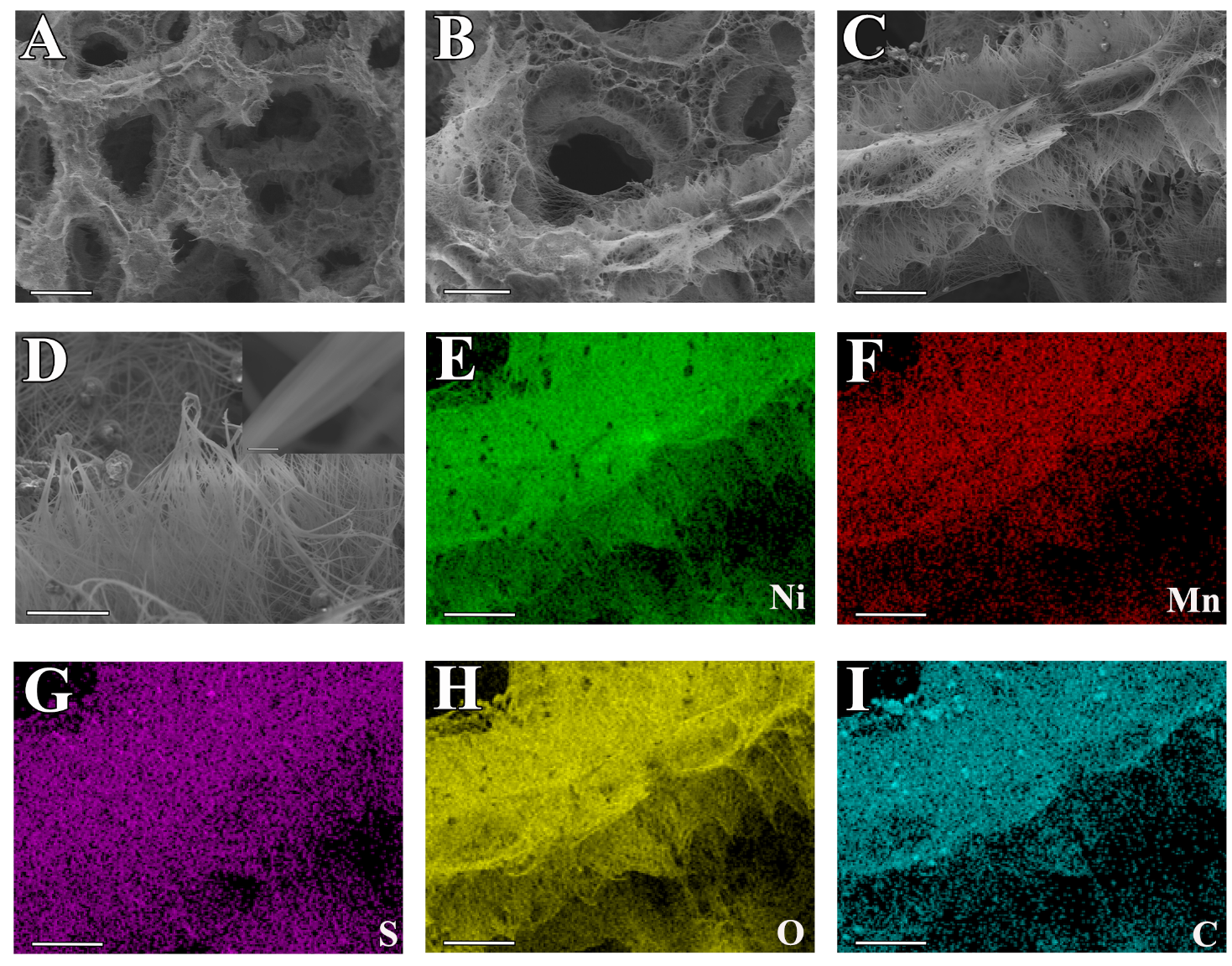


**Figure S36.** Morphological characterization of superplastic NiMn-MOF after electrochemical cycling. (A-D) SEM images (scale bars for A, B, C and D are 200 μm, 100 μm, 50 μm and 20 μm); inset of (D) is SEM image (scale bars: 300 nm); (E-I) SEM elemental mappings of Ni, Mn, S, O, C (scale bars: 50 μm).


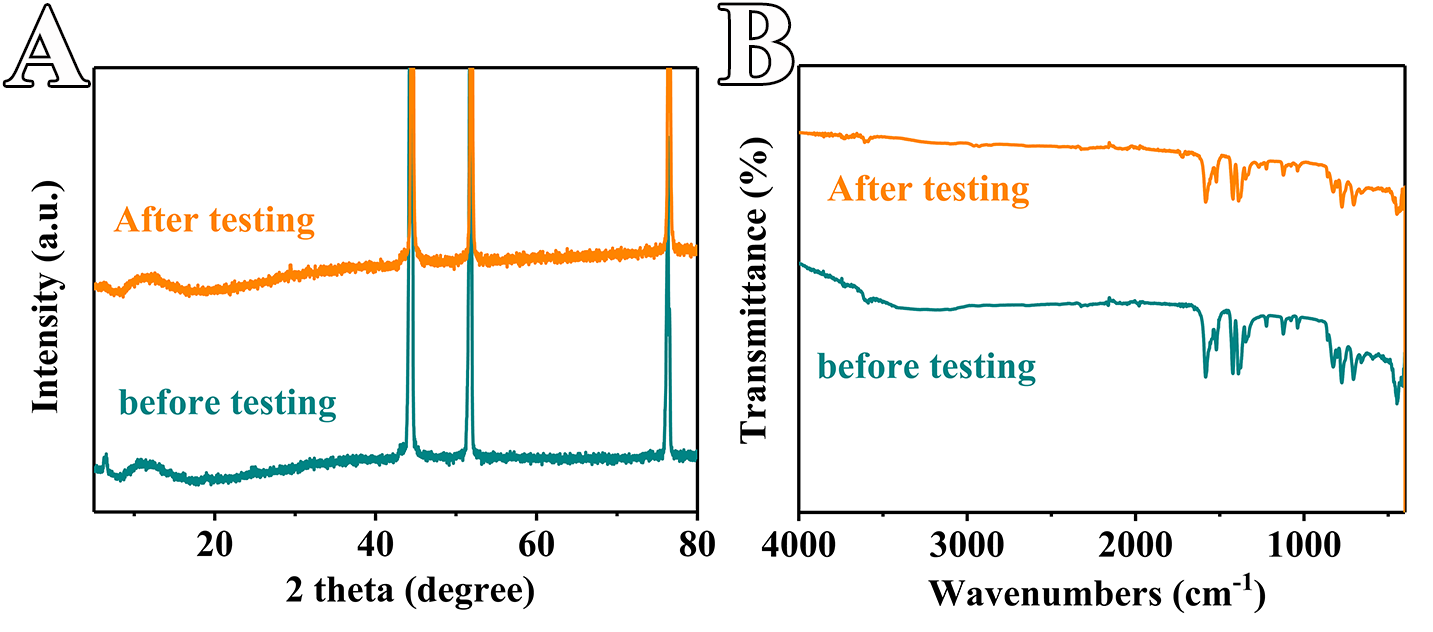


**Figure S37.** Structure characterizations of superplastic NiMn-MOF before and after electrochemical testing. (A) XRD patterns; (B) FT-IR spectra.


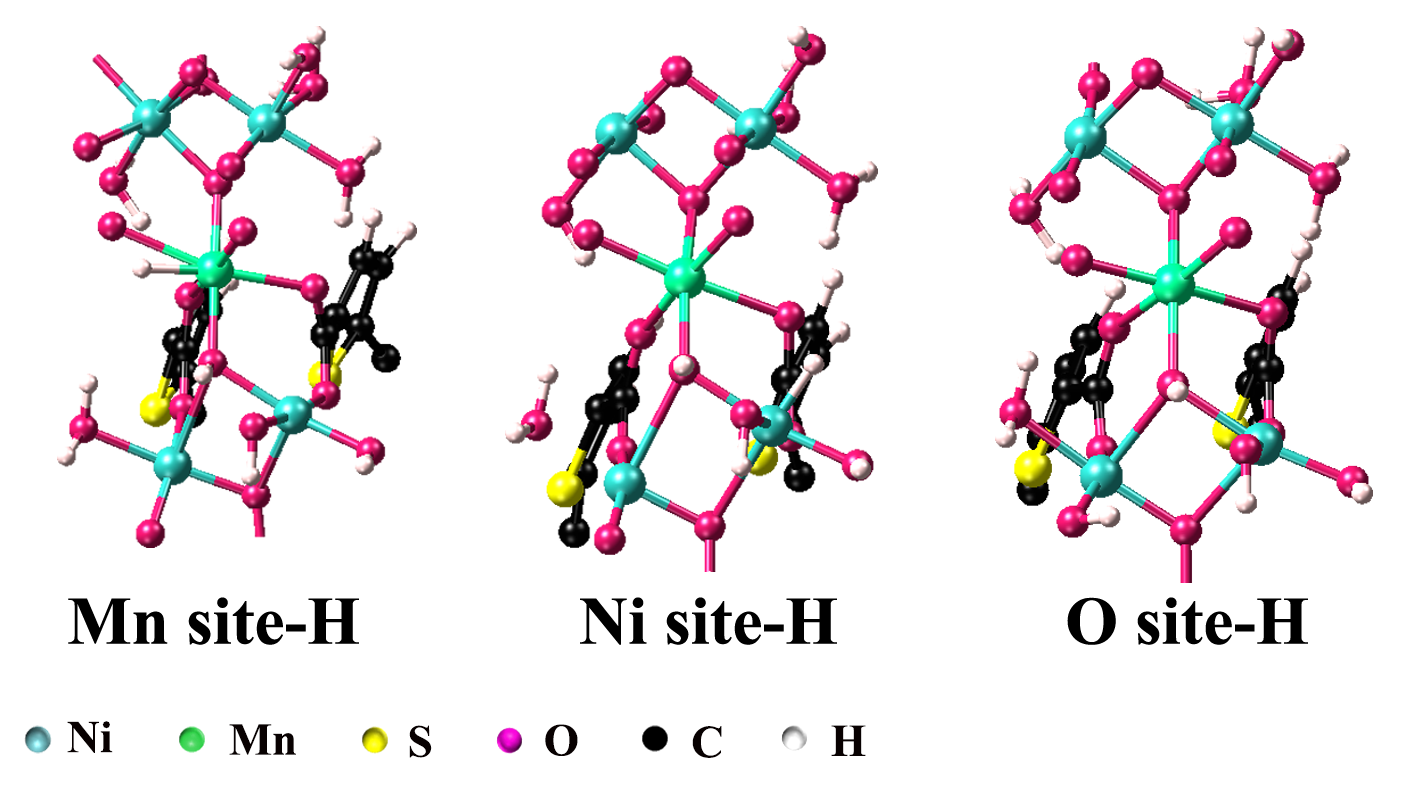


**Figure S38.** Optimized lattice structure for NiMn-MOF absorbed hydrogen atoms at different active sites. Color code: gray, nickel; green, manganese; yellow, sulfur; purple, oxygen; black, carbon; white, hydrogen.


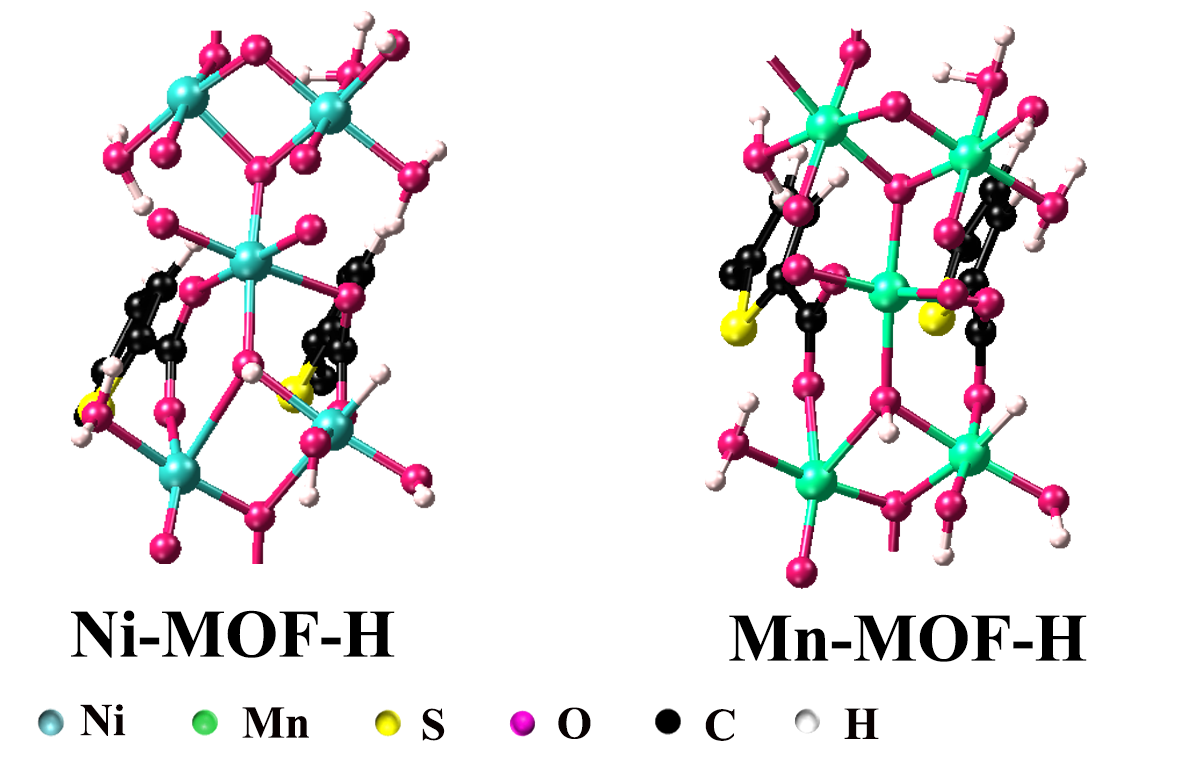


**Figure S39.** Optimized lattice structure for Ni-MOF and Mn-MOF absorbed hydrogen atoms. Color code: gray, nickel; green, manganese; yellow, sulfur; purple, oxygen; black, carbon; white, hydrogen.


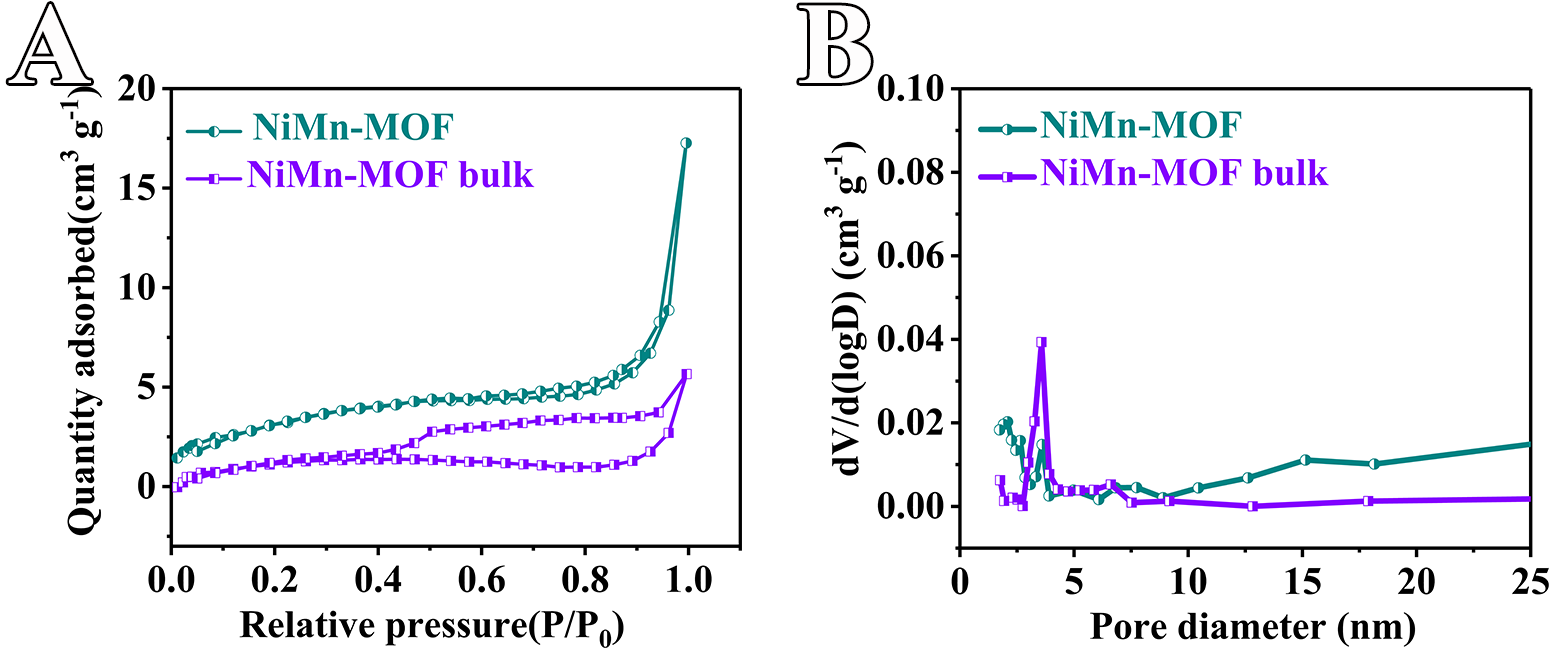


**Figure S40.** (A) N_2_ adsorption-desorption isotherms of superplastic NiMn-MOF and bulk powder; (B) BJH pore size distribution curves.

**Supplementary note.**

In terms of the N_2_ isotherm of bulk NiMn-MOF, the plots corresponding to type V adsorption-desorption profiles with H3 hysteresis loop. According to the literature,^14,15^ the H3 hysteresis loop is originated from narrow and slit pores formed by the aggregation of adjacent NiMn-MOF nanobelts. Notably, such a kind of hysteresis loop has not been observed in superplastic NiMn-MOF aerogels, indicating the individual nanobelts have been largely separated by ice-templated freeze-drying.


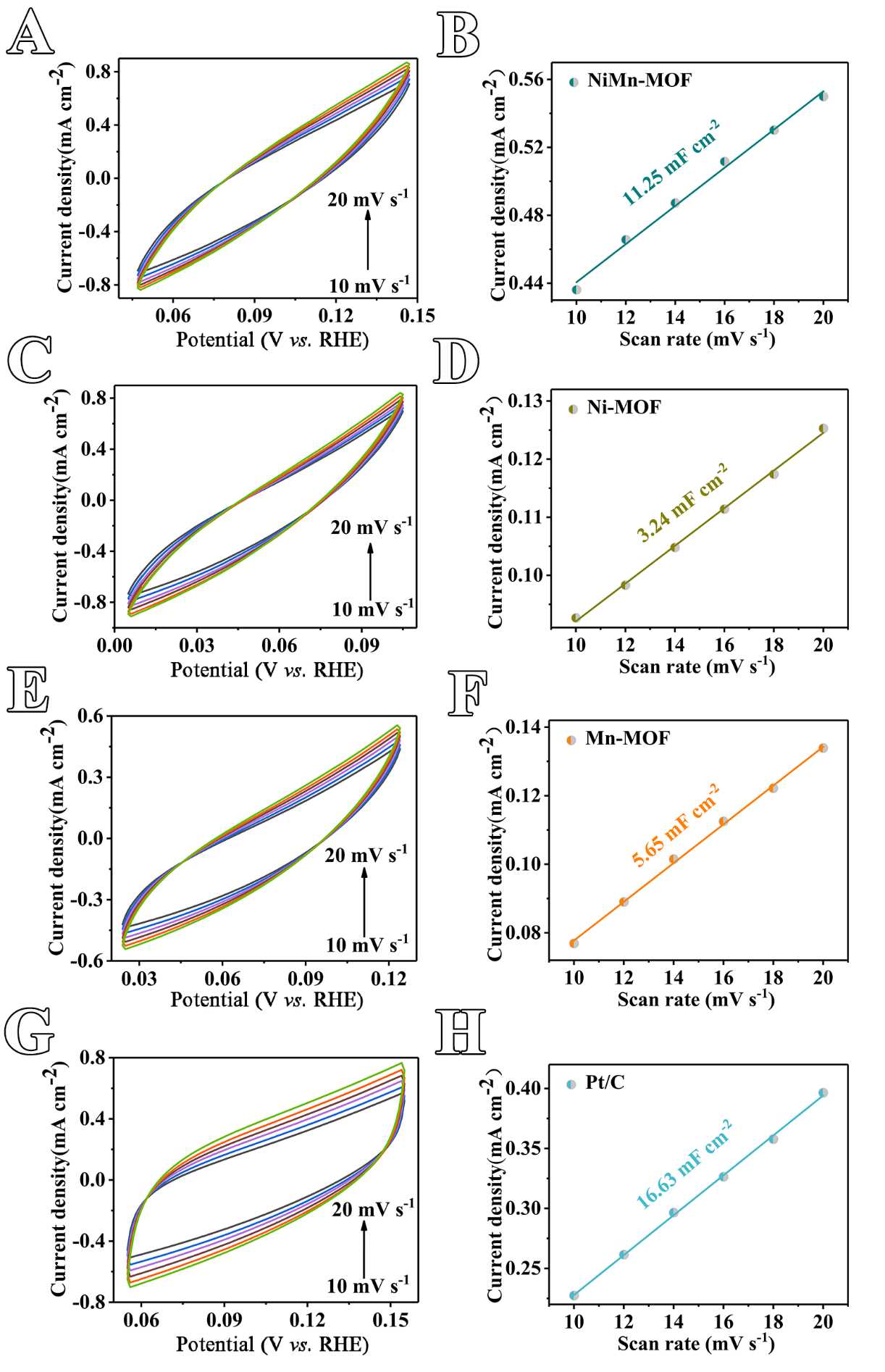


**Figure S41.** Cyclic voltammetry curve and corresponding capacitive plots for (A, B) NiMn-MOF, (C, D) Ni-MOF, (E, F) Mn-MOF and (G, H) Pt/C at different scan rates (10-20 mV s^-1^) in a 3 wt% NaCl solution.


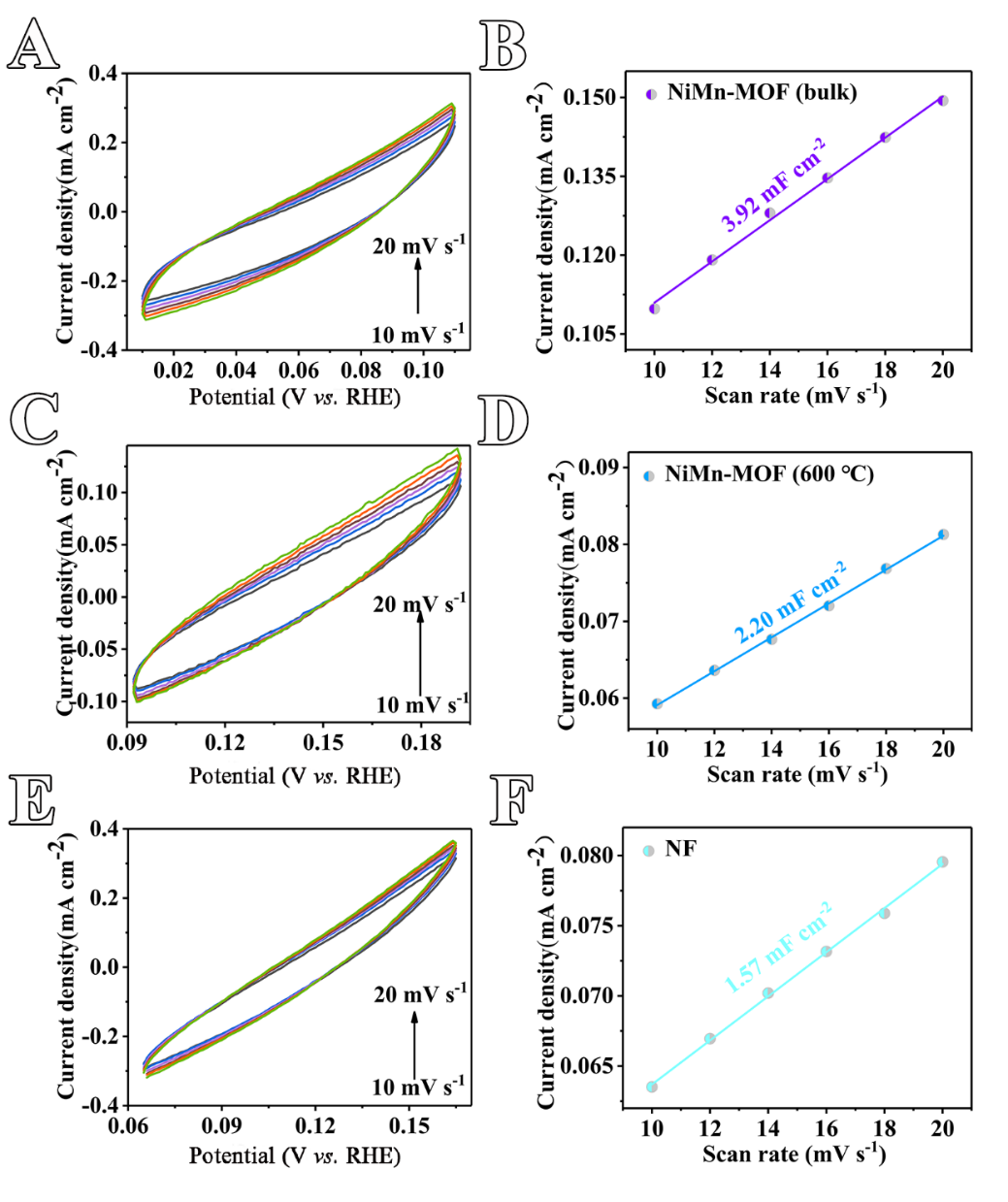


**Figure S42.** Cyclic voltammetry curves and corresponding capacitive plots for (A, B) bulk NiMn-MOF (powder), (C, D) NiMn-MOF treated at 600°C, and (E, F) NF at different scan rates (10-20 mV s^-1^) in 3 wt% NaCl solution.

**Supplementary note.**

As shown in Figures. S41,42, the C_dl_ value of NiMn-MOF is 11.25 mF cm^-2^, substantially larger than Ni-MOF (3.24 mF cm^-2^) and Mn-MOF (5.65 mF cm^-2^); consequently, the corresponding ECSA values are 321.43, 92.57, and 161.43. Furthermore, the LSVs were normalized to ECSA (Figure. S43). It shows that NiMn-MOF still exhibits higher HER activity than Ni- and Mn-MOFs, which suggests the strong synergy between Ni and Mn inside the material. The above result confirms that NiMn-MOF can provide enormous metal sites as catalytic centers for HER.


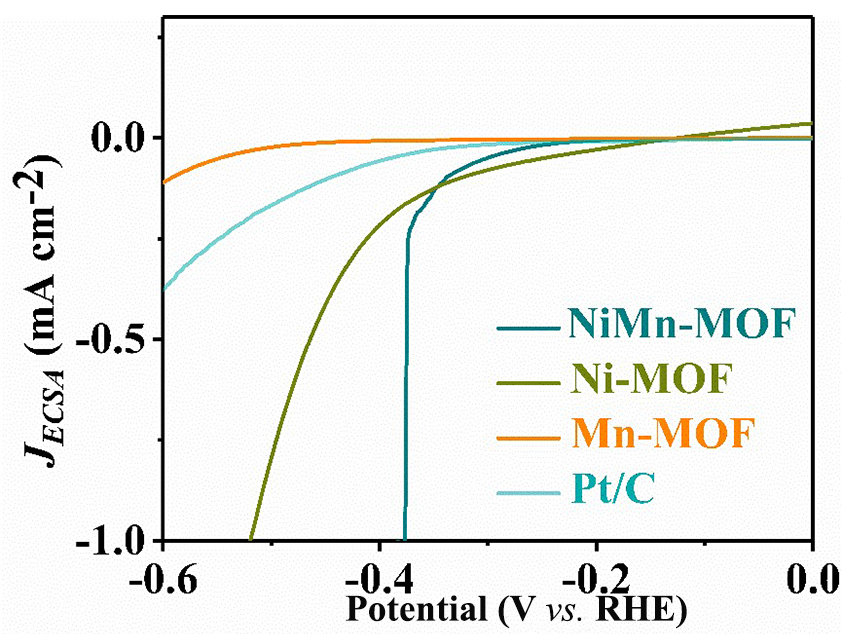


**Figure S43.** The LSV curves normalized by ECSA of different samples.

**Table S1.** ICP-OES analysis of NiMn-MOF and other comparison samples.

|  | **Content (wt.%)** | | | | |
| --- | --- | --- | --- | --- | --- |
|  |  | **Ni** | **Co** | **Mn** | **Total content** |
| 1 | Ni-MOF | 26.42% | - | - | 26.42% |
| 2 | Co-MOF | - | 27.30% | - | 27.30% |
| 3 | Mn-MOF | - | - | 24.71% | 24.71% |
| 4 | NiMn-MOF | 16.48% | - | 9.31% | 25.79% |
| 5 | NiCo-MOF | 13.46% | 13.37% | - | 26.83% |
| 6 | CoMn-MOF | - | 16.37% | 9.76% | 26.13% |
| 7 | NiCoMn-MOF | 9.16% | 8.94% | 6.67% | 24.77 |

**Table S2.** Electrical conductivity of NiMn-MOF and other comparison samples. Each sample was measured by repeatitieve six times.

|  | **Electrocal conductivity （S m^-1^）** | | | | |
| --- | --- | --- | --- | --- | --- |
|  | **Ni-MOF** | **NiMn- MOF (25% of Mn)** | **NiMn-MOF (67% of Mn)** | **NiMn- MOF (80% of Mn)** | **Mn- MOF** |
| 1 | 0.25 | 0.61 | 2.22 | 1.52 | 0.42 |
| 2 | 0.19 | 0.63 | 2.44 | 1.45 | 0.62 |
| 3 | 0.28 | 0.94 | 2.36 | 1.46 | 0.66 |
| 4 | 0.22 | 0.63 | 1.45 | 1.83 | 0.57 |
| 5 | 0.19 | 0.79 | 2.10 | 1.55 | 0.64 |
| 6 | 0.27 | 0.75 | 1.97 | 1.76 | 0.86 |
| Average | 0.23 | 0.73 | 2.09 | 1.60 | 0.63 |

**Table S3.** Average mass loadings of NiMn-MOF catalysts on nickel foam substrates.

| ***m*(nickel foam)/ g** | ***m*(NiMn-MOF)/ g** | **Area/cm^-2^** | ***Mass loading* /****mg cm^-2^** |
| --- | --- | --- | --- |
| 0.20201 | 0.20580 | 5.7 | 0.66491 |
| 0.19784 | 0.19974 | 5.7 | 0.33333 |
| 0.19651 | 0.19914 | 5.7 | 0.45614 |
| Average |  |  | 0.48 |

**Table S4.** Brunauer-Emmett-Teller (BET) surface area of NiMn-MOF and its powder counterpart.

| **Sample** | **BET specific surface area / m^2^ g^-1^** | **BJH volume of pores /** **cm^3^ g^-1^** |
| --- | --- | --- |
| **NiMn-MOF** | 11.8 | 0.013 |
| **NiMn-MOF (powder)** | 4.6 | 0.005 |

**Table S5.** Comparison of the HER activities for NiMn-MOF with other electrocatalysts in 3 wt% NaCl solution.

| **Sample** | **η_10_ (mV)** | **η_50_ (mV)** | **η_100_ (mV)** | **η_200_ (mV)** | **Tafel slope**  **(mV dec^-1^)** | **R_ct_ (Ω)** | **C_dl_**  **(mF cm^-2^)** |
| --- | --- | --- | --- | --- | --- | --- | --- |
| NiMn-MOF | 277 | 356 | 373 | 376 | 115 | 3.9 | 11.25 |
| Ni-MOF | 359 | 473 | 504 | 516 | 177 | 4.9 | 3.24 |
| NiMn-MOF (25%) | 483 | 579 | 606 | - | [184](#_ENREF_1) | 5.5 | - |
| NiMn-MOF (80%) | 396 | 513 | 573 | 625 | [163](#_ENREF_2) | 6.7 | - |
| Mn-MOF | 561 | - | - | - | [154](#_ENREF_3) | 7.1 | 5.65 |
| NiMn-MOF bulk | 327 | 447 | 483 | - | [168](#_ENREF_4) | 3.9 | 3.92 |
| NiMn-MOF (600 ^o^C) | 448 | 538 | 554 | - | [170](#_ENREF_5) | 1.7 | 2.2 |
| Pt/C | 324 | 452 | 526 | 616 | [72](#_ENREF_8) | 2.2 | 16.63 |

**Table S6.** Comparison of the HER activities for NiMn-MOF in electrolytes with different concentration.

| **Concentration of NaCl**  **(wt%)** | **η_10_**  **(mV)** | **η_50_**  **(mV)** | **η_100_**  **(mV)** | **η_200_**  **(mV)** | **R_s_**  **(Ω)** |
| --- | --- | --- | --- | --- | --- |
| 0.5 | 793 | - | - | - | 31.4 |
| 1.5 | 602 | 885 | - | - | 13.6 |
| 3 | 227 | 356 | 374 | 376 | 6.1 |
| 6 | 305 | 439 | 491 | 526 | 3.6 |
| 10 | 479 | 592 | 647 | - | 2.4 |

**Table S7.** Comparison of the HER activities for NiMn-MOF with different folding times in natural seawater.

| **Folding times** | **η_10_**  **(mV)** | **η_50_**  **(mV)** | **η_100_**  **(mV)** | **η_200_**  **(mV)** | **Tafel slope (mV dec^-1^)** | **R_ct_**  **(Ω)** |
| --- | --- | --- | --- | --- | --- | --- |
| No folded | 243 | 325 | 335 | 341 | 126 | 2.2 |
| 1^st^ folded | 270 | 354 | 362 | 364 | 139 | 2.1 |
| 2^nd^ folded | 264 | 343 | 362 | 372 | [121](#_ENREF_1) | 1.4 |
| 3^rd^ folded | 258 | 361 | 386 | 394 | [163](#_ENREF_2) | 1.9 |

**Table S8.** Comparison of the HER activities for bulk NiMn-MOF with different folding times in natural seawater.

| **Folding times** | **η_10_**  **(mV)** | **η_50_**  **(mV)** | **η_100_**  **(mV)** |
| --- | --- | --- | --- |
| No folded | 334 | 447 | 507 |
| 1^st^ folded | 415 | 510 | 562 |
| 2^nd^ folded | 447 | 547 | 598 |
| 3^rd^ folded | 437 | 602 | 677 |

**Table S9.** Comparison of the HER activities for NiMn-MOF with recently reported electrocatalysts in neutral electrolytes.

| **Catalyst** | **η_10_(mV)** | **η_50_(mV)** | **η_100_(mV)** | **η_200_(mV)** | **Mass loading** | **Electrolyte** | **Reference** |
| --- | --- | --- | --- | --- | --- | --- | --- |
| NiMn-MOF | 243 | 325 | 335 | 341 | 0.48 mg cm^-2^ | seawater | This work |
| Co_0.31_Mo_1.69_C/MXene/NC | 262 | 410 | unavailable | unavailable | 0.4 mg cm^-2^ | seawater | [^16^](#_ENREF_8) |
| CoMoP@C | 448 | 590 | unavailable | unavailable | 0.29 mg cm^-2^ | seawater | ^17^ |
| Co-N-C nanotubes | 680 | unavailable | unavailable | unavailable | 0.354 mg cm^-2^ | seawater | [^18^](#_ENREF_11) |
| RuCo/Ti | 387 | 665 | 880 | 1350 | - | seawater | [^19^](#_ENREF_12) |
| PtNi_5_ | 800 | 1025 | 1300 | unavailable | 0.398 mg cm^-2^ | seawater | [^20^](#_ENREF_13) |
| Mo_5_N_6_ | 258 | 300@20 mA cm^-2^ | unavailable | unavailable | 0.4 mg cm^-2^ | seawater | [^21^](#_ENREF_14) |

**Table S10.** The concentration for Ni and Mn in the electrolyte in the presence of NiMn-MOF after stability testing and without applied HER overpotential.

|  | **Sample** | **Concentration (mg L^-1^)** | | |
| --- | --- | --- | --- | --- |
|  |  | | **Ni** | **Mn** |
| 1 | NiMn-MOF after the stability test for 12 hrs | | 0.1372 | 0.0053 |
| 2 | NiMn-MOF after immersion 12 hrs | | 0.1933 | 0.0074 |

**Supplementary References**

(1) Feng, D.; [Lei](https://www.nature.com/articles/s41560-017-0044-5#auth-Ting-Lei), T.; Lukatskaya[, M. R.; Lukatskaya](https://www.nature.com/articles/s41560-017-0044-5#auth-Maria_R_-Lukatskaya), J. P.; Huang[, Z. H.; Huang](https://www.nature.com/articles/s41560-017-0044-5#auth-Zhehao-Huang), M. L.; [Leo](https://www.nature.com/articles/s41560-017-0044-5#auth-Leo-Shaw), S.; Chen[, A. A.; Yakovenko](https://www.nature.com/articles/s41560-017-0044-5#auth-Andrey_A_-Yakovenko), A. K.; Xiao[,](https://www.nature.com/articles/s41560-017-0044-5#auth-Jianping-Xiao) J. P.; [Kurt](https://www.nature.com/articles/s41560-017-0044-5#auth-Kurt-Fredrickson), F.; [Jeffrey](https://www.nature.com/articles/s41560-017-0044-5#auth-Jeffrey_B_-Tok), B. T.; Zou, X. D.; Cui, Y.; Bao, Z. N. Robust and conductive two-dimensional metal-organic frameworks with exceptionally high volumetric and areal capacitance. *Nat. Energy* **2018**, *3*, 30-36.

(2) Chen, J.; Sheng, K.; Luo, P.; Li, C.; Shi, G. Graphene hydrogels deposited in nickel foams for high-rate electrochemical capacitors. *Adv. Mater.* **2012**, *24*, 4569-4573.

(3) Chen, S.; Duan, J.; Jaroniec, M.; Qiao, S. Z. Three-dimensional N-doped graphene hydrogel/NiCo double hydroxide electrocatalysts for highly efficient oxygen evolution. *Angew. Chem., Int. Ed.* **2013**, *52*, 13567-13570.

(4) Luo, Y.; Chen, G. F.; Ding, L.; Chen, X.; Ding, L. X.; Wang, H. Efficient electrocatalytic N_2_ fixation with MXene under ambient conditions. *Joule* **2019**, *3*, 279-289.

(5) Kresse, G.; Furthmüller, J. Efficiency of ab-initio total energy calculations for metals and semiconductors using a plane-wave basis set. *Com. Mater. Sci.* **1996**, *6*, 15-50.

(6) Kresse, G.; Furthmuller, J. Efficient iterative schemes for ab initio total-energy calculations using a plane-wave basis set. *Phys. Rev. B.* **1996**, *54*, 11169-11186.

(7) Perdew, J. P.; Burke, K.; Ernzerhof, M. Generalized gradient approximation made simple. *Phys. Rev. Lett.* **1996**, *77*, 3865-3868.

(8) Wang, L.; Maxisch, T.; Ceder, G. Oxidation energies of transition metal oxides within the GGA+U framework. *Phys. Rev. B.* **2006**, *73*, 195107.

(9) Zhao, H. J.; Liu, X. Q. Chen, X. M.; Bellaiche, L. Effects of chemical and hydrostatic pressures on structural, magnetic, and electronic properties of R_2_NiMnO_6_ (R=rare-earthion) double perovskites. *Phys. Rev. B.* **2014**, *90*, 195147.

(10) Xiao, X.; Li, Q.; Yuan, X. Y.; Xu, Y. X.; [Zheng](https://onlinelibrary.wiley.com/action/doSearch?ContribAuthorStored=Zheng%2C+Mingbo), M. B.; [Pang](https://onlinelibrary.wiley.com/action/doSearch?ContribAuthorStored=Pang%2C+Huan), H. Ultrathin nanobelts as an excellent bifunctional oxygen catalyst: insight into the subtle changes in structure and synergistic effects of bimetallic metal-organic framework. *Small Methods* **2018**, *2*, 1800240.

(11) Du, J.; Wang, R.; Lv, Y. R.; Wei, Y. L.; Zang, S. Q. One-step MOF-derived Co/Co_9_S_8_ nanoparticles embedded in nitrogen, sulfur and oxygen ternary-doped porous carbon: an efficient electrocatalyst for overall water splitting. *Chem. Commun.* **2019**, *55*, 3203-3206.

(12) Fei, H. L.; Liu, X.; Li, Z. W.; Feng, W. J. Synthesis of manganese coordination polymer microspheres for lithium-ion batteries with good cycling performance. *Electrochim. Acta.* **2015** *174*, 1088-1095.

(13) Marques, L. F.; [Santos,](https://www.sciencedirect.com/science/article/pii/S0020169316303656#!) H. P.; [Correa](https://www.sciencedirect.com/science/article/pii/S0020169316303656#!), C. C.; [Resende,](https://www.sciencedirect.com/science/article/pii/S0020169316303656#!) A. L. C.; [Silva](https://www.sciencedirect.com/science/article/pii/S0020169316303656#!), R. R. D.; [Ribeiro](https://www.sciencedirect.com/science/article/pii/S0020169316303656#!), S. J. L.; [Machado](https://www.sciencedirect.com/science/article/pii/S0020169316303656#!) F. C. Construction of a series of rare earth metal-organic frameworks supported by thiophenedicarboxylate linker: Synthesis, characterization, crystal structures and near-infrared/visible luminescence. *Inorg. Chim. Acta.* **2016**, *451*, 41-51.

(14) Kruk, M.; Jaroniec, M. Application of large pore MCM-41 molecular sieves to improve pore size analysis using nitrogen adsorption measurements. *Langmuir* **1997**, *13*, 6267-6273.

(15) TillotsonL, T.M.; Hrubesh, W. Transparent ultralow-density silica aerogels prepared by a two-step sol-gel process. *J. Non-Cryst. Solids* **1992**, *145*, 44-50.

(16) Wu, X. H.; [Zhou](https://onlinelibrary.wiley.com/action/doSearch?ContribAuthorStored=Zhou%2C+Si), S.; [Wang](https://onlinelibrary.wiley.com/action/doSearch?ContribAuthorStored=Wang%2C+Zhiyu), Z. Y.; [Liu](https://onlinelibrary.wiley.com/action/doSearch?ContribAuthorStored=Liu%2C+Junshan), J. S.; [Pei](https://onlinelibrary.wiley.com/action/doSearch?ContribAuthorStored=Pei%2C+Wei), W.; [Yang](https://onlinelibrary.wiley.com/action/doSearch?ContribAuthorStored=Yang%2C+Pengju), P. J.; [Zhao](https://onlinelibrary.wiley.com/action/doSearch?ContribAuthorStored=Zhao%2C+Jijun), J. J.; [Qiu](https://onlinelibrary.wiley.com/action/doSearch?ContribAuthorStored=Qiu%2C+Jieshan), J. S. Engineering multifunctional collaborative catalytic interface enabling efficient hydrogen evolution in all pH range and seawater. *Adv. Energy Mater.* **2019**, *9*, 1901333.

(17) Ma, Y. Y.; Wu, C. X.; Feng, X. J.; Tan, H. Q.; Yan, L. K.; Liu, Y.; Kang, Z. H.; Wang, E. B.; Li, Y. G. Highly efficient hydrogen evolution from seawater by a low-cost and stable CoMoP@C electrocatalyst superior to Pt/C. *Energ. Environ. Sci.* **2017***, 10*, 788-798.

(18) Gao, S.; [Li](https://pubs.rsc.org/en/results?searchtext=Author%3AGuo-Dong%20Li), G. D.; [Liu](https://pubs.rsc.org/en/results?searchtext=Author%3AYipu%20Liu), Y. P.; [Chen](https://pubs.rsc.org/en/results?searchtext=Author%3AHui%20Chen), H.; [Feng](https://pubs.rsc.org/en/results?searchtext=Author%3ALiang-Liang%20Feng), L. L.; [Wang](https://pubs.rsc.org/en/results?searchtext=Author%3AYun%20Wang), Y.; [Yang](https://pubs.rsc.org/en/results?searchtext=Author%3AMin%20Yang), M.; [Wang](https://pubs.rsc.org/en/results?searchtext=Author%3ADejun%20Wang), D. J.; [Wang](https://pubs.rsc.org/en/results?searchtext=Author%3AShan%20Wang), S.; [Zou](https://pubs.rsc.org/en/results?searchtext=Author%3AXiaoxin%20Zou), X. X.; Electrocatalytic H_2_ production from seawater over Co, N-codoped nanocarbons. *Nanoscale* **2015***, 7*, 2306-2316.

(19) Niu, X. M.; Tang, Q. W.; He, B. L.; Yang, P. Z. Robust and stable ruthenium alloy electrocatalysts for hydrogen evolution by seawater splitting. *Electrochim. Acta.* **2016**, *208*, 180-187.

(20) Zheng, J. Seawater splitting for high-efficiency hydrogen evolution by alloyed PtNi_x_ electrocatalysts. *Appl. Surf. Sci.* **2017***, 413,* 360-36.

(21) Jin, H. Single-crystal nitrogen-rich two-dimensional Mo_5_N_6_ nanosheets for efficient and stable seawater splitting. *ACS Nano* **2018***, 12*, 12761-12769.
